# Insights into the genome and secretome of *Fusarium metavorans* DSM105788 by cultivation on agro-residual biomass and synthetic nutrient sources

**DOI:** 10.1186/s13068-021-01927-9

**Published:** 2021-03-20

**Authors:** Sophie C. Brandt, Hévila Brognaro, Arslan Ali, Bernhard Ellinger, Katharina Maibach, Martin Rühl, Carsten Wrenger, Hartmut Schlüter, Wilhelm Schäfer, Christian Betzel, Stefan Janssen, Martin Gand

**Affiliations:** 1 Faculty of Mathematics, Computer Science and Natural Science, Department of Biology, Biozentrum Klein Flottbek, Molecular Phytopathology, University of Hamburg, Ohnhorststr. 18, 22609 Hamburg, Germany; 2 Department of Parasitology, Institute of Biomedical Sciences, University of São Paulo, Av. Prof. Lineu Prestes, 1374, São Paulo, CEP 05508-000 Brazil; 3 Institute of Biochemistry and Molecular Biology, University of Hamburg, Martin Luther King Platz 6, 20146 Hamburg, Germany; 4 Dr. Panjwani Center for Molecular Medicine and Drug Research, International Center for Chemical and Biological Sciences, University of Karachi, University Road, Karachi, 75270 Pakistan; 5 Institute of Clinical Chemistry and Laboratory Medicine Diagnostic Center, Campus Research. Martinistr. 52, N27, 20246 Hamburg, Germany; 6 Fraunhofer Institute for Translational Medicine and Pharmacology ITMP, Department ScreeningPort, Schnackenburgallee 114, 22525 Hamburg, Germany; 7 Department Biology and Chemistry, Algorithmic Bioinformatics, Justus Liebig University Giessen, Heinrich-Buff-Ring 58, 35392 Gießen, Germany; 8 Department Biology and Chemistry, Institute of Food Chemistry and Food Biotechnology, Justus Liebig University Giessen, Heinrich-Buff-Ring 17, 35392 Gießen, Germany

**Keywords:** *Fusarium solani* species complex, *Fusarium metavorans*, Genome analysis, Secretome, Mass spectrometry, Proteomics, CAZyme analysis, Cellulose degradation, Residual biomass treatment

## Abstract

**Background:**

The transition to a biobased economy involving the depolymerization and fermentation of renewable agro-industrial sources is a challenge that can only be met by achieving the efficient hydrolysis of biomass to monosaccharides. In nature, lignocellulosic biomass is mainly decomposed by fungi. We recently identified six efficient cellulose degraders by screening fungi from Vietnam.

**Results:**

We characterized a high-performance cellulase-producing strain, with an activity of 0.06 U/mg, which was identified as a member of the *Fusarium solani* species complex linkage 6 (*Fusarium metavorans*), isolated from mangrove wood (FW16.1, deposited as DSM105788). The genome, representing nine potential chromosomes, was sequenced using PacBio and Illumina technology. In-depth secretome analysis using six different synthetic and artificial cellulose substrates and two agro-industrial waste products identified 500 proteins, including 135 enzymes assigned to five different carbohydrate-active enzyme (CAZyme) classes. The *F. metavorans* enzyme cocktail was tested for saccharification activity on pre-treated sugarcane bagasse, as well as untreated sugarcane bagasse and maize leaves, where it was complemented with the commercial enzyme mixture Accellerase 1500. In the untreated sugarcane bagasse and maize leaves, initial cell wall degradation was observed in the presence of at least 196 µg/mL of the in-house cocktail. Increasing the dose to 336 µg/mL facilitated the saccharification of untreated sugarcane biomass, but had no further effect on the pre-treated biomass.

**Conclusion:**

Our results show that *F. metavorans* DSM105788 is a promising alternative pre-treatment for the degradation of agro-industrial lignocellulosic materials. The enzyme cocktail promotes the debranching of biopolymers surrounding the cellulose fibers and releases reduced sugars without process disadvantages or loss of carbohydrates.

**Supplementary Information:**

The online version contains supplementary material available at 10.1186/s13068-021-01927-9.

## Background

Lignocellulosic biomass is the only sustainable source of organic carbon, offering a promising resource for the production of fuels, chemicals and carbon-based materials [1]. However, the use of lignocellulosic biomass must be considered in the context of sustainable agriculture to avoid competition with food and feed production [2]. Biotechnological approaches are therefore required to valorize non-edible biomass, focusing on abundant sources such as forestry and agricultural wastes [3]. Sugarcane is the dominant crop in tropical areas such as South America and South Asia [4], whereas maize dominates in sub-tropical and temperate regions such as North America and Northern Europe [5]. The widespread agricultural use of these two C4 crops generates large quantities of lignocellulosic biomass that can be valorized without compromising food/feed production.

Lignocellulosic biomass has a heterogeneous structure and composition dependent on the plant species [6–8]. The main component is cellulose, the most abundant polymer on earth, consisting of linear chains of several hundred to many thousand β-(1,4)-d-glucose units. The other components are hemicellulose, pectin, lignin and extractives, the latter comprising a diverse range of substances that impede the enzymatic hydrolysis of biomass [9, 10]. Hemicellulose, the second most abundant polymer in plant cell walls [11], features at least six different macromolecules with varying ratios of pentose (xylose and arabinose) and hexose (mostly mannose and glucose) residues [12]. Xylans have a linear backbone of β-(1,4)-linked β-d-xylopyranosyl residues, whereas glucuronoxylans feature substituted 4-*O*-methyl-α-d-glucuronopyranosyl units and acetyl groups, and arabinoxylans contain xylose substituted with α-l-arabinofuranosyl units [11]. Xyloglucans have a cellulose-like linear backbone of β-(1,4)-d-glucose with additional β-(1,6)-linked xylose sidechains, often capped with galactose and fucose [13]. Glucomannans have backbones of β-(1,4)-linked d-mannose and d-glucose, sometimes with branching β-(1,6)-glucosyl residues [14], but if α-(1,6)-linked galactose units are present the polymers are known as galactoglucomannans [15]. Pectin is a complex heteropolymer of covalently linked d-galacturonic acid and other residues, and is also a significant component of sugarcane and maize bagasse [16]. The main constituents are (1) homogalacturonan, comprising linear α-(1,4)-d-galactouronic acid chains with some esterified or *O*-acetylated modifications; (2) rhamnogalacturonan-I, comprising repeated disaccharides of galacturonic acid and C-3 or C-2 *O*-acetylated rhamnosyl residues, with linear or branched α-l-arabinofuranosyl and/or galactopyranosyl side chains on C-4; and (3) substituted galacturonans as linear and side chain residues (rhamnogalacturonan-II), resulting in 12 types of glycosyl units that form at least 22 types of glycosidic bonds [17].

The recalcitrance of lignocellulosic biomass in part reflects the complexity of the substrate, with complete hydrolysis requiring efficient enzymes for the digestion of cellulose as well as palettes of enzymes that can digest the components of hemicellulose [18] and pectin [19]. However, enzymatic hydrolysis is also impeded by the inaccessibility of the substrates, which can be addressed by physical and/or chemical pre-treatment. Such processes can generate inhibitors that limit the activity of cellulases and other enzymes, as well as toxic molecules such as furfurals, acetic acid, formic acid and lignin-derived phenolic compounds that interfere with fermentation [20]. The effect of biomass pre-treatment [21, 22] can therefore be improved by optimizing the enzymatic cocktails used to hydrolyze lignocellulosic biomass, tailoring them for the type of biomass and for the ability to tolerate inhibitors [1, 9, 10, 23]. Although the polysaccharide content of maize leaf and sugarcane culm cell walls is similar [24, 25], the cross-linking of polysaccharides and the interactions between polysaccharide and lignin/phenolic compounds differ, resulting in unique cell wall architectures. The physical and chemical characteristics of the biomass therefore reflect variations in the degree of cellulose polymerization, crystallinity, and lignin content, the hemicellulose and pectin content, and cell wall thickness [26].

Lignocellulosic biomass in nature is mainly decomposed by fungi, which are therefore promising candidates for the discovery of enzymes or enzyme cocktails for biomass degradation [27]. More than 5 million species of fungi have been described, and the number is likely to increase given that only 5% of species are formally classified [28, 29]. The subkingdom Dikarya consists of two phyla: Ascomycota, the largest phylum, commonly known as sac fungi [30], and Basidiomycota, the second largest phylum, commonly known as higher mushrooms or pillar fungi. The filamentous ascomycetes are ubiquitous and *Fusarium* is one of the most abundant genera in that phylum [31]. *Fusarium* species are frequently isolated from tropical, sub-tropical, and temperate environments, and less frequently from alpine habitats [32]. The genus *Fusarium* was first described at the beginning of the nineteenth century [33, 34]. Nine species have been described, including the easily recognized *Fusarium solani,* based on its striking morphology [35]. However, the current concept of *F. solani* is a species complex (FSSC) within the class Sordariomycetes, order Hypocreales, and the family Nectriaceae. The FSSC is thought to contain at least 60 phylogenetically distinct but closely related and morphologically similar species [36], and is allied with the sexual species *Nectria haematococca*. Robust classification within the FSSC and the genus *Fusarium* is achieved by analyzing polymorphisms in the genes encoding translation elongation factor 1α (TEF1) and the second largest subunit of RNA polymerase II (RPB2) as well as the internal transcribed spacer (ITS) together with 28S ribosomal RNA (ITS + 28S) [36–38]*.* Members of the FSSC collectively have a broad host range and can be found as soil-dwelling saprophytes, rhizosphere colonizers, or pathogens of pea, bean, potato, soybean, maize and many cucurbit plants, as well as animals including humans [39]. *Fusarium* sp. of the FSSC has 5–17 chromosomes, with a genome size of 40–54 Mbp and a GC content of ~ 50% [35, 40–42].

Our previously reported analysis of 295 fungal isolates, collected from different substrates and various environments in Vietnam, revealed their ability to degrade lipids, chitin, cellulose and xylan [43]. Six isolates were able to digest carboxymethylcellulose (CMC) with remarkable efficiency, two of which were *Fusarium* strains. We selected the most active member of FSSC linkage 6, isolated from dead mangrove wood, for further analysis. We characterized this strain as *F. metavorans* FW16.1 by analyzing its genome and secretome, leading to the identification of undiscovered lignocellulose degrading enzymes with the ability to convert sugarcane bagasse and maize leaves into fermentable sugars.

## Results

### Characterization, genomic analysis and phylogenetics of *F. metavorans* FW16.1

**Fig. 1:**
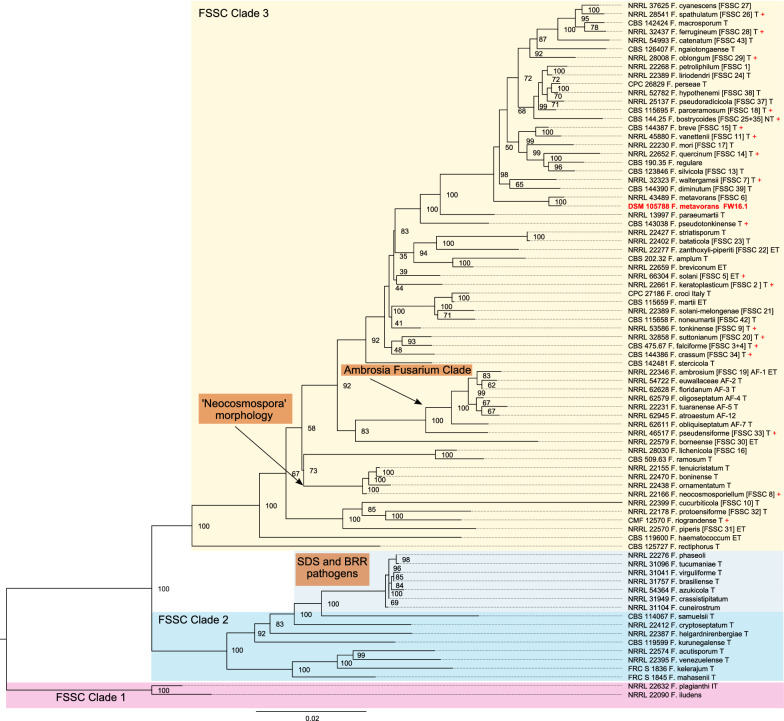
Phylogenetic tree of 79 *Fusarium* taxa plus FW16.1 estimated by partitioned maximum likelihood bootstrapping. Numbers at internal nodes indicate branch support based on 5000 data pseudo-replicates in IQ-TREE. The tree was rooted at NRRL 22,090 *F. iludens* and NRRL 22,632 *F. plagianthi*. The alignment holds 3209 columns and 1024 distinct patterns, of which 658 are parsimony-informative, 258 are singletons, and 293 are constant sites. FSSC numbers in brackets represent the ad hoc nomenclature previously used to distinguish species (10.1128/JCM.02371-07). T = ex-type strains; IT = ex-isotype strain; NT = ex-neotype strain

**Fig. 2:**
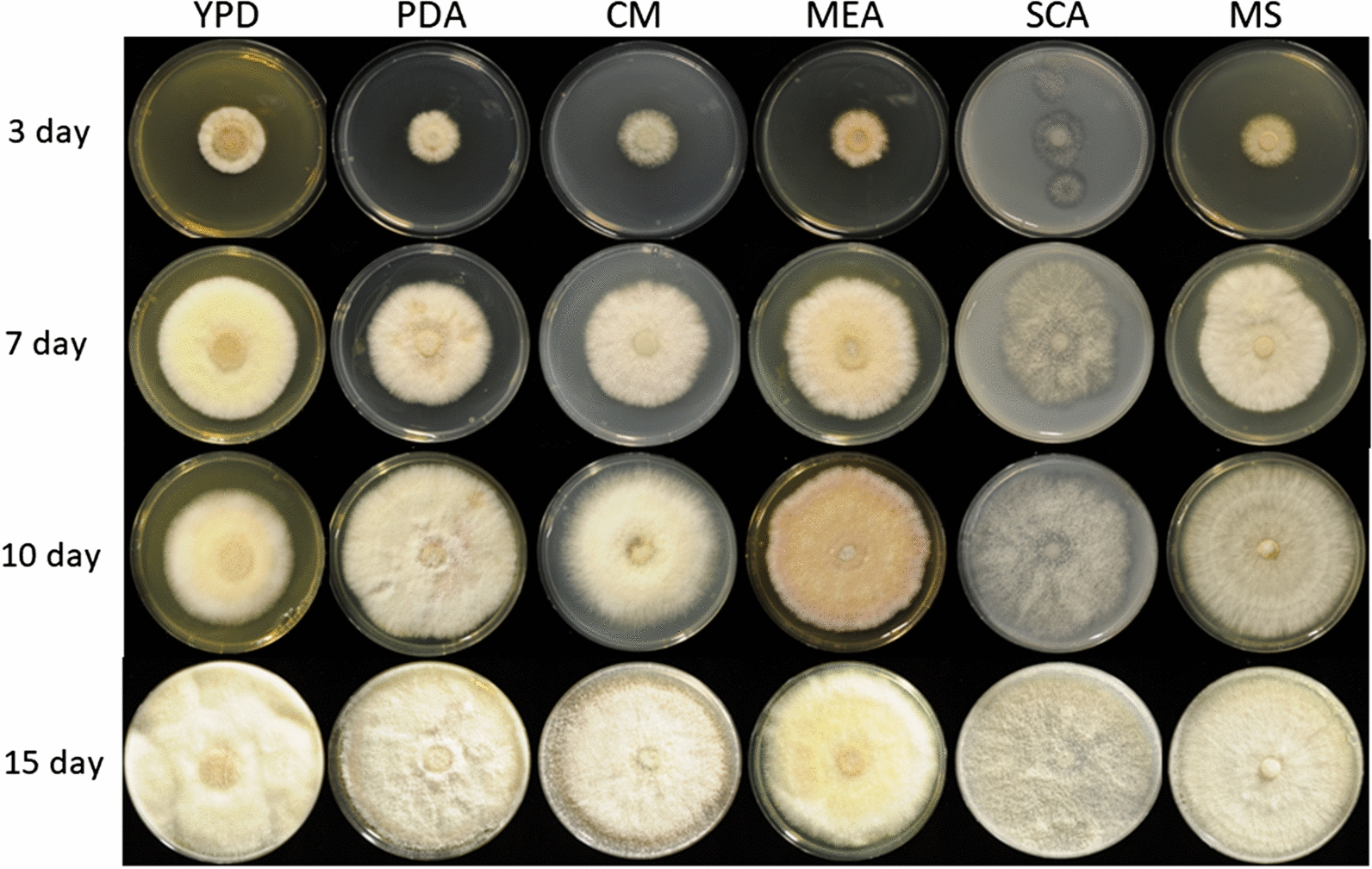
Images of *Fusarium metavorans* FW16.1 (DSM105788) mycelia on six different media over four consecutive days. The selected media were potato dextrose agar (PDA), yeast extract peptone dextrose (YPD), complete medium (CM), malt extract agar (MEA), starch casein agar (SCA) and Mandels’ mineral salts (MS)

We tested the carboxymethylcellulase (CMCase) activity of *F. metavorans* FW16.1 on media containing 1% CMC 3 days after inoculation, revealing a value of 0.055 ± 0.001 U/mg (Additional file 1: Table S1). Genomic DNA was isolated and analyzed by agarose gel electrophoresis (Additional file 1: Fig. S1) and the ITS region was amplified and sequenced (Additional file 1: Supplementary Data). Sequencing identified the isolate as a *F. solani* strain in the FSSC. The strain is preserved at the German Collection of Microorganisms and Cell Cultures (DSMZ) under the identifier DSM105788. The assembled FW16.1 genome was 48.28 Mbp in length, distributed over nine scaffolds with a GC content of 50.83% and an N_50_ scaffold length (weighted median of a contig length needed to cover 50% of the genome) of 6.66 Mbp. The optimal *k-*mer length (subsequences of length *k* contained in genomic sequence) following assembly with SOAPdenovo was *k* = 15 bp, with a pkdepth (peak depth estimated from *k*-mer distribution) of 30. Gene prediction revealed the presence of 15,626 putative open reading frames (ORFs) with an average of 1618.9 bp per gene or 1459.85 bp per coding sequence. The whole genome is available as a biosample from the National Center for Biotechnology Information (NCBI) under the bioproject PRJN413482, accession number JADNRB000000000. Phylogenetic analysis assigned FW16.1 to the FSSC 6 linkage, with highest similarity to *F. metavorans* NRRL 43489 (Fig. 1). Growth on six different media resulted in the formation of pale mycelia (Fig. 2).

### Carbohydrate-active enzyme analysis

**Fig. 3:**
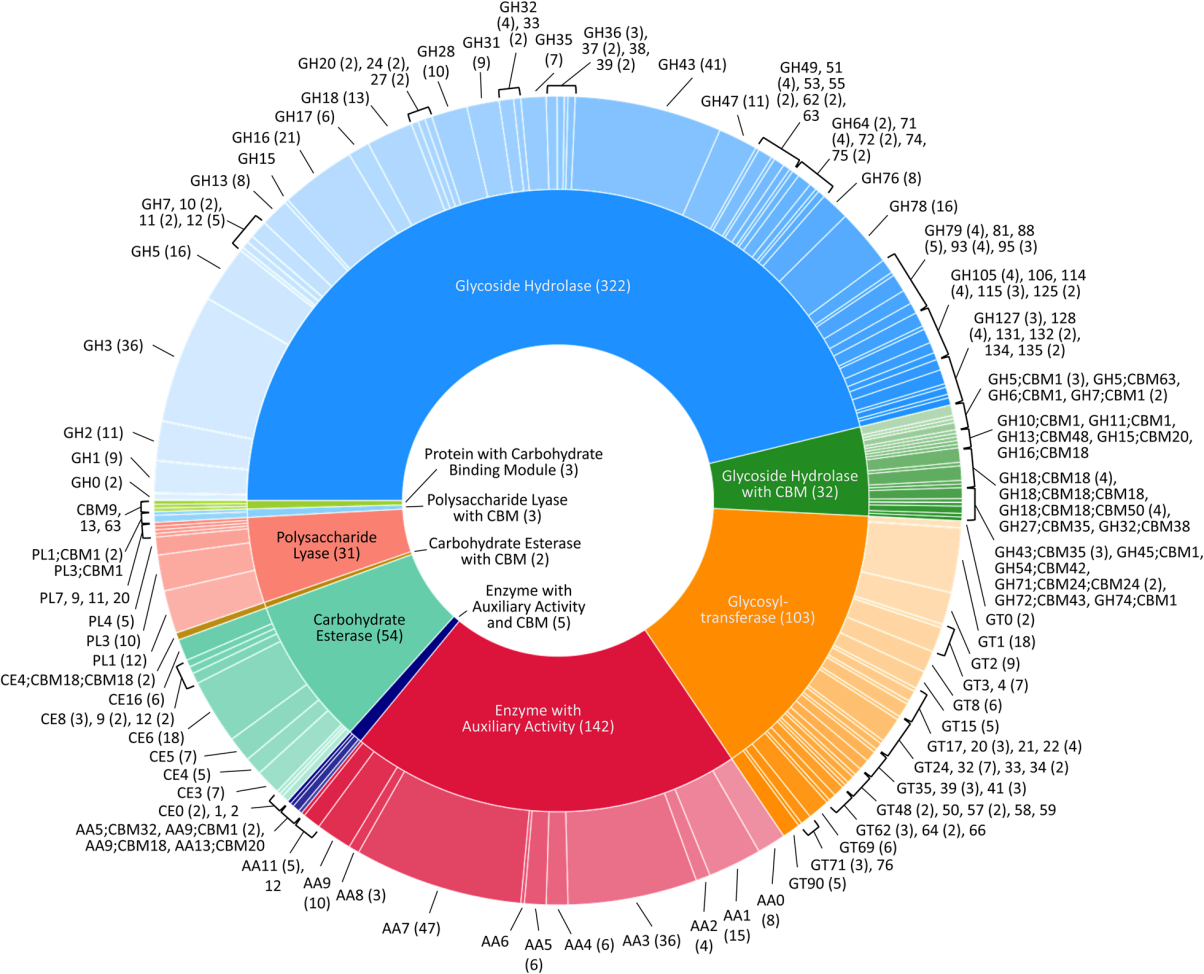
Representation of CAZymes encoded by the *F. metavorans* genome following the analysis of coding regions revealed by de novo sequencing. The inner ring represents the enzyme classes and the outer ring names the families. Numbers in brackets represent the frequency of occurrence, also coded by the size. No number was added if only one enzyme was found

The FW16.1 genomic regions marked as protein coding sequences (CDS) in our de novo assembly were searched for homologs of families (and subfamilies) in the CAZyme database representing enzymes involved in cellulose and sugar metabolism, revealing 694 putative genes (Fig. 3; Table 1). The candidates were assigned to five different carbohydrate-active enzyme (CAZyme) classes, which were divided into their families (Additional file 1: Table S2).

**Table 1:** Identified CAZyme classes based on the *F. metavorans* FW16.1 genome annotation using RAPSearch2 search and HMMER scanning

CAZyme classes	Number of detected genes
Glycoside hydrolases (GH)	322
Glycoside hydrolases (GH) with carbohydrate-binding modules (CBM)	32
Carbohydrate esterases (CE)	54
Carbohydrate esterases (CE) with CBM	2
Polysaccharide lyases (PL)	31
Polysaccharide lyases (PL) with CBM	3
Glycosyl transferases (GT)	103
Glycosyl transferases (GT) with CBM	0
Auxiliary activities (AA)	142
Auxiliary activities (AA) with CBM	5

### Evaluation of enzymatic activity

FW16.1 was cultivated in liquid yeast extract peptone dextrose (YPD) medium, and the enzymatic activity of the supernatant was tested. We observed CMCase activity that increased over the first 2 days, reaching a plateau of ~ 19.5 ± 0.3 U/mg that lasted until day 5. A further increase in activity on days 6 and 7 led to a new plateau at ~ 30 U/mg (Additional file 1: Fig. S2). We then measured enzyme activity induced by cultivation in a range of liquid media containing synthetic and artificial cellulose substrates for 72 h. The activity of the FW16.1 supernatant was 0.039 ± 0.001 U/mg against the crystalline cellulose Avicel PH-101 (Additional file 1: Fig. S3), increasing to 0.07 ± 0.01 U/mg against α-cellulose, and 0.18 ± 0.06 U/mg against hydroxyethylcellulose (HEC). The specific activity against high, medium and low-viscosity forms of CMC, described hereafter as H-CMC, M-CMC and L-CMC for simplicity, was comparable (ranging from 0.07 ± 0.01 to 0.1 ± 0.01 U/mg). We also tested the activity of FW16.1 against agro-residual biomass (sugarcane bagasse and maize leaves) focusing on the properties of the crude secretome. We therefore prepared lyophilized secretome fractions from both biomass types and resuspended them at a 1:1 ratio. The highest polygalacturonase and laminarinase activity was observed after 24 h, whereas the highest CMCase and xylanase activity was observed after 96 h (Additional file 1: Fig. 4A–D). We observed little activity against arabinan, arabinoxylan, galactan, pectin and starch, either due to low enzymatic specificity for these substrates or the low sensitivity of 3,5-dinitrosalicylic acid (DNS) assay.

### Secretome profiling of *F. metavorans* on synthetic substrates and agro-residual biomass

**Fig. 4:**
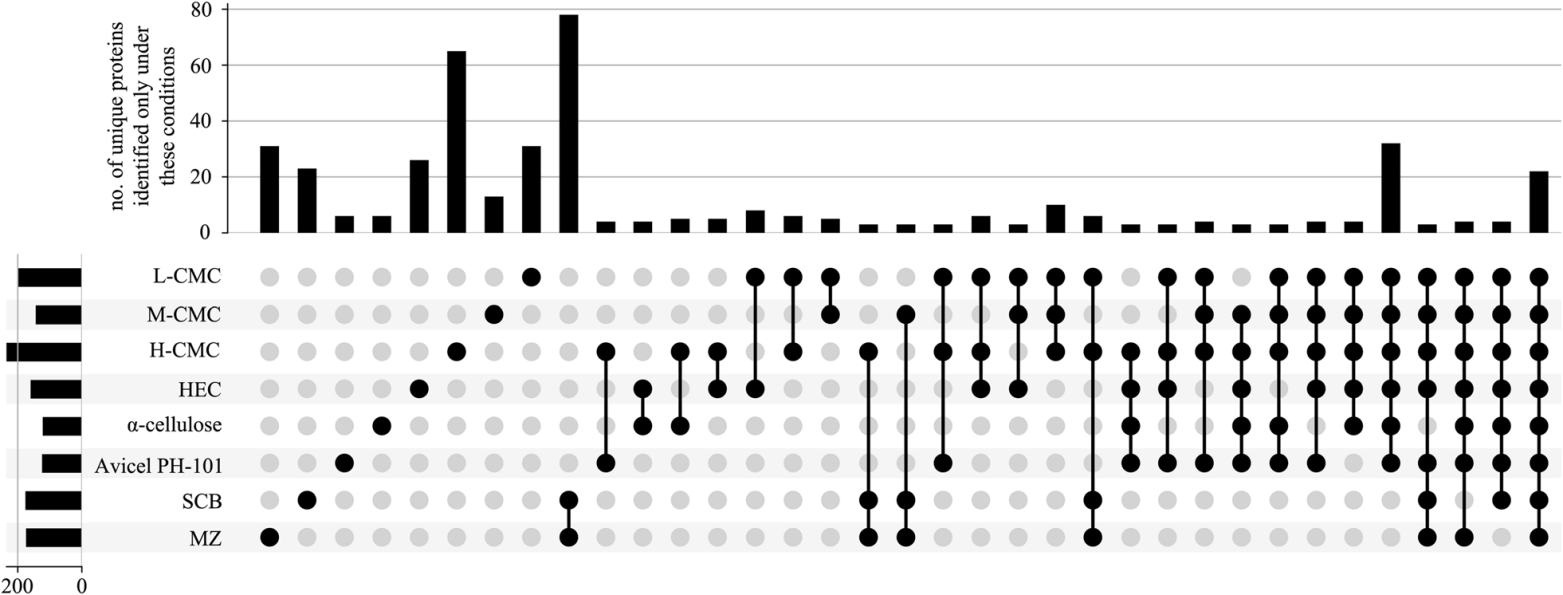
Co-expression of proteins found by mass spectrometric proteomics in different *F. metavorans* secretomes. The connected black dots in the lower part of the figure indicate growth conditions resulting in the expression of a shared set of proteins. The number of proteins found under the specific condition is shown by the size of the black bars. The combinations are sorted to first show the unique proteins, which are specific for certain growth media, and the set of proteins found in all conditions last. From all the possible combinations, only those with more than two co-expressed proteins are shown

Tandem mass spectrometric proteomics was used to analyze the FW16.1 secretome fractions, revealing the presence of 500 proteins (Additional file 1: Table S3). Different numbers of proteins were identified on each substrate, ranging from 122 for α-cellulose to 235 for H-CMC. We identified 124 proteins on Avicel PH-101, 144 on M-CMC, 160 on HEC, 174 on sugarcane bagasse, 176 on maize leaves and 202 on L-CMC. We identified 284 proteins on synthetic or artificial cellulose alone, with the number of unique proteins ranging from six on α-cellulose and Avicel PH-101 to 65 on H-CMC. We identified 13 unique proteins on M-CMC, 26 on HEC, and 31 on L-CMC. We identified 78 proteins solely in the sugarcane bagasse and maize leaf secretome fractions, 23 unique to sugarcane and 31 unique to maize. The largest number of proteins was co-expressed when FW16.1 was grown on the agro-residual biomass, suggesting some of the proteins may be involved in processes not related to energy metabolism (Fig. 4). The second largest number of proteins was co-expressed when FW16.1 was grown on synthetic and artificial cellulose substrates, reflecting the subset of genes required to metabolize these polymers. The third largest number of proteins was common to all conditions, including general sugar conversion and homeostasis genes. Interestingly, the fourth largest group of proteins found on more than one substrate was identified on the CMC media, representing genes specifically required for this artificial substrate. These findings indicate that FW16.1 can fine-tune the expression of relevant genes enabling its survival in different habitats.

**Fig. 5:**
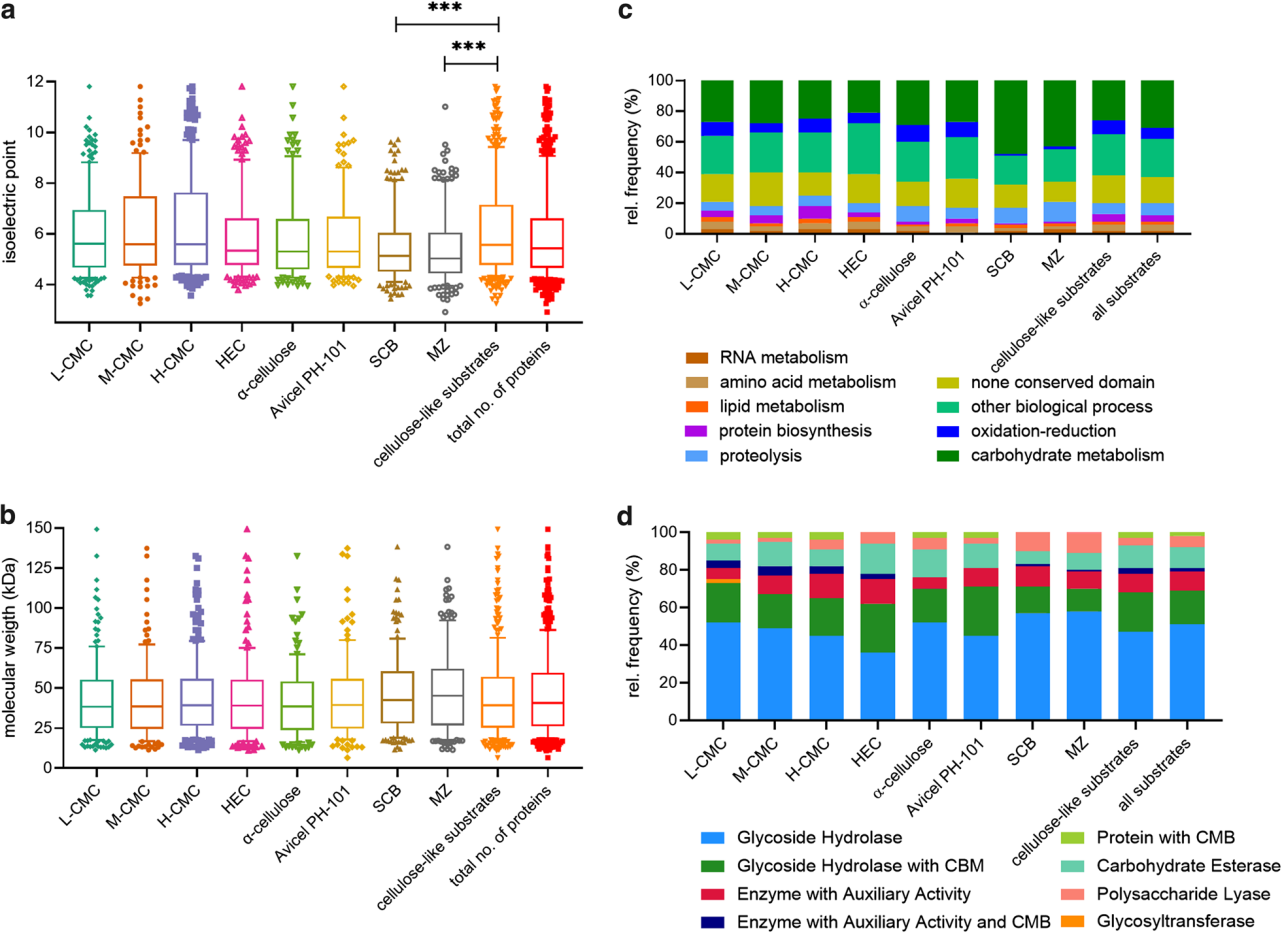
Characterization of proteins found by mass spectrometric proteomics in different *F. metavorans* secretomes. Boxplots show the theoretical isoelectric point (pI) (**a**) and molecular weight (MW) (**b**) of these proteins. The boxplots show the median as a line, the 25% and 75% quantiles as box and the 10% and 90% quantiles as whiskers. There was a highly significant difference between cellulose-like and biomass substrates in pI (***p < 0.0001). Stacked bar plots are classified according to biological activity (**c**) for all proteins, or the distribution of CAZyme classes (**d**)

The theoretical protein distribution was plotted as a function of isoelectric point (pI) (Fig. 5a) and molecular weight (MW) (Fig. 5b), revealing that 90% of the secretome proteins fell within the MW range 6.5–263.4 kDa (median = 40.8 kDa) and the pI range 2.9–11.8 (median = 5.4). On the six synthetic and artificial cellulose substrates, the median size of the secretome was 38.5–39.5 kDa, but this shifted to 42.5 and 45.1 kDa on the two biomass substrates. Similarly, the median pI was 5.3–5.6 on the synthetic and artificial cellulose substrates, but shifted to 5.0 and 5.1 on maize and sugarcane bagasse, respectively. This effect appears small, but the pI has a logarithmic scale and more than 135 proteins were analyzed for both parameters, resulting in significant deviations (*p* < 0.0001) based on an unpaired *t*-test assuming Gaussian distribution (Fig. 5).

**Fig. 6:**
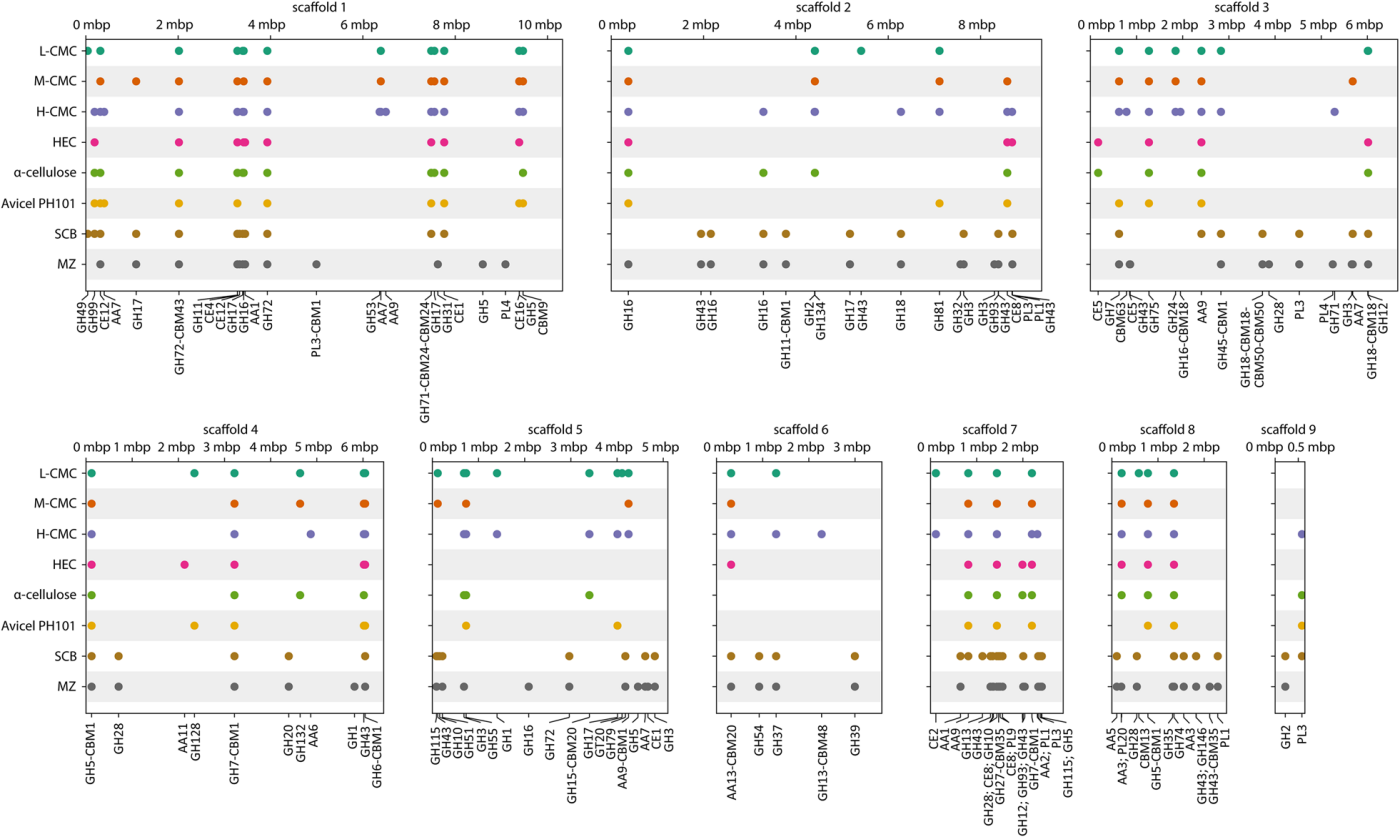
Mapping of 135 CAZymes found by mass spectrometric proteomics in different *F. metavorans* secretomes. *F. metavorans* FW16.1 was grown on eight different substrates (*y*-axis) differing in complexity. The CAZymes identified by MS were mapped back to protein coding regions (CDS) in our de novo genome assembly, which consists of nine scaffolds. A circle indicates the genomic location of a CDS with a positive proteomics mapping. The circle color corresponds to the growth substrate. The CDS is annotated with its CAZyme family name on the *x*-axis. For example, the same CDS on scaffold 2, annotated as GH16, is expressed/secreted in all growth substrates except sugarcane bagasse, and is located at position 389,635–390,517 bp (exact position not shown for clarity)

To gain insight into the metabolic diversity of the secretome on each substrate, the identified proteins were classified according to biological function (Fig. 5c) based on the sequences listed in Additional file 1: Table S3. Several molecular functions were identified, including carbohydrate, lipid, RNA and amino acid metabolism, protein synthesis, redox processes, proteolysis, and proteins with unknown functions. The proteins identified on the synthetic and artificial cellulose substrates were distributed similarly according their molecular functions, whereas the relative frequency of proteins related to carbohydrate metabolism was higher on the biomass substrates. The substrate-dependent profiles of the 135 CAZymes are shown in Fig. 5d; a complete list of identified CAZymes with associated modules is provided in Table 2. Predictions based on putative molecular functions for all proteins are summarized in Additional file 1: Table S3. The 135 CAZymes were assigned to five different classes (Table 2): 93 glycoside hydrolases (GHs), 17 auxiliary activities (AAs), 12 carbohydrate esterases (CEs), 12 polysaccharide lyases (PLs), and one glycosyltransferase (GT), as well as three non-catalytic carbohydrate-binding modules (CBMs). The distribution over the scaffolds is presented in Fig. 6.

**Table 2:**
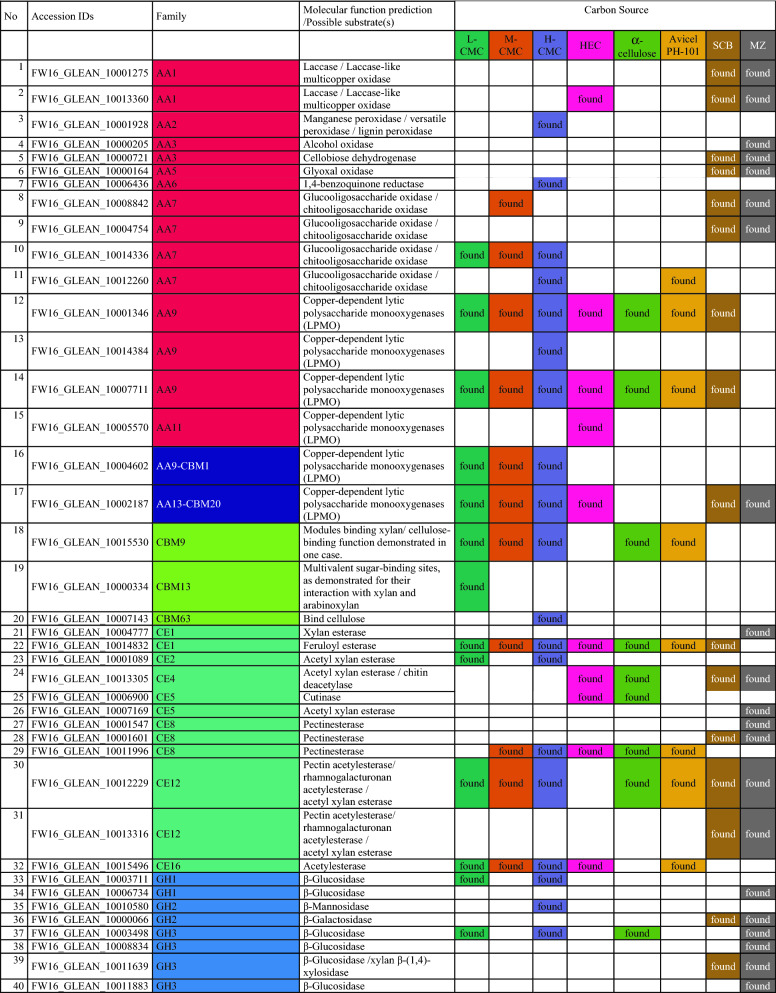

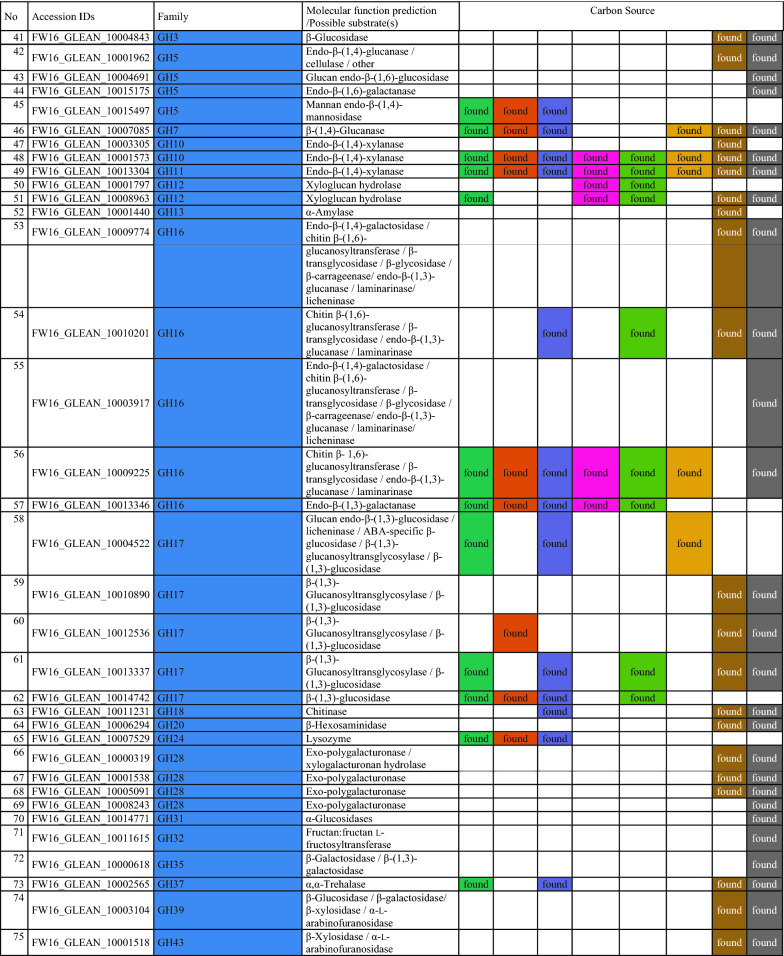

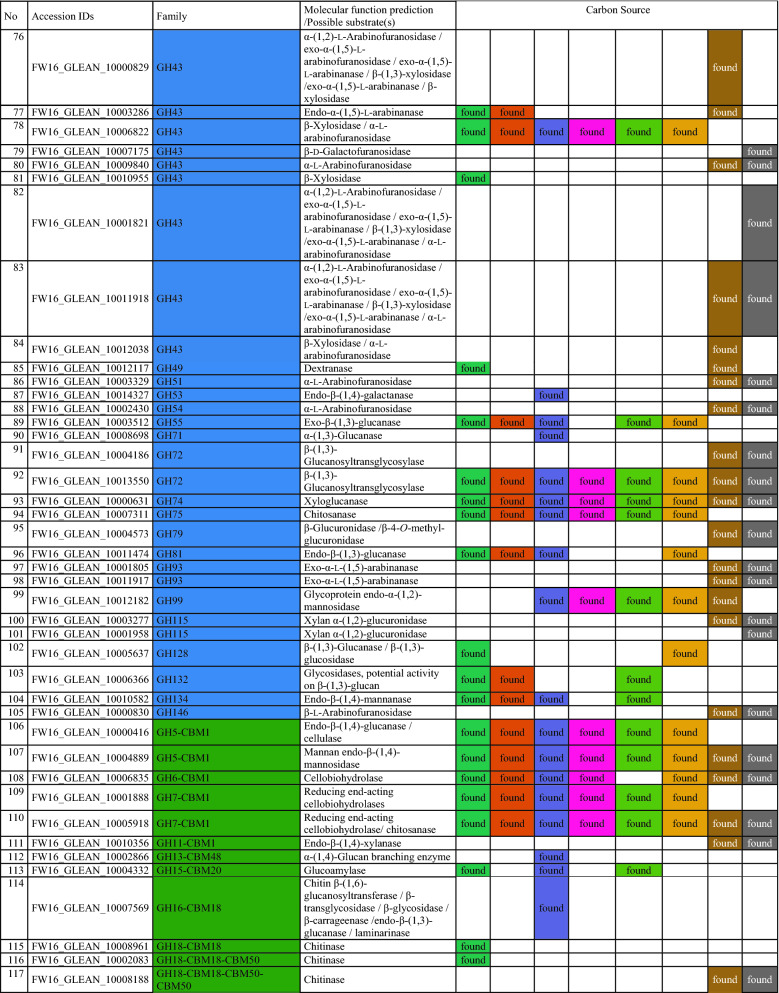

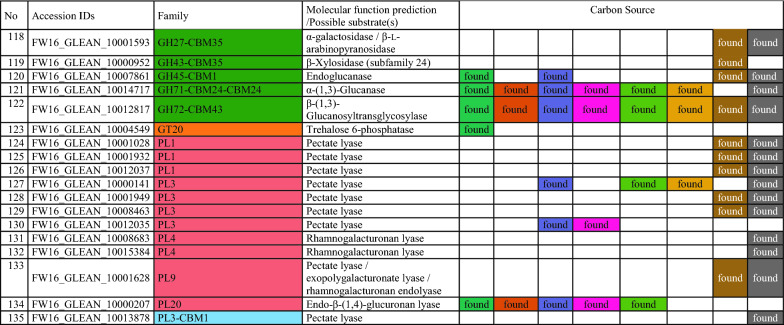
CAZymes identified in the secretome of *F. metavorans* DSM105788 after liquid fermentation on six different synthetic/artificial cellulose substrates as well as two different agro-residual biomasses. The color coding is identical to Fig. 4

The most abundant CAZyme class was the GHs (36–58%, or 62–73% when including GHs with CBMs), confirming their general role in cellulose degradation. The lowest proportion of GHs (36%) was identified on the substrate HEC, which also featured the highest proportion of CEs (16%) compared to the median 11%. Another unusual profile was the overrepresentation of PLs on natural substrates, with 7–8 enzymes (10%) compared to 1–3 (2–6%) on the synthetic and artificial cellulose substrates. The natural substrates also featured more GHs (57–58%), but fewer GH-CBMs (12–14%) than the synthetic and artificial cellulose substrates, which featured 47% GHs and 22% GH-CBMs on average.

Some CAZymes were produced on all substrates, whereas others were more specific. When the “core” GH family proteins (produced on at least five of the six synthetic and artificial celluloses) were evaluated, few differences were found: two GH5, one GH6, two GH7 (all five with CBM1) one GH10, one GH11, one GH16, one GH43, one GH55, one GH71-CBM24-CBM24, one GH72-CBM43, one GH74 and one GH75. Predicted cellulase activities were confirmed in several cases: endo-β-(1,4)-glucanase activity for FW16_GLEAN_10000416 (GH5-CBM1), cellobiohydrolase activity for FW16_GLEAN_10006835 (GH6-CBM1), reducing-end cellobiohydrolase activity for FW16_GLEAN_10001888 and FW16_GLEAN_10005918 (both GH7-CBM1), and potentially xyloglucanase activity for FW16_GLEAN_10000631 (GH74). Remarkably, no GH with predicted β-glucosidase activity was found on the cellulose and cellulose-like substrates, whereas FW16_GLEAN_10003711 (GH1) and FW16_GLEAN_10003498 (GH3) were found on four of the six substrates. Furthermore, enzymes with predicted β-(1,3)-glucanase activity representing GH families 16, 17, 55, 72, 81, 128 and 132 were found mostly on the artificial cellulose substrates and especially on the CMCs, suggesting the broader substrate specificity of those enzymes or a weak catalytic promiscuity. More diverse cellulose-degrading enzymes were identified on the biomass substrates: FW16_GLEAN_10011639 and FW16_GLEAN_10004843 (GH3), FW16_GLEAN_10001962 (GH5), FW16_GLEAN_10006835 (GH6-CBM1) and FW16_GLEAN_10005918 (GH7-CBM1), the latter also found on the artificial celluloses.

As suspected, the synthetic and artificial cellulose substrates contained fewer GH family proteins predicted to degrade hemicellulose or pectin compared to the biomass: FW16_GLEAN_10000066 (GH2), FW16_GLEAN_10001573 (GH10) and FW16_GLEAN_10013304 (GH11), FW16_GLEAN_10003286, FW16_GLEAN_10006822 and FW16_GLEAN_10010955 (GH43). However, a similar distribution was found under both conditions for β-galactosidase (FW16_GLEAN_10000066, GH2) a potential xylan β-(1,4)-xylosidase (FW16_GLEAN_10011639, GH3), β-(1,3)-glucosidase (FW16_GLEAN_10010890, GH17), exo-polygalacturonase (FW16_GLEAN_10001538 and FW16_GLEAN_10005091, both GH28), and potential disaccharide hydrolases such as FW16_GLEAN_10003104 (GH39), FW16_GLEAN_10001518, FW16_GLEAN_10009840 and FW16_GLEAN_10011918 (all GH43) and two exo-α-l-(1,5)-arabinanases (FW16_GLEAN_10001805 and FW16_GLEAN_10011917, both GH93). GH proteins identified solely on sugarcane bagasse were related to xylan, amylase and dextran degradation (GH10, GH13, and four of the 11 GH43 and GH49 proteins). In contrast, those identified solely on maize leaves were primarily related to disaccharide hydrolysis, including FW16_GLEAN_10006734 (GH1), FW16_GLEAN_10003498 and FW16_GLEAN_10008834 (both GH3, β-glucosidase), FW16_GLEAN_10000618 (GH35, β-galactosidase or β-(1,3)-galactosidase) and two of the 11 GH43 proteins (FW16_GLEAN_10007175 β-d-galactofuranosidase, and FW16_GLEAN_10001821, predicted arabinanase or xylosidase).

A clearer picture emerged for the AAs. The synthetic and artificial cellulose substrates mainly featured AA9 proteins with lytic cellulose monooxygenase activity, whereas the biomass substrates showed a greater diversity of AA families. Some were predicted to modify lignin, such as the laccases FW16_GLEAN_10001275 and FW16_GLEAN_10013360 (both AA1), the alcohol oxidase FW16_GLEAN_10000205 (AA3), the cellobiose dehydrogenase FW16_GLEAN_10000721 (AA3), and glyoxal oxidase FW16_GLEAN_10000164 (AA5). Interestingly, no AA9 proteins were found on maize leaves, but two of the four identified AA9 proteins were found on sugarcane bagasse.

Among the 12 identified PLs, six were found on sugarcane bagasse and 10 were found on maize, highlighting their role in pectin degradation. Only 1–3 PLs were found on the synthetic and artificial cellulose substrates, with FW16_GLEAN_10000207 (PL20, predicted endo-β-(1,4)-glucuronan lyase) present on five of the six cellulase substrates but not on the biomass substrates.

We identified 4–5 CEs restricted to the synthetic and artificial cellulose substrates, five produced on sugarcane, and seven produced on maize. In the latter case, roles in hemicellulose and pectin degradation are likely, such as FW16_GLEAN_10004777 (CE1) and FW16_GLEAN_10007169 (CE5), both with predicted (acetyl)xylan esterase activity, FW16_GLEAN_10001547 and FW16_GLEAN_10001601 (both CE8), FW16_GLEAN_10012229 and FW16_GLEAN_10013316 (both CE12), all four with predicted pectinase activity. Sugarcane bagasse contained both CE12 enzymes also found on maize leaves, as well as one common CE8 and CE4 protein, and the CE1 protein FW16_GLEAN_10014832 with predicted feruloyl esterase activity. CEs solely present on the synthetic and artificial cellulose substrates included FW16_GLEAN_10001089 (CE2, acetylxylan esterase), FW16_GLEAN_10006900 (CE5, pectin esterase), FW16_GLEAN_10011996 (CE8, cutinase) and FW16_GLEAN_10015496 (CE16, acetyl esterase). We identified only one GT protein (FW16_GLEAN_10004549, GT20) and this was found on the L-CMC substrate.

Finally, we identified proteins representing three CMB families present solely on the synthetic and artificial cellulose substrates: FW16_GLEAN_10015530 (CBM9), FW16_GLEAN_10000334 (CBM13) and FW16_GLEAN_10007143 (CBM63). Interestingly, CBM9 and CBM63 are predicted to bind cellulose but CBM13 is not. Another eight CBM families were represented in the modular proteins described above, combined with GH, AA or PL domains, and these were distributed similarly between the synthetic cellulose and biomass substrates. CBM1 was the most abundant module (nine identified in total), and was associated with GH, AA and PL proteins, whereas the other CBMs were found only 1–3 times each.

### Conversion of biomass with the in-house *F. metavorans* cocktail

The overall enzymatic activity of the crude secretome preparations was low. We therefore lyophilized the enzymes secreted on both biomass substrates, resuspended them in 50 mM citrate buffer (pH 4.8), and combined them at a 1:1 ratio with a final protein concentration of 312 ± 2.7 µg/mL. We then prepared saturation curves (Additional file 1: Table S5).

**Fig. 7:**
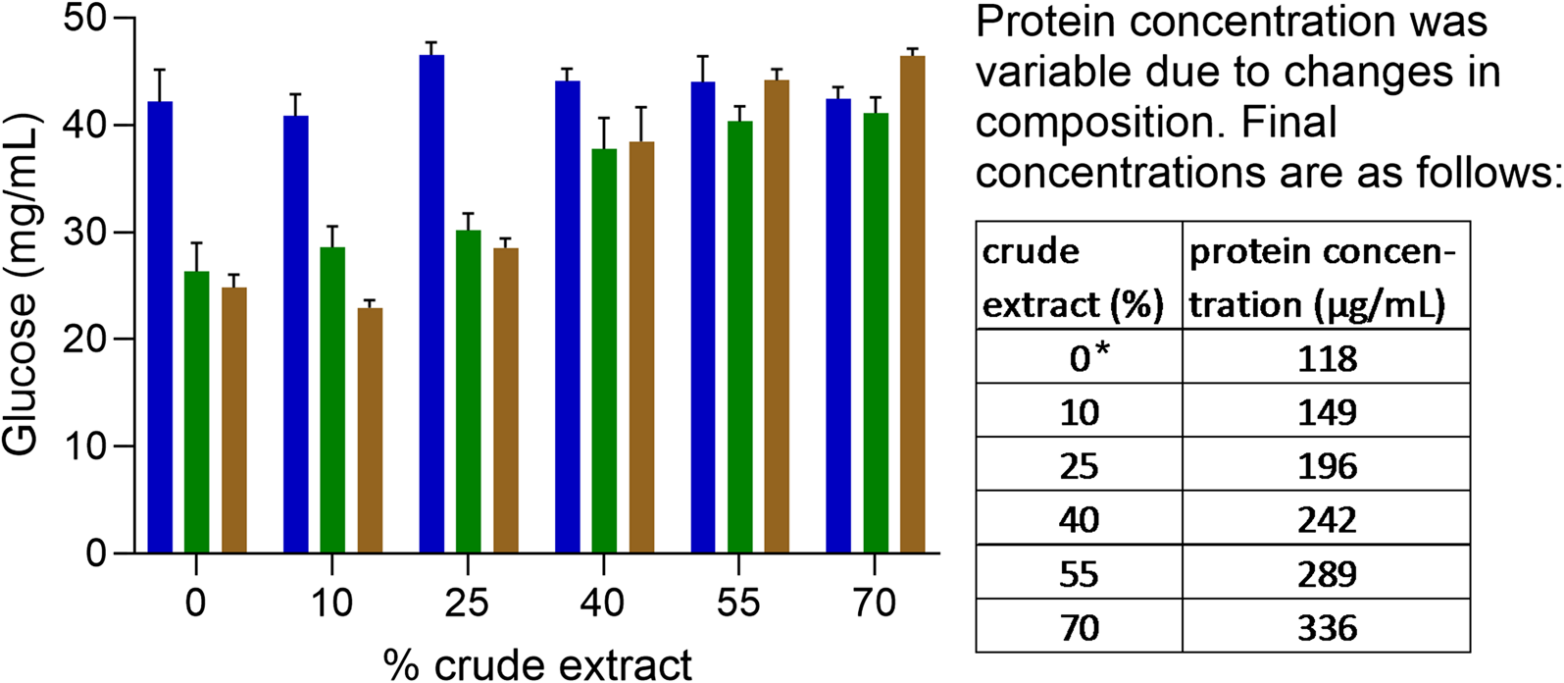
Glucose release by the enzyme mix on steam-exploded sugarcane bagasse (XSCB), untreated (in nature) sugarcane bagasse (NSCB) and maize leaves (MZ). The enzyme mix consisted of the *F.* *metavorans* in-house cocktail supplemented with Accellerase 1500 and was applied in increasing concentrations. Protein concentrations are shown in the table to the right. All mixtures contain a small amount of Accellerase 1500, which explains the protein content in the sample without crude extract (0%). XSCB is shown in blue, NSCB in brown and MZ in green

Hydrolysis assays were evaluated against three different substrates: steam-exploded sugarcane bagasse (XSCB), untreated (in nature) sugarcane bagasse (NSCB) and untreated maize leaves (MZ), each present at a concentration of 5% (w/v) for 24 h. Control assays without in-house enzymes (A1) were also prepared. All assays were supplemented with the commercial Accellerase 1500 enzyme mixture containing exoglucanase, endoglucanase, hemi-cellulase and β-glucosidase at a concentration of 5 FPU/mL (filter paper unit). Under control conditions (A1), XSCB was converted to glucose 1.6-fold more efficiently than the other substrates (Fig. 7). To test the activity of the secretome preparation, we supplemented the assay with the *F. metavorans* in-house cocktail at concentrations ranging from 10% (v/v) in assay A2 to 70% (v/v) in assay A6 (Additional file 1: Table S5).

Figure 7 shows the glucose profile following biomass hydrolysis in all assays (A1–A6). XSCB was easily converted to glucose by the commercial Accellerase 1500 enzyme mix, but the in-house cocktail did not facilitate further saccharification. In contrast, the in-house cocktail enhanced the release of sugars from the NSCB and MZ substrates starting at concentrations of 25% (v/v), corresponding to 196 µg/mL. When the concentration of the in-house cocktail reached 55% (v/v), corresponding to 0.289 µg/mL, the efficiency of saccharification became equivalent to that of the pre-treated (XSCB) substrate. An in-house enzyme cocktail with a protein load of 35–36 mg/g biomass therefore facilitated synergistic depolymerization without pre-treatment, achieving a statistically significant improvement in glucose yields (*p* < 0.05, 95% confidence).

## Discussion

We set out to characterize an active fungal isolate by identifying enzymes that facilitate the utilization of plant biomass, particularly those involved in cellulose degradation. We compared the enzymes induced by different synthetic cellulose substrates, and analyzed secretome components on two different types of agro-residual biomass representing the C4 crops sugarcane and maize [25, 44]. We also assigned the fungal isolate to the correct FSSC linkage. To the best of our knowledge, this is the first comparative analysis of the *F. metavorans* as a strain of the FSSC secretome on different substrates.

Analysis of the 62 proteins produced on all six artificial cellulose substrates revealed only 16 CAZymes, five of which were predicted to degrade cellulose. The enzymes were assigned to CAZy families GH5, GH7 and AA9. The corresponding genes were distributed over four different scaffolds, but there was no clear evidence for clusters of colocalized or coregulated genes. The hydrolytic degradation of cellulose by fungi involves at least three steps: (1) internal cellulose bonds are cleaved by endo-β-(1,4)-glucanases (GH5) [45–47] to create shorter polymers; (2) these are digested by exo-β-(1,4)-glucanases and/or cellobiohydrolases (GH7 and GH6) ultimately to produce cellobiose, which is (3) finally converted into two glucose molecules by β-glucosidases (mainly GH1 or GH3, and some others such as GH39) [48, 49]. At least the first two steps were recapitulated in the *F. metavorans* FW16.1 secretome fractions. For the first step, one predicted GH5 protein with cellulase activity (FW16_GLEAN_10000416) was found on all cellulose substrates, whereas another (FW16_GLEAN_10001962) was found on the biomass substrates. For the second step, one GH7 with a CBM1 domain (FW16_GLEAN_10005918, predicted cellobiohydrolase) was found on all substrates, another (FW16_GLEAN_10001888) was found on the artificial cellulose substrates, and a third without a CBM (FW16_GLEAN_10007085) was found on six of the eight substrates. Some proteins with predicted β-glucosidase activity (GH1, GH3 maybe GH39) were also found, but none of them were present on all substrates.

We also identified an AA9 lytic polysaccharide monooxygenase (LPMO) that can oxidize the C-1 or C-4 (and perhaps C-6) positions of the glycosidic bond in cellulose and disrupt its structure, as shown for the fungi *Podospora anserina* and *Neurospora crassa* [50, 51]. An interesting combination of AA9 and PL20 was observed, where glycosidic bonds of glucuronic acid-containing cello-oligosaccharides produced by AA9 proteins may be cleaved at the C4-position by the PL20 family via β-elimination to produce a reducing end [52]. This mechanism could also be involved in cellulose degradation, as already postulated for the fungus *Humicola insolens* [53]. A clear difference in cellulose degradation was identified between the biomass substrates, with more GHs found on maize leaves contrasting with more AAs catalyzing oxidative cellulose degradation on sugarcane bagasse, the latter indicating a more complex cellulose architecture [54]. The GH74 family, with predicted xyloglucanase activity, was also found on all substrates, and may therefore contribute to cellulose degradation. This is supported by the identification of a GH74 xyloglucanase from the bacterium *Cellvibrio japonicas* with a strong preference for xyloglucans but some activity (24–165-fold lower) against artificial substrates such as CMC and HEC [55]. Another protein (AA13) fused to the starch-binding module CBM20 [56] was found on four of the six synthetic cellulose substrates, perhaps indicating promiscuous activity against artificial celluloses. However, no cellulase activity was previously reported for AA13 enzymes isolated from the fungi *Neurospora crassa* and *Aspergillus nidulans* [57, 58].

The distribution of the CAZymes on the two biomass substrates was more complex, mirroring the complexity the substrates, including the presence of hemicellulose, pectin and lignin. The secretome fractions thus included a lignocellulolytic enzyme cocktail with the ability to degrade all cell wall polymers and stored starch granules, including cleavage by lyases and oxidation.

The degradation of hemicellulose requires enzymes specific for β-(1,4)-linked xyloses or xyloglucan and arabinoxylan acetylated at the C2 and/or C3 positions as well as β-(1,3), β-(1,4) and β-(1,6) glucan branches [59] that connect pectin to cellulose [17, 60]. These include endo-β-(1,4)-xylanases (GH10, GH11), α-l-arabinofuranosidases and exo-α-l-(1,5)-arabinanases (GH3, GH43, GH51, GH54, GH62 and GH93), β-xylosidases (GH43 and GH3), acetylxylan esterases (CE1–CE7), and ferulic acid esterases (CE1) and acetylesterases (CE16) [48]. We found three GH10 and GH11 proteins on maize and four on sugarcane. We also found GH27-CBM35, GH31 and GH35 proteins (the latter two restricted to maize) two GH93 proteins and one GH115 protein, all probably responsible for hemicellulose or rhamnogalacturonan I (pectin) degradation [61]. The GH43 family, which converts xylo-oligosaccharides containing arabinose and galactose to monomers, was also found on both substrates. We identified 11 GH43 enzymes in total, three exclusively on sugarcane and two on maize. Previous secretome analysis of *Trichoderma reesei* and *Aspergillus niger* on sugarcane bagasse [62], *A. nidulans* on sorghum [60], *Myceliophthora thermophila* on sugarcane bagasse [63] and *N. haematococca* on maize bran [64] identified one, five, eight, four and four GH43 proteins, respectively. We also identified multiple xylan esterases (CE1, CE4 and CE5): three on maize and one on sugarcane, similar to the *T. reesei* secretome on sugarcane bagasse which features two CE5 proteins [62]. In contrast, no CEs were found in the secretome fractions of *N. haematococca* on maize bran [64].

A combination of GHs, CEs and PLs was needed to break down pectin in our biomass substrates [19]. The GHs we identified represented families GH28 (four in total, one only found on sugarcane), GH43 and GH79 [19], perhaps also including GH35, GH51 and GH93 (which can digest rhamnogalacturonan I) [65]. We identified three CE8 proteins (two found only on sugarcane) and two CE12 proteins (required to remove branches from non-sugar components containing methyl and acetyl groups). Finally, we identified six PLs from families PL1, PL3 and PL9 on sugarcane, and 10 PLs from families PL1, PL3, PL4 and PL9 on maize. These are necessary for the efficient utilization of homogalacturonan and rhamnogalacturonan. In contrast, no pectin-digesting GHs, CEs and PLs were identified in the secretome of *N. haematococca* on maize bran, whereas the *A. niger* BRFM442 secretome contained six GH28, two CE8 and one PL proteins on the same substrate [64].

The AA superfamily of lignolytic enzymes and monooxygenases [66] was also found in the secretome induced on our maize and sugarcane substrates. Laccases (AA1) oxidize a wide range of aromatic compounds including polyphenols, methoxy-substituted monophenols and aromatic amines [67] and these were found on both substrates. When other *F. solani* strains were cultured on substrates such as oak combined with millet and wheat bran or corn, wheat, rye and oat, the secretome fractions contained laccases as well as manganese-dependent peroxidases (MnP) and lignin peroxidases (LiP), both of which represent family AA2 [68, 69]. We did not find any AA2 proteins, perhaps because we investigated only a limited set of time points, thus providing an incomplete picture of oxidative lignin degradation. However, we identified AA3 flavoenzymes on both substrates, and this family includes glucose oxidases and aryl alcohol oxidases that act on the anomeric carbon of β-d-glucose and alcohols using molecular oxygen as an electron acceptor, releasing hydrogen peroxide [70]. It is interesting to note that feruloyl and *p*-coumaroyl esterases were not found on the maize substrate, whereas one CE1 protein with that predicted function was found on sugarcane and all the cellulose substrates. These esterases normally remove the crosslinks between polysaccharides and lignin to increase enzymatic access to the cell wall [62, 63]. The analysis of an *A. nidulans* strain on sorghum stover revealed only two esterases in the secretome [54].

Several of the enzymes discussed above overcome the inaccessibility of insoluble substrates by using one or more non-catalytic CBMs [71]. Examples include the GH5, GH7, GH11, GH45 and PL3 families, which are frequently associated with CBM1 (which typically binds cellulose) [72]. Three of the five GH18 family members we identified were associated with the chitin-binding modules CBM18 and CBM50. Similarly, the *T. reesei* genome encodes at least 18 GH18 proteins, four with additional CBMs [73]. A glucoamylase (GH15) associated with the starch-binding module CBM20 was found in *Penicillium echinulatum* [74], and we identified α-(1,4)-glucan branching enzymes (GH13) associated with the glycogen-binding module CBM48, which has been found in several other species [75]. We also identified an α-(1,3)-glucanase (GH71) associated with the starch-binding module CBM24, and an α-galactosidase (GH27) associated with CBM35, which was shown to bind β-galactans in *Phanerochaete chrysosporium* [66].

Our comparative approach revealed 500 secretome proteins, including 93 GH proteins representing 40 different families. A similar range was reported *F. solani* ATCC MYA 4552 cultivated on a mixture of oak, millet and wheat, where 398 proteins were identified, including 48 GH proteins representing 28 families [69]. We compared the secretome proteins of our *F. metavorans* FW16.1 isolate on natural substrates with nine other fungal secretome fractions [60, 62, 64, 76–78]. In most cases, our isolate produced a larger number of secreted CAZymes, with only *A. nidulans* strain A78 grown on sorghum stover and *A. niger* BRFM442 grown on maize bran producing more (Table 3). The cultivation of *N. haematococca* on maize bran produced four GH43 proteins but no members of the families GH5, GH6, GH7 or AA9, arguing that maize bran induces the secretion of hemicellulases [64]. We found that *Fusarium* sp. of the FSSC uses their diverse arsenal of depolymerizing and accessory enzymes as destruents to break down complex substrates, supported by their adaptation to different environments, their metabolic plasticity, and their ability to degrade different lignocellulose materials [69, 79], as well as other compounds such as the pesticide dichlorodiphenyltrichloroethane [80].

**Table 3:** Comparison of CAZymes identified by mass spectrometric proteomics in six different studies encompassing seven different fungi, five different types of biomass, and six different synthetic and artificial cellulose substrates

Strain	Substrates	Fermentation	GHs	CEs	AAs	PLs	GTs	Source
*Trichoderma reesei* RUT-C30	Exploded sugarcane bagasse	Solid-state	15	0	1	0	0	[62]^a^
*T. reesei* RUT-C30	Sugarcane culm	Solid-state	19	0	1	0	0	[62]^a^
*Aspergillus niger* N402	Exploded sugarcane bagasse	Solid-state	35	10	2	1	0	[62]^a^
*A. niger* N402	Sugarcane culm	Solid-state	34	5	1	1	0	[62]^a^
*Fusarium metavorans*	In nature sugarcane bagasse	Liquid fermentation	49	8	9	7	0	This work
*F. metavorans*	Maize leaves	Liquid fermentation	58	11	8	8	0	This work
*F. metavorans*	L-CMC	Liquid fermentation	39	6	5	1	1	This work
*F. metavorans*	M-CMC	Liquid fermentation	26	5	6	1	0	This work
*F. metavorans*	H-CMC	Liquid fermentation	40	7	9	3	0	This work
*F. metavorans*	HEC	Liquid fermentation	19	6	5	2	0	This work
*F. metavorans*	α-Cellulose	Liquid fermentation	27	6	2	2	0	This work
*F. metavorans*	Avicel PH-101	Liquid fermentation	21	4	3	1	0	This work
*Nectria haematococca*	Maize bran	Liquid fermentation	30	0	3	2	0	[64]
*A. niger* BRFM 442	Maize bran	Liquid fermentation	77	5	3	1	0	[64]
*Trichoderma asperellum* S4F8	In nature sugarcane bagasse	Solid-state	61	3	0	0	1	[77]
*T. reesei* RUT-C30	In nature sugarcane bagasse	Solid-state	37	3	2	0	1	[77]
*Trichoderma harzianum* P49P11	Exploded sugarcane bagasse	Liquid fermentation	19	0	1	0	0	[78]
*T. harzianum* IOC 3845	In nature sugarcane bagasse	Liquid fermentation	57	3	1	0	0	[76]
*Aspergillus nidulans* A78	Sorghum stover	Solid-state	79	5	4	8	0	[60]^a^

^a^Time course analysis

The *F. metavorans* in-house enzymatic cocktail proved a suitable alternative to the chemical pre-treatment of agro-residual lignocellulosic biomass, clearly allowing the debranching of polymers surrounding the cellulose fibers and releasing reducing sugars (Fig. 7). Pre-treatment methods are often needed for recalcitrant biomass such as hemicellulose, lignin and crystalline cellulose, to open up the fibers and improve accessibility to the polymers [44, 81]. Accordingly, the *F. metavorans* in-house cocktail did not enhance the production of sugars from sugarcane biomass subjected to steam explosion, because pre-treatment had already rendered the polymers fully accessible to the Accellerase 15,000 cocktail. However the in-house cocktail had a strong impact on the saccharification of untreated maize and sugarcane biomass, with additional advantages over chemical pre-treatment such as selectivity, mass efficiency (the released carbohydrates are retained and utilized), and the avoidance of inhibitory by-products. Furthermore, no toxic compounds are dispersed into the environment, avoiding the need to recycle or remove them. The *F. metavorans* enzyme cocktail therefore provides a sustainable, low-energy process to enhance the efficiency of enzymatic saccharification [82–84].

## Conclusion

The CAZymes identified in this study can be used to enhance the enzymatic saccharification of agro-residual biomass. Our workflow involved strain isolation, genome sequencing, CAZyme analysis and secretome analysis by mass spectrometric proteomics, revealing 135 relevant enzymes. The *F.* *metavorans* in-house cocktail was used to increase the amount of glucose generated from maize leaves and untreated sugarcane bagasse by selective pre-treatment, improving the turnover of the hemicellulose fraction without carbohydrate loss or the formation of inhibitory by-products.

## Materials and methods

### Fungus isolation and growth conditions

The fungal isolate *F. metavorans* FW16.1, was obtained from mangrove wood [43] in Vietnam (longitude 10°36′015′′N, latitude 106°56′045′′E) and prepared as a conidial suspension. Mycelium pieces (5 mm diameter) on potato dextrose agar (PDA) were transferred to a fresh PDA plate and grown in the dark for 5–7 days at 28 °C. The conidia were scraped with a Drigalski cell spreader in sterile water and centrifuged at 2693 × *g* for 15 min at 4 °C. The pellet was washed in sterile water, filtered through a 40-µm mesh sieve and centrifuged as above. The pellet was resuspended in sterile water, aliquoted and stored at – 70 °C. To investigate mycelial growth and color formation, fungal growth was assessed on PDA, YPD [85], complete medium (CM) [86], malt extract agar (MEA) [87], starch casein agar (SCA) [88] and Mandels’ salt medium (MS) [89] for 15 days (Fig. 2).

### Phylogenetic analysis and de novo sequencing

Submerged cultures of *F. metavorans* FW16.1 were established in potato dextrose broth (PDB) and incubated at 28 °C, shaking at 150 rpm. DNA was isolated according to the CTAB method [90, 91] and purity and quality were confirmed by gel electrophoresis and spectrophotometry. We used 11 μg of pure high-molecular-weight genomic DNA (gDNA) for the de novo preparation of 270-bp short HiSeq and PACBIO RSII 20 K sequencing libraries. Following gene prediction, ORFs were identified and annotated according to Gene Ontology (GO), Kyoto Encyclopedia of Genes and Genomes (KEGG) and Clusters of Orthologous Groups (COGs) using BGI (Beijing Genomics Institute, China) to create a fungus-specific database (Additional file 2: FW16.IntegrationTable.lxs). The genome sequence of *F. metavorans* FW16.1 was deposited in GenBank (bioproject number PRJNA413482, biosample number SAMN07749916) with the accession number JADNRB000000000.

The ITS-1/8S rRNA/ITS-2 region was amplified and sequenced using primers ITS1_fw (5′-TCC GTA GGT GAA CCT GCG G-3′) and ITS4_rv (5′-TCC TCC GCT TAT TGA TAT GC-3′) [92] and the ITS sequence was deposited in GenBank (accession number MG098676). Multiple sequence alignments for marker genes *TEF1*, *RPB2* and ITS + 28S for 79 *Fusarium* taxa were kindly provided by Kerry O’Donnell (personal communication). We built three independent covariance models using cmbuild v1.1.3 in the Infernal package (https://doi.org/10.1093/bioinformatics/btt509) from the sequence alignments without consensus structure information (parameter -noss). The bit scores depend on multiple sequence alignment length (more precisely, the covariance model length), so we ran the ungapped alignment sequences against their covariance model (cmalign -noss -g) and obtained 639, 1668 and 981 bits as average scores for *TEF1*, *RPB2* and ITS + 28S, respectively. Given that a covariance model without a consensus structure is basically a hidden Markov model (HMM), we initially used hmmbuild and hmmsearch (www.hmmer.org) instead, but this did not yield hits with sufficient scores, most likely due to high penalties for the insertion of introns.

Using the covariance model for *TEF1*, we found a hit in scaffold2 at position 6,427,210–6,427,837 with 643 bits (slightly above average). The model for *RPB2* returned two partial hits in close proximity on the reverse strand of scaffold 3. Manual inspection revealed overlapping full models for those hits, but a 130-bp region (probably an intron) divided the region in half. Enforcing global alignment of the combined region 2,964,591–2,966,345 (cmalign -noss -g) resulted in a score of 1671 bits, which was above the expected average score.

The covariance model for the ITS + 28S region did not return significant hits, probably due to the omission of this region in the assembly, reflecting multiple gene copies and repetitive regions that complicated the coverage information [93]. We therefore used the covariance model to identify 5737 of the raw 465,771 PacBio reads with sufficient hits. We next used proovread v2.14.1 (https://doi.org/10.1093/bioinformatics/btu392) to polish the frequent insertion or deletion of bases (indels) in the PacBio reads with short Illumina reads and mapped the results against the FW16.1 scaffold using bowtie2 v2.3.4.1 (https://doi.org/10.1038/nmeth.1923) with the settings-p 20-very-sensitive-local-f), yielding a clear pile up on scaffold 1 from 2,806,169 to 2,812,396. The local alignment of 2245 polished reads against the model (cmalign -mxsize 100,000 -noss) resulted in 456 high-scoring identical alignment rows of 1227 bp. Finally, we added the three identified marker gene regions to the initial sequence alignment (Additional file 3: 79-FSSC-3-locus.nex) and used IQTree v1.6.12 (https://doi.org/10.1093/molbev/msaa015) with the settings -nt AUTO -bb 5000, and partitions *TEF1* = 1–665, ITS + 28S = 666–1621 and *RPB2* = 162–3209 to construct the phylogenetic tree with partitioned maximum likelihood bootstrapping. The resulting newick tree file (Additional file 4, tree_fw16 + 79.figtree) was rooted at NRRL 22,090 *F. iludens* and NRRL 22,632 *F. plagianthi* and colored using FigTree v1.4.4 (http://tree.bio.ed.ac.uk/software/figtree/).

### CAZyme analysis

All genomic regions marked as CDS in our de novo assembly were screened for homologs to families and subfamilies in the CAZyme database [66] using a combination of RAPSearch2 [94, 95] and hmmsearch from the HMMER package [96] as previously described [97]. The CAZyme families/subfamilies were represented by sequence members with different enzymatic activities, annotated as different EC numbers, thus a single homolog CDS can yield multiple EC annotations. To reduce EC number ambiguity, we used BLASTP (v2.9.0 +) to score the CDS identified by LC–MS/MS against all sequences of the homologous CAZyme family obtained from dbCAN2 (http://csbl.bmb.uga.edu/dbCAN/index.php) [98]. The CDS was only annotated with EC numbers of the top BLASTP hits for each protein. The corresponding descriptors of EC numbers were used as possible functions (Additional file 1: Table S4). CAZymes identified by LC–MS/MS were mapped to the genome.

### Secretome analysis and SDS-PAGE

The *F. metavorans* secretomes were induced by fermentation in 100-mL Erlenmeyer flasks at 28 °C for up to 96 h, shaking at 150 rpm. Each liquid fermentation was carried out in duplicate (agro-residual biomass) or triplicate (synthetic substrates). Mycelia were pre-cultivated in YPD medium at 28 °C for 3 days, shaking at 150 rpm, then washed briefly and dried between sheets of filter paper (Whatman, Dassel, Germany). We then incubated 0.1 g of the semi-dried mycelia with 50 mL inductive medium at 28 °C for 72 h, shaking at 150 rpm. The inductive medium comprised mineral salts (0.35% NaNO_2_, 0.15% K_2_HPO_4_, 0.05% MgSO_4_ × 7H_2_O, 0.05% KCl, 0.001% FeSO_4_ × 7H_2_O) supplemented with 1% (w/v) synthetic or artificial cellulose (Avicel, α-cellulose, HEC, H-CMC, M-CMC or L-CMC, all from Sigma-Aldrich, Steinheim, Germany). The agro-residual biomass was prepared at a final concentration of 1% in Mandels and Weber medium [99], with additional yeast extract and peptone (0.03%). The sugarcane bagasse was milled to 1 mm and the maize leaves to 1.5 mm as untreated substrates. After 96 h, the fungal biomass was removed by centrifugation (3250 × *g* for 30 min) and the supernatant was harvested for secretome analysis, followed by lyophilization and resuspension in 50 mM citrate buffer (pH 4.5). The secretome samples were separated by SDS-PAGE on 12% polyacrylamide gels [100]. The gels were stained with 0.1% Coomassie Brilliant Blue R250 and destained with 45% methanol and 10% acetic acid. The gels were set aside for analysis by mass spectrometric proteomics and remaining samples were retained for enzymatic assays.

### Proteomics

#### Sample preparation

In-gel tryptic digestion [101] was carried out by dividing each gel lane into 4–5 equal parts and dicing them, followed by reduction with 10 mM dithiothreitol in 100 mM ammonium bicarbonate, alkylation with 55 mM iodoacetamide in 100 mM ammonium bicarbonate and digestion with 13 ng/µL trypsin in 10 mM ammonium bicarbonate containing 10% (v/v) acetonitrile (Promega, Mannheim, Germany)*.* Tryptic peptides were extracted with a 1:1 mixture of 5% formic acid and acetonitrile and were completely lyophilized. The peptides were resuspended in 40 µL 0.1% formic acid before LC–MS/MS analysis.

#### LC–MS/MS analysis of the tryptic peptides

We injected 2-µL samples onto an Acclaim PepMap C-18 nanoViper trapping column (Thermo Fisher Scientific, Waltham, MA, USA; 100 μm × 20 mm, 5 μm, 100 Å) at a flow rate of 3 μL/min and washed for 5 min with 2% buffer B (0.1% formic acid in acetonitrile). The peptides were separated on an Acclaim PepMap C-18 nanoViper reversed-phase capillary column (Thermo Fisher Scientific; 75 µm × 50 cm, 2 µm, 100 Å) at 45 °C using a Dionex Ultimate 3000 nano-UPLC system (Thermo Fisher Scientific) connected to a Fusion tribrid (quadrupole/Orbitrap/linear ion-trap) mass spectrometer (Thermo Fisher Scientific). The gradient system consisted of buffer A (0.1% formic acid in MS-grade water) and buffer B at a constant flow rate of 300 nL/min for 70 min. The profile was held at 3% B for 5 min followed by a gradient to 28% B, at 35 min, then 35% B at 40 min, and 90% B at 40 min 6 s. After a hold at 90% B for 9 min 54 s, the column was equilibrated at 3% B for 19 min 54 s. Eluted peptides were ionized in positive ion mode using a nanospray Flex with an electrospray ionization source (Thermo Fisher Scientific) and a fused-silica nano-bore emitter with an internal diameter of 10 μm (New Objective, Woburn, MA, USA) at a capillary voltage of 1800 V. The ion transfer tube temperature was set to 300 °C. Parent ion scans were carried out in the range 400–1300 *m/z* in the Orbitrap mass analyzer at 120 K resolution with a maximum injection time of 120 ms and an AGC target value of 2 × 10^5^. Data-dependent acquisition mode was set to top speed mode for precursor ion selection. The most intense peaks with (intensity threshold of 5 × 10^3^) were isolated with a quadrupole isolation width of 1.6 *m/z*, fragmented by high-energy collisional dissociation (collision energy 30%) and detected in the ion-trap mass analyzer. A dynamic exclusion filter was applied for 30 s and excluded after one time. For ion-trap detection, the scan rate was set to a rapid scan range 400–1300 *m/z*. The maximum injection time was 60 ms, and the AGC target value was 1 × 10^4^.

#### Protein identification by database matching

The LC–MS/MS data files were used to search the translated database of *F. metavorans* DSM105788 sequences (Additional file 2: FW16.IntegrationTable.lxs) with Proteome Discoverer v2.0 (Thermo Fisher Scientific) including the search engine Sequest HT. The search parameters included precursor and product ion mass tolerances of 10 ppm and 0.5 Da, respectively, two missed cleavages allowed, cysteine carbamidomethylation as a fixed modification, and methionine oxidation as a variable modification. Proteins found with at least one unique peptide and a false discovery rate (FDR) of 1% (determined by percolator) were accepted [101].

### Enzymatic activity

Enzymatic hydrolysis was measured using the DNS method [102] after liquid fermentation at 50 °C for 24 or 96 h with the substrates arabinan, arabinoxylan, galactan, xylan, starch, CMC and polygalacturonic acid (all at 0.5%) or pectin citrus and laminarin (at 0.2%). We mixed 10 µL of the *F. metavorans* extract with 50 µL of each substrate and 40 µL 50 mM citrate buffer (pH 4.8). Xylan was assayed for 10 min and the remaining substrates for 3 h. When *F. metavorans* was grown in YPD medium, we also measured CMCase activity against CMC every 24 h for up to 7 days. Furthermore, if the fungus was cultivated in Mandels’ mineral salts medium supplemented with 1% (w/v) cellulose and artificial cellulose substrates Avicel PH-101, α-cellulose, HEC, H-CMC, M-CMC or L-CMC, we also measured the CMCase activity on day 3. The protein concentration was determined using the ROTI Nanoquant protein detection kit (Carl Roth, Karlsruhe, Germany) by adding 50 μL of the supernatant to 200 μL of the detection solution. Measurements were collected from at last three experimental replicates.

### Saccharification of sugarcane bagasse and maize leaves

The conversion of 5% (w/v) NSCB, XSCB [44] and MZ into glucose, was tested in saturation curve assays supplemented with increasing amounts of the *F. metavorans* in-house crude enzymatic cocktail to a fixed amount of Accellerase 1500 (Genecor, Rochester, NY, USA) at final total cellulase activity of 5 FPU/g biomass, corresponding to 118 µg/mL. For the in-house enzymatic cocktail, the lyophilized secretome fractions from both biomass substrates were resuspended in 50 mM citrate buffer (pH 4.8) and combined at a 1:1 ratio (NSCB:MZ) before saturation curve experiments, such that the final protein concentration of 312 ± 2.7 µg/mL represented 100%. Saccharification was carried out in 2-mL Eppendorf tubes containing 50 mM citrate buffer (pH 4.5) and up to 70% (v/v) of the in-house enzymatic cocktail from *F.* *metavorans* at 50 °C for 24 h in a thermomixer (Eppendorf, Hamburg, Germany) at an agitation rate of 1000 rpm. The amount of protein applied for the saturation curve experiments can be found in Additional file 1: Table S5. Each experiment was replicated and the reducing sugars were measured in triplicate using the DNS assay [102]. Glucose standards were used to calibrate the glucose released under each condition. The statistical significance (threshold *p* < 0.05) was determined using Perseus (www.coxdocs.org/doku.php).

## Supplementary Information

**Additional file 1: Figure S1**: Genomic DNA from *Fusarium metavorans* FW16.1 (DSM105788) was isolated using the CTAB method and 5 μL was mixed with 6 × loading buffer (0.25% (w/v) xylene cyanol, 0.25% (w/v) bromophenol blue, 30% (v/v) glycerol) and separated by 0.8% (w/v) agarose gel electrophoresis in Tris–borate EDTA (TBE) buffer at 80 V for 60 min, with the GeneRuler 1 kb Plus DNA Ladder (Thermo Fisher Scientific) as a marker. The DNA was stained with 1% ethidium bromide for 15 min and observed on a UV transilluminator (SynGene Genius, BioImaging System). **Figure S2**: Specific CMCase activity of the supernatants against high-viscosity CMC over time in YPD medium. **Figure S3**: Specific CMCase activity of the supernatants using different synthetic nutrient sources. **Figure S4**: Enzymatic activities for polygalacturonase (A), laminarinase (B), CMCase (C) and xylanase (D). **Table S1**: CMCase activity of 48 fungal strains. **Table S2**: CAZyme analysis of fungal isolate FW16.1 and other fungal species. The coding regions were compared with the CAZyme database (Cantarel *et al*. 2009; Lombard *et al*. 2014). **Table S3**: Proteins of the fungal isolate FW16.1 induced on different synthetic and artificial cellulose and biomass substrates (maize leaves (MZ) or sugar cane bagasse (SCB)). The proteins were separated by SDS-PAGE followed by in-gel tryptic digestion and LC–MS/MS. The accession number, description, coverage (%), number of peptides (# peptides), peptide-to-spectrum matches (# PSMs), molecular weight in kDa (MW [kDa]), the calculated isoelectric point (calc. pI), Score Sequest HT and number of Peptides Sequest HT (# Peptides Sequest HT) were compared with the automated translation of the genome of the fungal isolate *Fusarium metavorans* DSM105788. BLASTP annotations and cellular functions are also shown. **Table S4**: Functional prediction of the CAZymes found on different synthetic and artificial cellulose and biomass substrates. **Table S5**: Quantity of protein applied in the saturation curve experiments.

**Additional file 2: **Integration table of the fungus.

**Additional file 3: **The phylogenetic tree provided by Dr. Kerry O’Donnell.

**Additional file 4: **The phylogenetic tree including the FW16.1 strain.

## Data Availability

All data generated or analyzed during this study are either included in this published article or can be found in the Supplementary Material.
