# Supplementary material for: Insights into the genome and secretome of Fusarium metavorans DSM105788 by cultivation on agro-residual biomass and synthetic nutrient sources
Source: Biotechnol Biofuels. 2021 Mar 20;14:74. doi: 10.1186/s13068-021-01927-9 (PMC7981871; doi:10.1186/s13068-021-01927-9)
Supplement: Supplementary file 1 — Additional file 1: Figure S1: Genomic DNA from Fusarium metavorans FW16.1 (DSM105788) was isolated using the CTAB method and 5 μL was mixed with 6 × loading buffer (0.25% (w/v) xylene cyanol, 0.25% (w/v) bromophenol blue, 30% (v/v) glycerol) and separated by 0.8% (w/v) agarose gel electrophoresis in Tris–borate EDTA (TBE) buffer at 80 V for 60 min, with the GeneRuler 1 kb Plus DNA Ladder (Thermo Fisher Scientific) as a marker. The DNA was stained with 1% ethidium bromide for 15 min and observed on a UV transilluminator (SynGene Genius, BioImaging System). Figure S2: Specific CMCase activity of the supernatants against high-viscosity CMC over time in YPD medium. Figure S3: Specific CMCase activity of the supernatants using different synthetic nutrient sources. Figure S4: Enzymatic activities for polygalacturonase (A), laminarinase (B), CMCase (C) and xylanase (D). Table S1: CMCase activity of 48 fungal strains. Table S2: CAZyme analysis of fungal isolate FW16.1 and other fungal species. The coding regions were compared with the CAZyme database (Cantarel et al. 2009; Lombard et al. 2014). Table S3: Proteins of the fungal isolate FW16.1 induced on different synthetic and artificial cellulose and biomass substrates (maize leaves (MZ) or sugar cane bagasse (SCB)). The proteins were separated by SDS-PAGE followed by in-gel tryptic digestion and LC–MS/MS. The accession number, description, coverage (%), number of peptides (# peptides), peptide-to-spectrum matches (# PSMs), molecular weight in kDa (MW [kDa]), the calculated isoelectric point (calc. pI), Score Sequest HT and number of Peptides Sequest HT (# Peptides Sequest HT) were compared with the automated translation of the genome of the fungal isolate Fusarium metavorans DSM105788. BLASTP annotations and cellular functions are also shown. Table S4: Functional prediction of the CAZymes found on different synthetic and artificial cellulose and biomass substrates. Table S5: Quantity of protein applied in the saturation cu [file 13068_2021_1927_MOESM1_ESM.docx]

Additional file 1

# Supplementary Data

The ITS region amplified using primers ITS1 and ITS4 (White *et al*., 1990) and sequenced using ABI Dye Terminator technology (Applied Biosystems, Foster City, CA, USA). The ITS sequence has been deposited in GenBank under accession number MG098676.

CCTGTGAACATACCTTCAACGTTGCCTCGGCGGGAACAGACGGCCCCGTGAAAACGGGCCGCCCCCGCCAGAGGACCCCCTAACTCTGTTTCTATAATGTTTCTTCTGAGTAAAACAAGCAAATAAATTAAAACTTTCAACAACGGATCTCTTGGCTCTGGCATCGATGAAGAACGCAGCGAAATGCGATAAGTAATGTGAATTGCAGAATTCAGTGAATCATCGAATCTTTGAACGCACATTGCGCCCGCCAGTATTCTGGCGGGCATGCCTGTTCGAGCGTCATTACAACCCTCAGGCCCCCGGGCCTGGCGTTGGGGATCGGCGGAGGGCCCCCCGTGGGCACACGCCGTCCCCCAAATACAGTGGCGGTCCCGCCGCAGCTTCCATCGCGTAGTAGCTAACACCTCGCGACGGAGAGCGGCGCGGCCACGCCGTAAAACCCCCAACTCTTCTGAAGTTGACCTCGAATCAGGTAGGAATACCCGCTGAACTTAAGCATA

# Supplementary Figures and Tables

## Supplementary Figures


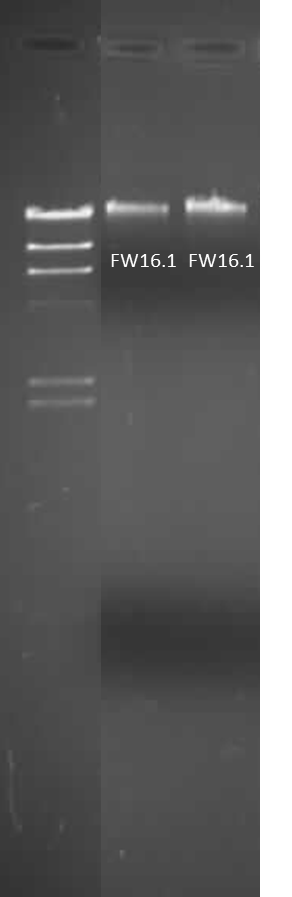


**Supplementary Figure 1:** Genomic DNA from *Fusarium metavorans* FW16.1 (DSM105788) was isolated using the CTAB method and 5 μL was mixed with 6 × loading buffer (0.25% (w/v) xylene cyanol, 0.25% (w/v) bromophenol blue, 30% (v/v) glycerol) and separated by 0.8% (w/v) agarose gel electrophoresis in Tris-borate EDTA (TBE) buffer at 80 V for 60 min, with the GeneRuler 1 kb Plus DNA Ladder (Thermo Fisher Scientific) as a marker. The DNA was stained with 1% ethidium bromide for 15 min and observed on a UV transilluminator (SynGene Genius, BioImaging System).


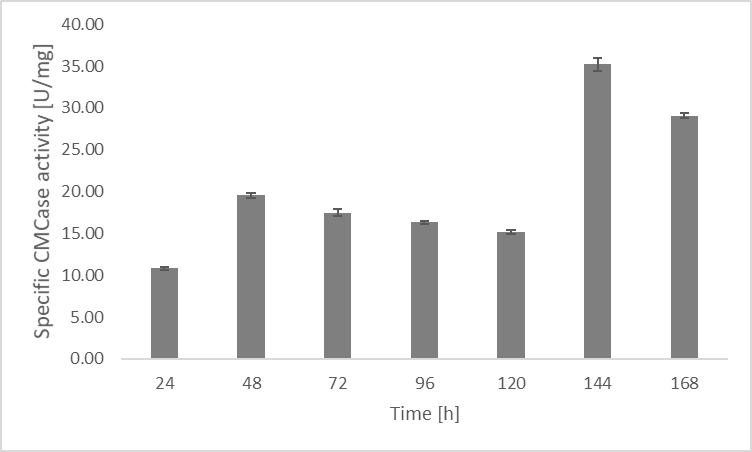


**Supplementary Figure 2:** Specific CMCase activity of the supernatants against high-viscosity CMC over time in YPD medium.

**Supplementary Figure 3:** Specific CMCase activity of the supernatants using different synthetic nutrient sources.


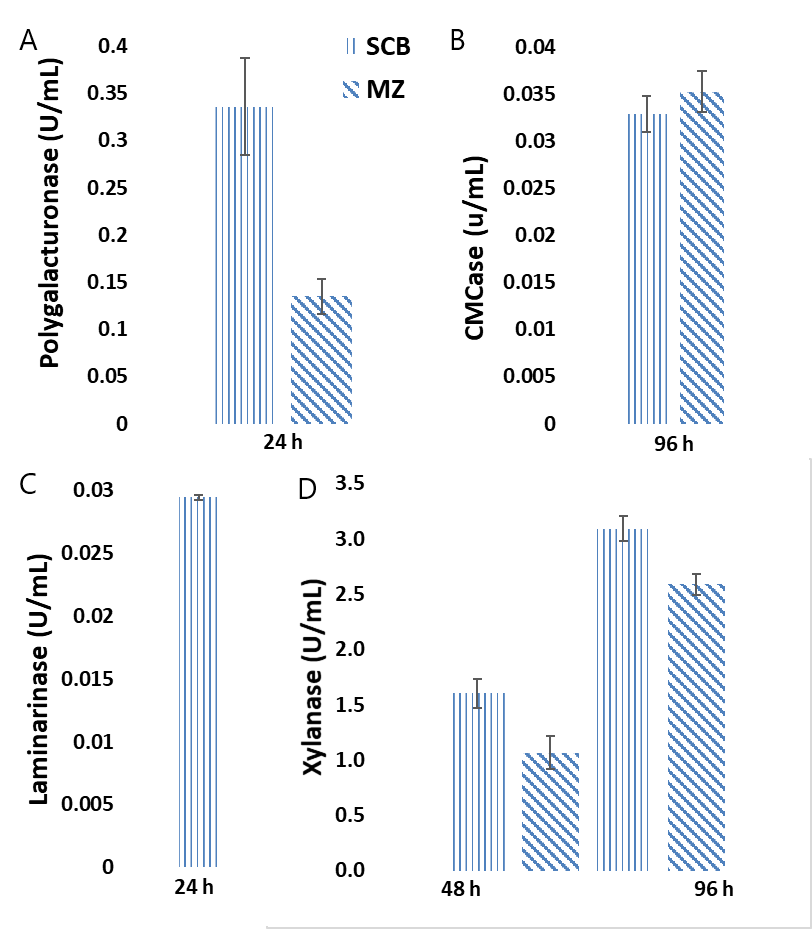


**Supplementary Figure 4:** Enzymatic activities for polygalacturonase (A), laminarinase (B), CMCase (C) and xylanase (D).

## Supplementary Tables

**Supplementary Table 1:** CMCase activity of 48 fungal strains.

| Working Name | DSMZ ID | CMCase activity | | | | | |
| --- | --- | --- | --- | --- | --- | --- | --- |
|  |  | Unit | | Unit/mL | | Unit/mg | |
|  |  | average | standard deviation | average | standard deviation | average | standard deviation |
| SF31 | 106393 | 0.001832 | 7.66 × 10^-5^ | 0.00916 | 0.000383 | 0.094524 | 0.003954 |
| Fi14 | 106457 | 0.00168 | 0.00006 | 0.00842 | 0.00029 | 0.06132 | 0.00211 |
| Fsh13 | 107512 | 0.00564 | 0.00002 | 0.02818 | 0.00010 | 0.05947 | 0.00022 |
| SF14 | 106375 | 0.00726 | 0.00001 | 0.03628 | 0.00003 | 0.05773 | 0.00008 |
| FW16.1 | 105788 | 0.00588 | 0.00001 | 0.02941 | 0.00007 | 0.05457 | 0.00013 |
| FL100 | 106490 | 0.00205 | 0.00003 | 0.01023 | 0.00014 | 0.04506 | 0.00036 |
| Fsh102 | 105790 | 0.00188 | 0.00004 | 0.00942 | 0.00019 | 0.02787 | 0.00052 |
| FL101 | 106261 | 0.00205 | 0.00002 | 0.01026 | 0.00011 | 0.02759 | 0.00030 |
| Fsh101 | 107513 | 0.00190 | 0.00002 | 0.00951 | 0.00009 | 0.02496 | 0.00039 |
| FL10 | 106248 | 0.00196 | 0.00001 | 0.00982 | 0.00006 | 0.02318 | 0.00043 |
| Fi5 | 106446 | 0.00497 | 0.00015 | 0.02487 | 0.00077 | 0.02292 | 0.00071 |
| FW2.1 | 106677 | 0.00163 | 0.00003 | 0.00813 | 0.00014 | 0.02281 | 0.00048 |
| FW27 | 106683 | 0.00084 | 0.00001 | 0.00420 | 0.00007 | 0.02252 | 0.00035 |
| SF19.1 | 106380 | 0.00226 | 0.00001 | 0.01131 | 0.00003 | 0.02035 | 0.00006 |
| Fsh201 | 107514 | 0.00246 | 0.00001 | 0.01230 | 0.00005 | 0.02002 | 0.00007 |
| Fi12 | 106455 | 0.00194 | 0.00001 | 0.00970 | 0.00004 | 0.01998 | 0.00009 |
| Fsh17 | 106723 | 0.00938 | 0.00001 | 0.04690 | 0.00003 | 0.01931 | 0.00002 |
| Fi43 | 106485 | 0.00263 | 0.00001 | 0.01313 | 0.00007 | 0.01846 | 0.00010 |
| FH101 | 106237 | 0.00255 | 0.00014 | 0.01277 | 0.00068 | 0.01718 | 0.00092 |
| Fi6.1 | 106243 | 0.00105 | 0.00001 | 0.00523 | 0.00004 | 0.01682 | 0.00013 |
| FF1 | 104516 | 0.00190 | 0.00004 | 0.00952 | 0.00018 | 0.01551 | 0.00029 |
| Fi4 | 106445 | 0.00212 | 0.00002 | 0.01059 | 0.00012 | 0.01528 | 0.00017 |
| Fsh20 | 106730 | 0.00082 | 0.00005 | 0.00410 | 0.00026 | 0.01527 | 0.00110 |
| FW35 | 106735 | 0.00211 | 0.00008 | 0.01057 | 0.00039 | 0.01450 | 0.00051 |
| Fi24 | 106466 | 0.00323 | 0.00002 | 0.01616 | 0.00008 | 0.01342 | 0.00006 |
| Fi39 | 106482 | 0.00258 | 0.00002 | 0.01292 | 0.00011 | 0.01331 | 0.00012 |
| FR1 | 106296 | 0.00240 | 0.00004 | 0.01200 | 0.00019 | 0.01326 | 0.00025 |
| FW36 | 106736 | 0.00192 | 0.00007 | 0.00961 | 0.00035 | 0.01136 | 0.00041 |
| Fi1 | 106442 | 0.00293 | 0.00006 | 0.01464 | 0.00031 | 0.01048 | 0.00023 |
| Fi7 | 106449 | 0.00205 | 0.00006 | 0.01026 | 0.00029 | 0.01036 | 0.00029 |
| Fi10 | 106453 | 0.00118 | 0.00005 | 0.00592 | 0.00027 | 0.00992 | 0.00046 |
| Fi23 | 106465 | 0.00067 | 0.00008 | 0.00333 | 0.00040 | 0.00765 | 0.00092 |
| Fi19 | 106461 | 0.00272 | 0.00006 | 0.01358 | 0.00029 | 0.00693 | 0.00015 |
| Fsh200 | 107515 | 0.00143 | 0.00006 | 0.00715 | 0.00032 | 0.00582 | 0.00026 |
| FL6 | 106243 | 0.00022 | 0.00018 | 0.00110 | 0.00091 | 0.00519 | 0.00428 |
| FL15 | 106312 | 0.00086 | 0.00003 | 0.00430 | 0.00016 | 0.00467 | 0.00019 |
| SF25 | 106387 | 0.00070 | 0.00007 | 0.00348 | 0.00036 | 0.00422 | 0.00041 |
| FW49 | 106685 | 0.00057 | 0.00020 | 0.00286 | 0.00102 | 0.00401 | 0.00172 |
| SF7 | 106361 | 0.00038 | 0.00042 | 0.00191 | 0.00208 | 0.00235 | 0.00445 |

## Supplementary Table 2: CAZyme analysis of fungal isolate FW16.1 and other fungal species. The coding regions were compared with the CAZyme database (Cantarel *et al*. 2009; Lombard *et al*. 2014).

| CAZyme | *Fusarium metavorans* DSM105788 FW16.1 | *Nectria haematococca* mpVI_77_13_4 | *Fusarium oxysporum* | *Fusarium verticillioides* 7600 | *Fusarium graminearum* PH_1 | *Trichoderma reesei* QM6a |
| --- | --- | --- | --- | --- | --- | --- |
| GH1 | 9 | 5 | 6 | 6 | 3 | 2 |
| GH2 | 11 | 11 | 8 | 10 | 10 | 7 |
| GH3 | 36 | 38 | 32 | 28 | 21 | 13 |
| GH4 | 0 | 0 | 0 | 0 | 0 | 0 |
| GH5 | 16 | 15 | 19 | 20 | 12 | 6 |
| GH6 | 0 | 0 | 0 | 0 | 0 | 0 |
| GH7 | 1 | 1 | 2 | 2 | 1 | 0 |
| GH9 | 0 | 0 | 0 | 0 | 0 | 0 |
| GH10 | 2 | 3 | 4 | 3 | 4 | 1 |
| GH11 | 2 | 3 | 3 | 4 | 2 | 3 |
| GH12 | 5 | 6 | 4 | 4 | 4 | 2 |
| GH13 | 8 | 8 | 10 | 13 | 7 | 4 |
| GH15 | 1 | 1 | 1 | 1 | 1 | 2 |
| GH16 | 21 | 20 | 27 | 30 | 21 | 14 |
| GH17 | 6 | 6 | 6 | 7 | 6 | 4 |
| GH18 | 13 | 18 | 24 | 16 | 14 | 14 |
| GH19 | 0 | 0 | 0 | 0 | 0 | 0 |
| GH20 | 2 | 4 | 4 | 3 | 3 | 3 |
| GH23 | 0 | 0 | 0 | 0 | 0 | 2 |
| GH24 | 2 | 3 | 1 | 1 | 0 | 1 |
| GH25 | 0 | 0 | 0 | 0 | 0 | 1 |
| GH26 | 0 | 0 | 0 | 0 | 0 | 0 |
| GH27 | 2 | 1 | 2 | 1 | 2 | 3 |
| GH28 | 10 | 11 | 15 | 9 | 6 | 4 |
| GH29 | 0 | 0 | 2 | 2 | 1 | 0 |
| GH30 | 0 | 0 | 3 | 2 | 0 | 5 |
| GH31 | 9 | 8 | 9 | 7 | 7 | 3 |
| GH32 | 4 | 5 | 12 | 7 | 5 | 0 |
| GH33 | 2 | 3 | 1 | 1 | 1 | 0 |
| GH35 | 7 | 8 | 6 | 3 | 3 | 1 |
| GH36 | 3 | 2 | 2 | 5 | 3 | 2 |
| GH37 | 2 | 2 | 2 | 2 | 2 | 2 |
| GH38 | 1 | 1 | 1 | 2 | 1 | 1 |
| GH39 | 2 | 1 | 3 | 3 | 1 | 1 |
| GH42 | 0 | 0 | 0 | 0 | 0 | 0 |
| GH43 | 41 | 43 | 53 | 36 | 25 | 6 |
| GH45 | 0 | 1 | 0 | 0 | 1 | 0 |
| GH46 | 0 | 0 | 0 | 0 | 0 | 0 |
| GH47 | 11 | 10 | 10 | 14 | 10 | 8 |
| GH49 | 1 | 1 | 1 | 1 | 0 | 0 |
| GH51 | 4 | 4 | 3 | 2 | 2 | 0 |
| GH53 | 1 | 1 | 2 | 1 | 1 | 0 |
| GH54 | 0 | 0 | 0 | 0 | 0 | 0 |
| GH55 | 3 | 4 | 7 | 8 | 4 | 6 |
| GH62 | 2 | 2 | 1 | 1 | 1 | 1 |
| GH63 | 1 | 1 | 1 | 1 | 1 | 1 |
| GH64 | 2 | 2 | 8 | 2 | 2 | 3 |
| GH65 | 0 | 0 | 1 | 1 | 0 | 2 |
| GH67 | 0 | 0 | 2 | 3 | 1 | 1 |
| GH71 | 4 | 5 | 2 | 3 | 1 | 2 |
| GH72 | 2 | 2 | 2 | 4 | 2 | 5 |
| GH73 | 0 | 0 | 0 | 0 | 0 | 0 |
| GH74 | 1 | 1 | 1 | 1 | 1 | 0 |
| GH75 | 2 | 2 | 2 | 4 | 1 | 3 |
| GH76 | 8 | 8 | 12 | 12 | 8 | 8 |
| GH78 | 16 | 12 | 19 | 9 | 7 | 1 |
| GH79 | 4 | 1 | 6 | 2 | 0 | 4 |
| GH81 | 1 | 1 | 1 | 7 | 1 | 2 |
| GH84 | 0 | 0 | 0 | 0 | 0 | 0 |
| GH85 | 0 | 0 | 0 | 0 | 0 | 0 |
| GH88 | 5 | 5 | 3 | 2 | 1 | 0 |
| GH89 | 0 | 0 | 0 | 0 | 0 | 2 |
| GH92 | 0 | 0 | 0 | 0 | 0 | 7 |
| GH93 | 4 | 4 | 5 | 5 | 2 | 0 |
| GH94 | 0 | 0 | 0 | 0 | 0 | 0 |
| GH95 | 3 | 3 | 2 | 1 | 2 | 4 |
| GH97 | 0 | 0 | 1 | 1 | 1 | 0 |
| GH105 | 4 | 4 | 4 | 3 | 3 | 1 |
| GH106 | 1 | 1 | 0 | 0 | 0 | 0 |
| GH109 | 0 | 0 | 0 | 0 | 1 | 0 |
| GH114 | 4 | 4 | 3 | 11 | 2 | 0 |
| GH115 | 3 | 2 | 3 | 2 | 2 | 1 |
| GH125 | 2 | 2 | 4 | 3 | 3 | 2 |
| GH127 | 3 | 3 | 2 | 2 | 1 | 0 |
| GH128 | 4 | 4 | 4 | 5 | 4 | 4 |
| GH130 | 0 | 0 | 0 | 0 | 0 | 0 |
| GH131 | 1 | 1 | 0 | 1 | 1 | 0 |
| GH132 | 2 | 2 | 2 | 6 | 2 | 2 |
| GH134 | 1 | 1 | 1 | 1 | 0 | 0 |
| GH135 | 2 | 2 | 0 | 0 | 0 | 0 |
| GH0 | 2 | 3 | 6 | 3 | 2 | 3 |
| GH0_ | 0 | 0 | 1 | 0 | 0 | 0 |
| GH2;CBM35 | 0 | 0 | 0 | 0 | 0 | 0 |
| GH5;CBM1 | 3 | 3 | 2 | 2 | 2 | 2 |
| GH5;CBM2;CBM63 | 0 | 0 | 1 | 1 | 0 | 0 |
| GH5;CBM63 | 1 | 1 | 1 | 1 | 2 | 0 |
| GH6;CBM1 | 1 | 1 | 1 | 1 | 1 | 1 |
| GH7;CBM1 | 2 | 2 | 1 | 2 | 1 | 3 |
| GH10;CBM1 | 1 | 0 | 2 | 2 | 1 | 0 |
| GH11;CBM1 | 1 | 0 | 0 | 0 | 0 | 0 |
| GH12;CBM1 | 0 | 0 | 0 | 0 | 0 | 0 |
| GH13;GT5 | 0 | 0 | 0 | 0 | 0 | 0 |
| GH13;CBM20 | 0 | 0 | 0 | 0 | 0 | 0 |
| GH13;CBM48 | 1 | 1 | 1 | 1 | 1 | 1 |
| GH15;CBM20 | 1 | 1 | 2 | 4 | 2 | 0 |
| GH16;CBM1 | 0 | 0 | 0 | 0 | 0 | 0 |
| GH16;CBM13 | 0 | 0 | 0 | 0 | 0 | 0 |
| GH16;CBM18 | 1 | 1 | 1 | 1 | 1 | 1 |
| GH16;CBM18;CBM18 | 0 | 0 | 0 | 0 | 0 | 0 |
| GH16;CBM6 | 0 | 0 | 0 | 0 | 0 | 0 |
| GH18;CBM1 | 0 | 0 | 0 | 0 | 0 | 2 |
| GH18;CBM18 | 4 | 4 | 1 | 5 | 1 | 0 |
| GH18;CBM18;CBM18 | 1 | 3 | 4 | 0 | 2 | 0 |
| GH18;CBM18;CBM50 | 4 | 3 | 3 | 1 | 1 | 3 |
| GH18;CBM19 | 0 | 0 | 0 | 0 | 0 | 0 |
| GH18;CBM50 | 0 | 0 | 0 | 2 | 1 | 0 |
| GH20;CBM32;CBM32 | 0 | 0 | 0 | 0 | 0 | 0 |
| GH25;CBM50 | 0 | 0 | 0 | 0 | 0 | 1 |
| GH26;CBM35 | 0 | 0 | 0 | 0 | 0 | 0 |
| GH27;CBM13 | 0 | 0 | 0 | 0 | 0 | 0 |
| GH27;CBM35 | 1 | 1 | 2 | 2 | 0 | 0 |
| GH28;CBM1 | 0 | 0 | 0 | 0 | 0 | 0 |
| GH30;CBM1 | 0 | 0 | 0 | 0 | 0 | 0 |
| GH31;GH18 | 0 | 0 | 0 | 0 | 0 | 0 |
| GH32;CBM38 | 1 | 1 | 4 | 1 | 0 | 0 |
| GH35;CBM32 | 0 | 0 | 0 | 0 | 0 | 0 |
| GH43;CBM35 | 0 | 0 | 0 | 0 | 0 | 0 |
| GH43;CBM1 | 0 | 0 | 0 | 0 | 0 | 0 |
| GH43;CBM1;CBM6 | 0 | 0 | 0 | 0 | 0 | 0 |
| GH43;CBM35 | 3 | 3 | 3 | 2 | 2 | 0 |
| GH43;CBM42 | 0 | 0 | 0 | 0 | 0 | 0 |
| GH43;CBM6 | 0 | 0 | 1 | 1 | 1 | 0 |
| GH43;CBM66 | 0 | 0 | 0 | 0 | 0 | 1 |
| GH45;CBM1 | 1 | 0 | 1 | 2 | 0 | 1 |
| GH46;CBM13 | 0 | 0 | 0 | 0 | 0 | 0 |
| GH54;CBM13 | 0 | 0 | 0 | 0 | 0 | 0 |
| GH54;CBM42 | 1 | 1 | 1 | 1 | 1 | 2 |
| GH55;CBM13 | 0 | 0 | 0 | 0 | 0 | 0 |
| GH55;CBM50 | 0 | 2 | 0 | 0 | 0 | 0 |
| GH62;CBM1 | 0 | 0 | 0 | 0 | 0 | 0 |
| GH62;CBM13 | 0 | 0 | 0 | 0 | 0 | 0 |
| GH71;CBM24 | 0 | 0 | 0 | 0 | 0 | 1 |
| GH71;CBM24;CBM24 | 2 | 1 | 1 | 1 | 0 | 1 |
| GH72;CBM43 | 1 | 1 | 1 | 1 | 1 | 0 |
| GH73;CBM50 | 0 | 0 | 0 | 0 | 0 | 0 |
| GH74;CBM1 | 1 | 0 | 0 | 0 | 0 | 1 |
| GH75;CBM13 | 0 | 0 | 0 | 0 | 0 | 0 |
| GH97;CBM13 | 0 | 0 | 0 | 0 | 0 | 0 |
| GH97;CBM51 | 0 | 0 | 0 | 0 | 0 | 0 |
| GH93;CBM66 | 0 | 0 | 0 | 0 | 0 | 0 |
| GH93;CBM13;CBM13 | 0 | 0 | 0 | 0 | 0 | 0 |
| GH131;CBM1 | 0 | 0 | 0 | 0 | 0 | 0 |
| GH133;GH13 | 0 | 0 | 0 | 0 | 0 | 0 |
| GH0;CBM13 | 0 | 0 | 0 | 0 | 1 | 0 |
| GH0;CBM50 | 0 | 0 | 0 | 0 | 0 | 0 |
| CE1 | 1 | 1 | 6 | 5 | 3 | 2 |
| CE2 | 1 | 1 | 1 | 1 | 1 | 0 |
| CE3 | 7 | 11 | 6 | 9 | 5 | 2 |
| CE4 | 5 | 5 | 6 | 6 | 7 | 2 |
| CE5 | 7 | 7 | 11 | 12 | 12 | 2 |
| CE6 | 18 | 18 | 11 | 11 | 9 | 7 |
| CE8 | 3 | 4 | 5 | 6 | 4 | 0 |
| CE9 | 2 | 2 | 1 | 2 | 1 | 1 |
| CE12 | 2 | 2 | 5 | 5 | 5 | 0 |
| CE15 | 0 | 0 | 0 | 0 | 0 | 0 |
| CE16 | 6 | 5 | 5 | 6 | 5 | 2 |
| CE0 | 2 | 2 | 2 | 3 | 2 | 1 |
| CE1;CBM1 | 0 | 0 | 0 | 0 | 0 | 0 |
| CE1;CBM2 | 0 | 0 | 0 | 0 | 0 | 0 |
| CE2;CBM1 | 0 | 0 | 0 | 0 | 0 | 0 |
| CE3;CBM1 | 0 | 0 | 0 | 0 | 0 | 0 |
| CE4;CBM18 | 0 | 0 | 0 | 0 | 1 | 1 |
| CE4;CBM18;CBM18 | 2 | 2 | 4 | 4 | 0 | 0 |
| CE4;CBM50 | 0 | 0 | 0 | 0 | 0 | 0 |
| CE5;CBM1 | 0 | 1 | 0 | 0 | 0 | 2 |
| CE15;CBM1 | 0 | 0 | 0 | 0 | 0 | 1 |
| CE16;CBM1 | 0 | 0 | 0 | 0 | 0 | 0 |
| CE0;CBM3 | 0 | 0 | 0 | 0 | 0 | 0 |
| PL1 | 12 | 12 | 9 | 10 | 8 | 0 |
| PL3 | 10 | 10 | 6 | 7 | 7 | 0 |
| PL4 | 5 | 6 | 3 | 3 | 3 | 0 |
| PL7 | 1 | 1 | 0 | 0 | 0 | 2 |
| PL8 | 0 | 0 | 0 | 0 | 0 | 1 |
| PL9 | 1 | 1 | 2 | 2 | 1 | 0 |
| PL11 | 1 | 1 | 1 | 0 | 0 | 0 |
| PL14 | 0 | 0 | 0 | 0 | 0 | 0 |
| PL20 | 1 | 1 | 0 | 0 | 1 | 2 |
| PL1;CBM1 | 2 | 2 | 1 | 1 | 1 | 0 |
| PL1;CBM35 | 0 | 0 | 0 | 0 | 0 | 0 |
| PL3;CBM1 | 1 | 1 | 1 | 0 | 0 | 0 |
| AA1 | 15 | 13 | 23 | 15 | 14 | 8 |
| AA2 | 4 | 4 | 8 | 5 | 5 | 3 |
| AA3 | 36 | 33 | 32 | 27 | 19 | 12 |
| AA4 | 6 | 7 | 5 | 2 | 1 | 1 |
| AA5 | 6 | 5 | 5 | 9 | 6 | 4 |
| AA6 | 1 | 1 | 1 | 2 | 1 | 1 |
| AA7 | 47 | 46 | 48 | 35 | 31 | 14 |
| AA8 | 3 | 2 | 2 | 2 | 1 | 0 |
| AA9 | 10 | 10 | 14 | 11 | 10 | 1 |
| AA11 | 5 | 5 | 4 | 5 | 5 | 3 |
| AA12 | 1 | 1 | 3 | 9 | 2 | 1 |
| AA13 | 0 | 0 | 2 | 1 | 1 | 0 |
| AA0 | 8 | 6 | 6 | 8 | 4 | 2 |
| AA3;CBM1 | 0 | 0 | 1 | 1 | 1 | 0 |
| AA5;CBM32 | 1 | 2 | 3 | 3 | 3 | 0 |
| AA3;AA8 | 0 | 0 | 0 | 0 | 0 | 0 |
| AA5;CBM32 | 0 | 0 | 0 | 0 | 0 | 0 |
| AA7;CBM18 | 0 | 0 | 0 | 0 | 0 | 0 |
| AA9;CBM1 | 2 | 2 | 2 | 2 | 2 | 1 |
| AA9;CBM18 | 1 | 1 | 1 | 1 | 1 | 1 |
| AA10;CBM2 | 0 | 0 | 0 | 0 | 0 | 0 |
| AA10;CBM12 | 0 | 0 | 2 | 0 | 0 | 0 |
| AA13;CBM20 | 1 | 1 | 0 | 0 | 0 | 0 |
| CBM9 | 1 | 0 | 0 | 0 | 0 | 0 |
| CBM13 | 0 | 0 | 0 | 0 | 0 | 0 |
| CBM18 | 0 | 0 | 0 | 0 | 0 | 0 |
| CBM21 | 0 | 0 | 0 | 0 | 0 | 0 |
| CBM50 | 0 | 0 | 0 | 0 | 0 | 0 |
| CBM63 | 1 | 0 | 0 | 0 | 0 | 0 |
| GT1 | 18 | 17 | 20 | 13 | 14 | 5 |
| GT2 | 9 | 9 | 10 | 15 | 9 | 8 |
| GT3 | 1 | 1 | 1 | 3 | 1 | 1 |
| GT4 | 7 | 8 | 7 | 10 | 7 | 5 |
| GT8 | 6 | 6 | 12 | 9 | 6 | 3 |
| GT15 | 5 | 5 | 4 | 5 | 4 | 4 |
| GT17 | 1 | 1 | 2 | 2 | 1 | 1 |
| GT18 | 0 | 0 | 0 | 0 | 0 | 0 |
| GT20 | 3 | 3 | 3 | 8 | 3 | 4 |
| GT21 | 1 | 1 | 1 | 1 | 1 | 1 |
| GT22 | 4 | 4 | 4 | 5 | 4 | 4 |
| GT24 | 1 | 1 | 1 | 2 | 1 | 1 |
| GT25 | 0 | 0 | 0 | 0 | 0 | 1 |
| GT31 | 0 | 0 | 0 | 0 | 0 | 0 |
| GT32 | 7 | 9 | 6 | 6 | 4 | 6 |
| GT33 | 1 | 1 | 1 | 1 | 1 | 1 |
| GT34 | 2 | 2 | 3 | 5 | 2 | 2 |
| GT35 | 1 | 1 | 1 | 2 | 1 | 1 |
| GT39 | 3 | 3 | 3 | 4 | 3 | 3 |
| GT41 | 3 | 2 | 4 | 2 | 2 | 1 |
| GT47 | 0 | 0 | 0 | 0 | 0 | 0 |
| GT48 | 2 | 2 | 1 | 1 | 1 | 1 |
| GT50 | 1 | 1 | 1 | 1 | 1 | 1 |
| GT51 | 0 | 0 | 0 | 0 | 0 | 0 |
| GT55 | 0 | 0 | 0 | 0 | 0 | 0 |
| GT57 | 2 | 2 | 2 | 2 | 2 | 2 |
| GT58 | 1 | 1 | 1 | 1 | 1 | 1 |
| GT59 | 1 | 1 | 1 | 1 | 1 | 1 |
| GT62 | 3 | 3 | 3 | 4 | 3 | 3 |
| GT64 | 2 | 2 | 2 | 1 | 2 | 2 |
| GT66 | 1 | 1 | 1 | 1 | 1 | 1 |
| GT69 | 6 | 5 | 4 | 5 | 2 | 4 |
| GT71 | 3 | 3 | 3 | 2 | 4 | 1 |
| GT76 | 1 | 1 | 1 | 1 | 1 | 1 |
| GT90 | 5 | 5 | 6 | 7 | 6 | 6 |
| GT91 | 0 | 0 | 0 | 0 | 0 | 0 |
| GT0 | 2 | 2 | 2 | 3 | 2 | 2 |

**Supplementary Table 3:** Proteins of the fungal isolate FW16.1 induced on different synthetic and artificial cellulose and biomass substrates (maize leaves (MZ) or sugar cane bagasse (SCB)). The proteins were separated by SDS-PAGE followed by in-gel tryptic digestion and LC-MS/MS. The accession number, description, coverage (%), number of peptides (# peptides), peptide-to-spectrum matches (# PSMs), molecular weight in kDa (MW [kDa]), the calculated isoelectric point (calc. pI), Score Sequest HT and number of Peptides Sequest HT (# Peptides Sequest HT) were compared with the automated translation of the genome of the fungal isolate *Fusarium metavorans* DSM105788. BLASTP annotations and cellular functions are also shown.

| No | Accession | Coverage % | No. of Peptides | No. of PSMs | No. of Unique Peptides | Score Sequest HT | pI | MW kDa | L-CMC | M-CMC | H-CMC | HEC | α-Cellulose | Avicel PH-101 | MZ | SCB | Function | BLAST hit > 70% identity | CAZYme | Function of CAZyme |
| --- | --- | --- | --- | --- | --- | --- | --- | --- | --- | --- | --- | --- | --- | --- | --- | --- | --- | --- | --- | --- |
| 1 | FW16_GLEAN_10000066 | 2 | 1 | 4 | 1 | 15.43 | 5.813627 | 96.54 |  |  |  |  |  |  | FW16_GLEAN_10000066 | FW16_GLEAN_10000066 | CARBOHYDRATE METABOLISM |  | GH2 | β-galactosidase |
| 2 | FW16_GLEAN_10000067 | 11 | 2 | 11 | 2 | 37.72 | 5.402188 | 41.38 |  |  |  |  |  |  | FW16_GLEAN_10000067 |  | LIPID METABOLIC | SGNH hydrolase-type esterase |  |  |
| 3 | FW16_GLEAN_10000102 | 13 | 2 | 13 | 2 | 43.86 | 3.751401 | 24.47 |  |  |  |  |  |  | FW16_GLEAN_10000102 | FW16_GLEAN_10000102 | NONE CONSERVED DOMAIN |  |  |  |
| 4 | FW16_GLEAN_10000141 | 24 | 4 | 26 | 4 | 108.09 | 5.68611 | 22.37 |  |  | FW16_GLEAN_10000141 |  | FW16_GLEAN_10000141 | FW16_GLEAN_10000141 |  | FW16_GLEAN_10000141 | CARBOHYDRATE METABOLISM |  | PL3 | pectate lyase |
| 5 | FW16_GLEAN_10000164 | 2 | 1 | 3 | 1 | 9.15 | 3.996159 | 97.08 |  |  |  |  |  |  | FW16_GLEAN_10000164 | FW16_GLEAN_10000164 | CARBOHYDRATE METABOLISM |  | AA5 | glyoxal oxidase |
| 6 | FW16_GLEAN_10000205 | 2 | 1 | 4 | 1 | 14.71 | 7.441113 | 63.44 |  |  |  |  |  |  | FW16_GLEAN_10000205 |  | CARBOHYDRATE METABOLISM |  | AA3 | alcohol oxidase |
| 7 | FW16_GLEAN_10000207 | 19.5402 | 5 | 5 | 5 | 10.2692 | 5.931709 | 27.66 | FW16_GLEAN_10000207 | FW16_GLEAN_10000207 | FW16_GLEAN_10000207 | FW16_GLEAN_10000207 | FW16_GLEAN_10000207 |  |  |  | CARBOHYDRATE METABOLISM |  | PL20 | endo-β-1,4-glucuronan lyase |
| 8 | FW16_GLEAN_10000297 | 47 | 3 | 77 | 3 | 325.68 | 6.032429 | 11.46 |  |  |  |  |  |  | FW16_GLEAN_10000297 | FW16_GLEAN_10000297 | RNA METABOLIC | RNAse (RIBONUCLEASES) |  |  |
| 9 | FW16_GLEAN_10000307 | 27.8912 | 4 | 5 | 4 | 9.94457 | 9.587687 | 13.46 | FW16_GLEAN_10000307 | FW16_GLEAN_10000307 | FW16_GLEAN_10000307 | FW16_GLEAN_10000307 | FW16_GLEAN_10000307 | FW16_GLEAN_10000307 |  |  | NONE CONSERVED DOMAIN |  |  |  |
| 10 | FW16_GLEAN_10000319 | 40 | 11 | 255 | 11 | 1084.27 | 5.415751 | 48.20 |  |  |  |  |  |  | FW16_GLEAN_10000319 | FW16_GLEAN_10000319 | CARBOHYDRATE METABOLISM |  | GH28 | polygalacturonase |
| 11 | FW16_GLEAN_10000334 | 6.34921 | 1 | 1 | 1 | 1.97099 | 4.465128 | 25.45 | FW16_GLEAN_10000334 |  |  |  |  |  |  |  | CARBOHYDRATE METABOLISM |  | CBM13 |  |
| 12 | FW16_GLEAN_10000346 | 41 | 10 | 162 | 10 | 613.64 | 5.214796 | 38.08 | FW16_GLEAN_10000346 | FW16_GLEAN_10000346 | FW16_GLEAN_10000346 | FW16_GLEAN_10000346 | FW16_GLEAN_10000346 | FW16_GLEAN_10000346 | FW16_GLEAN_10000346 | FW16_GLEAN_10000346 | PROTEOLYSIS | ZINC PEPTIDASE |  |  |
| 13 | FW16_GLEAN_10000393 | 17 | 7 | 139 | 7 | 560.75 | 6.588766 | 78.97 | FW16_GLEAN_10000393 |  | FW16_GLEAN_10000393 |  |  |  |  |  | LIPID METABOLIC |  |  |  |
| 14 | FW16_GLEAN_10000413 | 4.09836 | 1 | 1 | 1 | 1.65459 | 4.940285 | 24.45 |  |  |  | FW16_GLEAN_10000413 |  |  |  |  | OTHER BIOLOGICAL PROCESS | Necrosis inducing protein |  |  |
| 15 | FW16_GLEAN_10000416 | 60 | 15 | 1374 | 12 | 5881.11 | 4.782263 | 40.46 | FW16_GLEAN_10000416 | FW16_GLEAN_10000416 | FW16_GLEAN_10000416 | FW16_GLEAN_10000416 | FW16_GLEAN_10000416 | FW16_GLEAN_10000416 |  |  | CARBOHYDRATE METABOLISM |  | GH5+CBM1 | endo-β-1,4-glucanase / cellulase and other |
| 16 | FW16_GLEAN_10000428 | 4 | 1 | 3 | 1 | 7.58 | 5.600838 | 32.78 |  |  |  |  |  |  | FW16_GLEAN_10000428 | FW16_GLEAN_10000428 | PROTEOLYSIS | leucyl aminopeptidase |  |  |
| 17 | FW16_GLEAN_10000482 | 22.6027 | 3 | 3 | 3 | 6.81109 | 9.908813 | 13.07 | FW16_GLEAN_10000482 | FW16_GLEAN_10000482 | FW16_GLEAN_10000482 | FW16_GLEAN_10000482 | FW16_GLEAN_10000482 | FW16_GLEAN_10000482 |  |  | NONE CONSERVED DOMAIN |  |  |  |
| 18 | FW16_GLEAN_10000524 | 16 | 2 | 7 | 2 | 27.27 | 6.201336 | 16.37 |  |  |  |  |  |  | FW16_GLEAN_10000524 | FW16_GLEAN_10000524 | OTHER BIOLOGICAL PROCESS | NUCLEAR TRANSPORT FACTOR 2 (NTF2) |  |  |
| 19 | FW16_GLEAN_10000581 | 9.16335 | 1 | 2 | 1 | 7.1676 | 5.157518 | 24.59 |  |  | FW16_GLEAN_10000581 | FW16_GLEAN_10000581 | FW16_GLEAN_10000581 | FW16_GLEAN_10000581 |  |  | NONE CONSERVED DOMAIN |  |  |  |
| 20 | FW16_GLEAN_10000618 | 4 | 4 | 8 | 4 | 23.54 | 5.176893 | 112.42 |  |  |  |  |  |  | FW16_GLEAN_10000618 |  | CARBOHYDRATE METABOLISM |  | GH35 | β-galactosidase |
| 21 | FW16_GLEAN_10000631 | 74 | 32 | 2164 | 32 | 9485.29 | 5.444353 | 75.40 | FW16_GLEAN_10000631 | FW16_GLEAN_10000631 | FW16_GLEAN_10000631 | FW16_GLEAN_10000631 | FW16_GLEAN_10000631 | FW16_GLEAN_10000631 | FW16_GLEAN_10000631 | FW16_GLEAN_10000631 | CARBOHYDRATE METABOLISM |  | GH74 | endoglucanase |
| 22 | FW16_GLEAN_10000721 | 7 | 3 | 7 | 3 | 23.2 | 4.888127 | 65.47 |  |  |  |  |  |  | FW16_GLEAN_10000721 | FW16_GLEAN_10000721 | CARBOHYDRATE METABOLISM |  | AA3 | cellobiose dehydrogenase |
| 23 | FW16_GLEAN_10000788 | 1.93424 | 1 | 1 | 1 | 1.62306 | 6.065277 | 58.27 | FW16_GLEAN_10000788 |  |  |  |  |  |  |  | NONE CONSERVED DOMAIN |  |  |  |
| 24 | FW16_GLEAN_10000829 | 6 | 2 | 5 | 2 | 18.13 | 4.522344 | 52.89 |  |  |  |  |  |  |  | FW16_GLEAN_10000829 | CARBOHYDRATE METABOLISM |  | GH43 | β-xylosidase |
| 25 | FW16_GLEAN_10000830 | 2 | 1 | 5 | 1 | 18.99 | 6.359854 | 95.76 |  |  |  |  |  |  | FW16_GLEAN_10000830 | FW16_GLEAN_10000830 | CARBOHYDRATE METABOLISM | glycoside hydrolase family 43 [Fusarium beomiforme] | GH43 | β-xylosidase or α-L-arabinofuranosidase or xylanase or α-1,2-L-arabinofuranosidase or exo-α-1,5-L-arabinofuranosidase or exo-β-1,3-galactanase or β-D-galactofuranosidase |
| 26 | FW16_GLEAN_10000952 | 16 | 4 | 9 | 4 | 34.95 | 5.033305 | 47.53 |  |  |  |  |  |  | FW16_GLEAN_10000952 |  | CARBOHYDRATE METABOLISM |  | GH43-CBM35 SUB FAM 24 | β-xylosidase |
| 27 | FW16_GLEAN_10000995 | 7.89474 | 1 | 1 | 1 | 0 | 8.930134 | 58.96 |  |  |  | FW16_GLEAN_10000995 |  |  |  |  | OTHER BIOLOGICAL PROCESS | Phosphate transporter |  |  |
| 28 | FW16_GLEAN_10001019 | 5.14019 | 1 | 1 | 1 | 3.22841 | 4.99812 | 23.85 | FW16_GLEAN_10001019 |  |  |  | FW16_GLEAN_10001019 |  |  |  | OXIDATION-REDUCTION | Alkyl hydroperoxide reductase |  |  |
| 29 | FW16_GLEAN_10001025 | 26 | 9 | 135 | 9 | 521.28 | 4.653872 | 66.84 |  |  |  |  |  |  | FW16_GLEAN_10001025 | FW16_GLEAN_10001025 | PROTEOLYSIS | METALLOPROTEINASE/PEPTIDASE M36 |  |  |
| 30 | FW16_GLEAN_10001028 | 53 | 9 | 87 | 9 | 277.51 | 6.802727 | 31.58 |  |  |  |  |  |  | FW16_GLEAN_10001028 | FW16_GLEAN_10001028 | CARBOHYDRATE METABOLISM |  | PL1 | pectate lyase |
| 31 | FW16_GLEAN_10001075 | 5 | 1 | 1 | 1 | 3.41 | 5.483654 | 29.69 |  |  |  |  |  |  |  | FW16_GLEAN_10001075 | RNA METABOLIC | endonuclease exonuclease phosphatase family [Fusarium albosuccineum] |  |  |
| 32 | FW16_GLEAN_10001089 | 12.0787 | 3 | 3 | 3 | 4.03131 | 7.324425 | 38.44 | FW16_GLEAN_10001089 |  | FW16_GLEAN_10001089 |  |  |  |  |  | CARBOHYDRATE METABOLISM |  | CE2 | acetyl xylan esterase |
| 33 | FW16_GLEAN_10001114 | 7 | 1 | 2 | 1 | 7.3 | 3.402937 | 24.03 |  |  |  |  |  |  | FW16_GLEAN_10001114 |  | NONE CONSERVED DOMAIN | hypothetical protein FDECE_8968 [Fusarium decemcellulare] |  |  |
| 34 | FW16_GLEAN_10001218 | 14 | 5 | 28 | 3 | 94.15 | 4.945988 | 53.00 |  |  |  |  |  |  | FW16_GLEAN_10001218 |  | AMINO ACID METABOLISM | MPAO, PAO1 polyamine oxidase |  |  |
| 35 | FW16_GLEAN_10001242 | 6.30372 | 1 | 1 | 1 | 1.76461 | 6.285318 | 37.95 | FW16_GLEAN_10001242 |  |  |  |  |  |  |  | OXIDATION-REDUCTION | D-isomer specific 2-hydroxyacid dehydrogenase |  |  |
| 36 | FW16_GLEAN_10001275 | 9 | 4 | 9 | 4 | 24.36 | 5.733209 | 74.29 |  |  |  |  |  |  | FW16_GLEAN_10001275 | FW16_GLEAN_10001275 | CARBOHYDRATE METABOLISM |  | AA1 | Laccase / Laccase-like multicopper oxidase |
| 37 | FW16_GLEAN_10001346 | 38 | 4 | 108 | 4 | 554.64 | 8.491154 | 26.60 | FW16_GLEAN_10001346 | FW16_GLEAN_10001346 | FW16_GLEAN_10001346 | FW16_GLEAN_10001346 | FW16_GLEAN_10001346 | FW16_GLEAN_10001346 |  | FW16_GLEAN_10001346 | CARBOHYDRATE METABOLISM |  | AA9 | copper-dependent lytic polysaccharide monooxygenases |
| 38 | FW16_GLEAN_10001440 | 13 | 4 | 9 | 4 | 31.37 | 5.575658 | 48.46 |  |  |  |  |  |  |  | FW16_GLEAN_10001440 | CARBOHYDRATE METABOLISM |  | GH13 | α-Amylase |
| 39 | FW16_GLEAN_10001444 | 6.25 | 1 | 1 | 1 | 0 | 8.14109 | 51.93 |  |  |  |  |  |  |  |  | OTHER BIOLOGICAL PROCESS | Bifunctional solanapyrone synthase [Fusarium oxysporum f. sp. cubense] |  |  |
| 40 | FW16_GLEAN_10001451 | 8 | 2 | 4 | 2 | 11.61 | 4.459549 | 45.77 |  |  |  |  |  |  |  | FW16_GLEAN_10001451 | OTHER BIOLOGICAL PROCESS | ALPHA-BETA HYDROLASES |  |  |
| 41 | FW16_GLEAN_10001458 | 10 | 1 | 1 | 1 | 3.1 | 5.274008 | 21.85 |  |  |  |  |  |  |  | FW16_GLEAN_10001458 | NONE CONSERVED DOMAIN | uncharacterized protein NECHADRAFT_97189 [Fusarium vanettenii 77-13-4] |  |  |
| 42 | FW16_GLEAN_10001499 | 2.74314 | 1 | 1 | 1 | 0 | 6.43972 | 44.10 | FW16_GLEAN_10001499 |  |  |  |  |  |  |  | OXIDATION-REDUCTION | Cytochrome P450 |  |  |
| 43 | FW16_GLEAN_10001515 | 17 | 2 | 13 | 2 | 42.78 | 7.202629 | 16.33 |  |  |  |  |  |  | FW16_GLEAN_10001515 | FW16_GLEAN_10001515 | OTHER BIOLOGICAL PROCESS | FB-LECTIN/FUNGAL FRUIT BODY LECTIN |  |  |
| 44 | FW16_GLEAN_10001518 | 13 | 3 | 6 | 3 | 20.6 | 4.635194 | 34.69 |  |  |  |  |  |  | FW16_GLEAN_10001518 | FW16_GLEAN_10001518 | CARBOHYDRATE METABOLISM |  | GH43 | α-galactosidase |
| 45 | FW16_GLEAN_10001531 | 7 | 2 | 5 | 2 | 20.6 | 6.822216 | 53.44 |  |  |  |  |  |  |  | FW16_GLEAN_10001531 | OTHER BIOLOGICAL PROCESS | SUGAR TRANSPORTER LIKE |  |  |
| 46 | FW16_GLEAN_10001538 | 2 | 1 | 2 | 1 | 6.26 | 5.447366 | 76.43 |  |  |  |  |  |  | FW16_GLEAN_10001538 | FW16_GLEAN_10001538 | CARBOHYDRATE METABOLISM |  | GH28 | galacturan 1,4-alpha-galacturonidase |
| 47 | FW16_GLEAN_10001547 | 9 | 14 | 58 | 1 | 250.8 | 4.792706 | 262.14 |  |  |  |  |  |  | FW16_GLEAN_10001547 |  | CARBOHYDRATE METABOLISM |  | CE8 | Pectinesterase |
| 48 | FW16_GLEAN_10001573 | 58 | 17 | 749 | 17 | 3228.35 | 8.137892 | 37.49 | FW16_GLEAN_10001573 | FW16_GLEAN_10001573 | FW16_GLEAN_10001573 | FW16_GLEAN_10001573 | FW16_GLEAN_10001573 | FW16_GLEAN_10001573 | FW16_GLEAN_10001573 | FW16_GLEAN_10001573 | CARBOHYDRATE METABOLISM |  | GH10 | Xylanase |
| 49 | FW16_GLEAN_10001583 | 26.4706 | 2 | 2 | 2 | 4.3289 | 4.867138 | 10.98 |  |  |  | FW16_GLEAN_10001583 |  |  |  |  | NONE CONSERVED DOMAIN |  |  |  |
| 50 | FW16_GLEAN_10001593 | 15 | 7 | 35 | 7 | 117.81 | 4.381458 | 58.50 |  |  |  |  |  |  | FW16_GLEAN_10001593 | FW16_GLEAN_10001593 | CARBOHYDRATE METABOLISM |  | GH27-CBM35 | α-galactosidase |
| 51 | FW16_GLEAN_10001600 | 3 | 1 | 2 | 1 | 5.5 | 4.760895 | 56.09 |  |  |  |  |  |  | FW16_GLEAN_10001600 | FW16_GLEAN_10001600 | NONE CONSERVED DOMAIN | hypothetical protein CEP52_001089 [Fusarium sp. AF-4] |  |  |
| 52 | FW16_GLEAN_10001601 | 59 | 11 | 217 | 11 | 933.6 | 8.881646 | 33.05 |  |  |  |  |  |  | FW16_GLEAN_10001601 | FW16_GLEAN_10001601 | CARBOHYDRATE METABOLISM |  | CE8 | Pectinesterase |
| 53 | FW16_GLEAN_10001628 | 3 | 1 | 3 | 1 | 11.51 | 6.374161 | 39.92 |  |  |  |  |  |  | FW16_GLEAN_10001628 | FW16_GLEAN_10001628 | CARBOHYDRATE METABOLISM |  | PL9 | pectate lyase |
| 54 | FW16_GLEAN_10001686 | 32 | 4 | 91 | 4 | 319.17 | 5.743412 | 16.68 | FW16_GLEAN_10001686 | FW16_GLEAN_10001686 | FW16_GLEAN_10001686 | FW16_GLEAN_10001686 |  |  | FW16_GLEAN_10001686 | FW16_GLEAN_10001686 | OTHER BIOLOGICAL PROCESS | Cell wall mannoprotein 1 |  |  |
| 55 | FW16_GLEAN_10001748 | 1 | 1 | 1 | 1 | 2.54 | 4.215627 | 95.72 |  |  |  |  |  |  | FW16_GLEAN_10001748 |  | PROTEOLYSIS | Peptidase S8/S53 |  |  |
| 56 | FW16_GLEAN_10001789 | 1.89702 | 1 | 1 | 1 | 3.50554 | 5.738819 | 125.12 |  |  | FW16_GLEAN_10001789 |  |  |  |  |  | OTHER BIOLOGICAL PROCESS | Ankyrin repeat-containing domain |  |  |
| 57 | FW16_GLEAN_10001797 | 4 | 1 | 7 | 1 | 9.34 | 9.2859 | 25.12 |  |  |  | FW16_GLEAN_10001797 | FW16_GLEAN_10001797 |  |  |  | CARBOHYDRATE METABOLISM |  | GH12 | endoglucanase / xyloglucan hydrolase |
| 58 | FW16_GLEAN_10001805 | 7 | 2 | 14 | 2 | 46.48 | 4.456078 | 40.06 |  |  |  |  |  |  | FW16_GLEAN_10001805 | FW16_GLEAN_10001805 | CARBOHYDRATE METABOLISM |  | GH93 | exo-α-L-1,5-arabinanase |
| 59 | FW16_GLEAN_10001821 | 6 | 1 | 2 | 1 | 5.44 | 6.045744 | 33.72 |  |  |  |  |  |  | FW16_GLEAN_10001821 |  | CARBOHYDRATE METABOLISM |  | GH43 | β-xylosidase |
| 60 | FW16_GLEAN_10001859 | 8 | 4 | 23 | 3 | 54.35 | 5.447552 | 69.36 |  |  |  |  |  |  |  | FW16_GLEAN_10001859 | PROTEOLYSIS | ASPARTIC ENDOPEPTIDASE |  |  |
| 61 | FW16_GLEAN_10001866 | 14 | 2 | 5 | 2 | 13.39 | 4.737755 | 18.87 |  |  |  |  |  |  |  | FW16_GLEAN_10001866 | OTHER BIOLOGICAL PROCESS | ALPHABETAHYDROLASE_DUF3237 |  |  |
| 62 | FW16_GLEAN_10001888 | 1.56006 | 2 | 20 | 2 | 52.1078 | 4.150036 | 64.78 | FW16_GLEAN_10001888 | FW16_GLEAN_10001888 | FW16_GLEAN_10001888 | FW16_GLEAN_10001888 | FW16_GLEAN_10001888 | FW16_GLEAN_10001888 |  |  | CARBOHYDRATE METABOLISM |  | GH7-CBM1 | endo-β-1,4-glucanase |
| 63 | FW16_GLEAN_10001898 | 4 | 1 | 3 | 1 | 5.7 | 3.87903 | 18.89 |  |  |  |  |  |  | FW16_GLEAN_10001898 | FW16_GLEAN_10001898 | NONE CONSERVED DOMAIN | hypothetical protein CEP54_010099 [Fusarium sp. AF-8] |  |  |
| 64 | FW16_GLEAN_10001905 | 7.59494 | 1 | 1 | 1 | 2.91132 | 4.837361 | 42.94 |  |  |  | FW16_GLEAN_10001905 |  |  |  |  | NONE CONSERVED DOMAIN |  |  |  |
| 65 | FW16_GLEAN_10001928 | 1.91518 | 1 | 1 | 1 | 3.49166 | 5.826799 | 80.69 |  |  | FW16_GLEAN_10001928 |  |  |  |  |  | CARBOHYDRATE METABOLISM |  | AA2 | manganese peroxidase / versatile peroxidase / lignin peroxidase |
| 66 | FW16_GLEAN_10001932 | 33 | 6 | 46 | 6 | 191.81 | 6.644035 | 34.20 |  |  |  |  |  |  | FW16_GLEAN_10001932 | FW16_GLEAN_10001932 | CARBOHYDRATE METABOLISM |  | PL1 | pectate lyase |
| 67 | FW16_GLEAN_10001949 | 60 | 12 | 727 | 4 | 2904.15 | 6.892635 | 23.55 |  |  |  |  |  |  | FW16_GLEAN_10001949 | FW16_GLEAN_10001949 | CARBOHYDRATE METABOLISM |  | PL3 | pectate lyase |
| 68 | FW16_GLEAN_10001951 | 16 | 3 | 14 | 3 | 42.67 | 4.508378 | 37.29 |  |  |  |  |  |  |  | FW16_GLEAN_10001951 | PROTEOLYSIS | serine endopeptidase |  |  |
| 69 | FW16_GLEAN_10001958 | 1 | 1 | 15 | 1 | 35.67 | 5.577858 | 108.26 |  |  |  |  |  |  | FW16_GLEAN_10001958 |  | CARBOHYDRATE METABOLISM |  | GH115 | xylan α-1,2-glucuronidase or α-(4-O-methyl)-glucuronidase |
| 70 | FW16_GLEAN_10001962 | 19 | 9 | 29 | 9 | 101.6 | 5.516551 | 77.57 |  |  |  |  |  |  | FW16_GLEAN_10001962 | FW16_GLEAN_10001962 | CARBOHYDRATE METABOLISM |  | GH5 | endo-β-1,4-glucanase / cellulase and other |
| 71 | FW16_GLEAN_10002031 | 8 | 2 | 6 | 2 | 23.48 | 4.831693 | 50.73 |  |  |  |  |  |  |  | FW16_GLEAN_10002031 | PROTEOLYSIS | AMINOPEPTIDASE |  |  |
| 72 | FW16_GLEAN_10002039 | 4 | 1 | 1 | 1 | 3.83 | 6.059902 | 36.30 |  |  |  |  |  |  | FW16_GLEAN_10002039 |  | NONE CONSERVED DOMAIN | hypothetical protein BHE90_002908 [Fusarium euwallaceae] |  |  |
| 73 | FW16_GLEAN_10002083 | 0.577664 | 1 | 1 | 1 | 2.14993 | 5.893555 | 168.97 | FW16_GLEAN_10002083 |  |  |  |  |  |  |  | CARBOHYDRATE METABOLISM |  | GH18-CBM18-CBM50 | chitinase |
| 74 | FW16_GLEAN_10002117 | 4 | 1 | 21 | 1 | 78.11 | 3.956944 | 31.03 |  |  |  |  |  |  | FW16_GLEAN_10002117 | FW16_GLEAN_10002117 | NONE CONSERVED DOMAIN | hypothetical protein CDV31_006328 [Fusarium ambrosium] |  |  |
| 75 | FW16_GLEAN_10002182 | 2.02703 | 1 | 1 | 1 | 0 | 5.193701 | 65.97 |  |  |  | FW16_GLEAN_10002182 |  |  |  |  | NONE CONSERVED DOMAIN |  |  |  |
| 76 | FW16_GLEAN_10002187 | 21.0243 | 6 | 35 | 6 | 129.74 | 4.986008 | 39.36 | FW16_GLEAN_10002187 | FW16_GLEAN_10002187 | FW16_GLEAN_10002187 | FW16_GLEAN_10002187 |  |  | FW16_GLEAN_10002187 | FW16_GLEAN_10002187 | CARBOHYDRATE METABOLISM |  | AA13-CBM20 | copper-dependent lytic polysaccharide monooxygenases |
| 77 | FW16_GLEAN_10002223 | 4 | 1 | 1 | 1 | 2.83 | 4.715526 | 45.26 |  |  |  |  |  |  | FW16_GLEAN_10002223 | FW16_GLEAN_10002223 | PROTEOLYSIS | Aspartic peptidase |  |  |
| 78 | FW16_GLEAN_10002361 | 40 | 9 | 63 | 9 | 284.32 | 5.584733 | 50.84 | FW16_GLEAN_10002361 |  | FW16_GLEAN_10002361 |  |  |  | FW16_GLEAN_10002361 | FW16_GLEAN_10002361 | PROTEOLYSIS | aminopeptidase Y precursor vacuolar [Fusarium albosuccineum] |  |  |
| 79 | FW16_GLEAN_10002371 | 4 | 2 | 20 | 2 | 81.15 | 3.609186 | 79.39 |  |  |  |  |  |  | FW16_GLEAN_10002371 | FW16_GLEAN_10002371 | NONE CONSERVED DOMAIN |  |  |  |
| 80 | FW16_GLEAN_10002372 | 9.78593 | 6 | 6 | 6 | 9.57966 | 5.39625 | 68.98 | FW16_GLEAN_10002372 | FW16_GLEAN_10002372 | FW16_GLEAN_10002372 | FW16_GLEAN_10002372 |  |  |  |  | NONE CONSERVED DOMAIN |  |  |  |
| 81 | FW16_GLEAN_10002430 | 59 | 18 | 947 | 18 | 4125.91 | 5.61481 | 49.48 |  |  |  |  |  |  | FW16_GLEAN_10002430 | FW16_GLEAN_10002430 | CARBOHYDRATE METABOLISM |  | GH54 | α-L-arabinofuranosidase / β-xylosidase |
| 82 | FW16_GLEAN_10002444 | 16 | 1 | 1 | 1 | 3.36 | 5.725708 | 18.01 |  |  |  |  |  |  | FW16_GLEAN_10002444 | FW16_GLEAN_10002444 | NONE CONSERVED DOMAIN | hypothetical protein CDV36_007167 [Fusarium kuroshium] |  |  |
| 83 | FW16_GLEAN_10002565 | 8 | 5 | 21 | 5 | 70 | 4.360903 | 76.09 | FW16_GLEAN_10002565 |  | FW16_GLEAN_10002565 |  |  |  | FW16_GLEAN_10002565 | FW16_GLEAN_10002565 | CARBOHYDRATE METABOLISM |  | GH37 | α,α-trehalase |
| 84 | FW16_GLEAN_10002576 | 7.57238 | 3 | 4 | 3 | 12.3587 | 5.570699 | 48.91 | FW16_GLEAN_10002576 | FW16_GLEAN_10002576 | FW16_GLEAN_10002576 | FW16_GLEAN_10002576 | FW16_GLEAN_10002576 | FW16_GLEAN_10002576 |  |  | AMINO ACID METABOLISM | Adenosylhomocysteinase [Fusarium kuroshium] |  |  |
| 85 | FW16_GLEAN_10002712 | 11.6505 | 1 | 2 | 1 | 5.49938 | 11.80668 | 11.37 | FW16_GLEAN_10002712 | FW16_GLEAN_10002712 | FW16_GLEAN_10002712 | FW16_GLEAN_10002712 | FW16_GLEAN_10002712 | FW16_GLEAN_10002712 |  |  | OTHER BIOLOGICAL PROCESS | Histone H4 [Tolypocladium capitatum] |  |  |
| 86 | FW16_GLEAN_10002775 | 12.3077 | 3 | 4 | 3 | 12.0432 | 9.217985 | 28.50 | FW16_GLEAN_10002775 | FW16_GLEAN_10002775 | FW16_GLEAN_10002775 |  |  |  |  |  | PROTEIN BIOSYNTHESIS | 40s ribosomal s3 [Fusarium albosuccineum] |  |  |
| 87 | FW16_GLEAN_10002845 | 5.46875 | 1 | 1 | 1 | 1.67534 | 11.32776 | 14.47 |  |  | FW16_GLEAN_10002845 |  |  |  |  |  | OTHER BIOLOGICAL PROCESS | Histone H4 |  |  |
| 88 | FW16_GLEAN_10002861 | 2.09724 | 1 | 1 | 1 | 2.86407 | 6.271916 | 118.22 |  |  |  |  |  |  |  | FW16_GLEAN_10002861 | OTHER BIOLOGICAL PROCESS | sucA 2-oxoglutarate dehydrogenase E1 component |  |  |
| 89 | FW16_GLEAN_10002865 | 5 | 2 | 6 | 2 | 20.6 | 5.125607 | 55.15 | FW16_GLEAN_10002865 | FW16_GLEAN_10002865 | FW16_GLEAN_10002865 | FW16_GLEAN_10002865 | FW16_GLEAN_10002865 | FW16_GLEAN_10002865 | FW16_GLEAN_10002865 |  | OTHER BIOLOGICAL PROCESS | ATPase |  |  |
| 90 | FW16_GLEAN_10002866 | 2.12164 | 1 | 1 | 1 | 3.29879 | 5.669788 | 80.28 |  |  | FW16_GLEAN_10002866 |  |  |  |  |  | CARBOHYDRATE METABOLISM |  | GH13-CBM48 | α-amylase / pullulanase |
| 91 | FW16_GLEAN_10002874 | 2.77296 | 1 | 1 | 1 | 0 | 4.569734 | 64.62 | FW16_GLEAN_10002874 |  |  |  |  |  |  |  | PROTEIN BIOSYNTHESIS | spliceosome associated [Fusarium albosuccineum] |  |  |
| 92 | FW16_GLEAN_10002967 | 8.20106 | 1 | 1 | 1 | 0 | 9.281474 | 41.61 |  |  |  |  |  |  |  | FW16_GLEAN_10002967 | RNA METABOLIC | FHA domain-interacting nucleolar phosphoprotein |  |  |
| 93 | FW16_GLEAN_10002975 | 4.61538 | 1 | 1 | 1 | 2.66039 | 6.234785 | 21.97 | FW16_GLEAN_10002975 |  | FW16_GLEAN_10002975 | FW16_GLEAN_10002975 | FW16_GLEAN_10002975 | FW16_GLEAN_10002975 |  |  | OTHER BIOLOGICAL PROCESS | Small GTPase superfamily |  |  |
| 94 | FW16_GLEAN_10003075 | 46 | 5 | 59 | 5 | 268.08 | 6.050777 | 21.48 |  |  |  |  |  |  | FW16_GLEAN_10003075 | FW16_GLEAN_10003075 | OTHER BIOLOGICAL PROCESS | CERATO-PLATANIN FAMILY |  |  |
| 95 | FW16_GLEAN_10003104 | 25 | 6 | 90 | 6 | 345.9 | 4.538451 | 49.49 |  |  |  |  |  |  | FW16_GLEAN_10003104 | FW16_GLEAN_10003104 | CARBOHYDRATE METABOLISM |  | GH39 | α-L-iduronidase / β-xylosidase / α-L-arabinofuranosidase / β-glucosidase / β-galactosidase |
| 96 | FW16_GLEAN_10003157 | 4.58716 | 1 | 1 | 1 | 2.21116 | 6.041504 | 24.53 |  |  | FW16_GLEAN_10003157 |  |  |  |  |  | OTHER BIOLOGICAL PROCESS | Carbonic anhydrase |  |  |
| 97 | FW16_GLEAN_10003177 | 27.027 | 4 | 11 | 4 | 33.8465 | 9.685877 | 13.75 | FW16_GLEAN_10003177 | FW16_GLEAN_10003177 | FW16_GLEAN_10003177 | FW16_GLEAN_10003177 | FW16_GLEAN_10003177 | FW16_GLEAN_10003177 |  |  | NONE CONSERVED DOMAIN |  |  |  |
| 98 | FW16_GLEAN_10003221 | 15 | 1 | 3 | 1 | 10.4 | 3.920816 | 11.94 |  |  |  |  |  |  | FW16_GLEAN_10003221 |  | NONE CONSERVED DOMAIN | hypothetical protein CDV31_009772 [Fusarium ambrosium] |  |  |
| 99 | FW16_GLEAN_10003277 | 25 | 20 | 203 | 20 | 714.68 | 4.469946 | 117.52 |  |  |  |  |  |  | FW16_GLEAN_10003277 | FW16_GLEAN_10003277 | CARBOHYDRATE METABOLISM |  | GH115 | xylan α-1,2-glucuronidase/ α-(4-O-methyl)-glucuronidase |
| 100 | FW16_GLEAN_10003286 | 31 | 7 | 30 | 7 | 102.48 | 6.743193 | 31.70 | FW16_GLEAN_10003286 | FW16_GLEAN_10003286 |  |  |  |  |  | FW16_GLEAN_10003286 | CARBOHYDRATE METABOLISM |  | GH43 | β-xylosidase / α-L-arabinofuranosidase / xylanase |
| 101 | FW16_GLEAN_10003305 | 19 | 4 | 23 | 4 | 85.9 | 9.615482 | 34.26 |  |  |  |  |  |  |  | FW16_GLEAN_10003305 | CARBOHYDRATE METABOLISM |  | GH10 | endo-1,4-β-xylanase /; endo-1,3-β-xylanase |
| 102 | FW16_GLEAN_10003323 | 4.71204 | 1 | 1 | 1 | 1.93917 | 4.297175 | 19.58 |  |  |  |  |  |  | FW16_GLEAN_10003323 | FW16_GLEAN_10003323 | NONE CONSERVED DOMAIN | hypothetical protein CEP51_001944 [Fusarium floridanum] |  |  |
| 103 | FW16_GLEAN_10003329 | 26 | 8 | 35 | 8 | 136.75 | 4.897066 | 71.26 |  |  |  |  |  |  | FW16_GLEAN_10003329 | FW16_GLEAN_10003329 | CARBOHYDRATE METABOLISM |  | GH51 | endoglucanase / endo-β-1,4-xylanase |
| 104 | FW16_GLEAN_10003342 | 1.4157 | 1 | 1 | 1 | 0 | 3.574223 | 74.44 | FW16_GLEAN_10003342 |  |  |  |  |  |  |  | NONE CONSERVED DOMAIN |  |  |  |
| 105 | FW16_GLEAN_10003369 | 26 | 5 | 24 | 5 | 98.61 | 5.534708 | 38.83 | FW16_GLEAN_10003369 | FW16_GLEAN_10003369 | FW16_GLEAN_10003369 | FW16_GLEAN_10003369 | FW16_GLEAN_10003369 |  | FW16_GLEAN_10003369 | FW16_GLEAN_10003369 | OTHER BIOLOGICAL PROCESS | ALPHA/BETAHYDROLASE SUPERFAMILY |  |  |
| 106 | FW16_GLEAN_10003424 | 3.49515 | 1 | 1 | 1 | 3.17491 | 5.942049 | 55.54 | FW16_GLEAN_10003424 |  |  |  |  |  |  |  | AMINO ACID METABOLISM | Cysteine synthase/cystathionine beta-synthase |  |  |
| 107 | FW16_GLEAN_10003425 | 33 | 13 | 156 | 13 | 536.9 | 5.929961 | 51.51 |  |  | FW16_GLEAN_10003425 |  |  |  | FW16_GLEAN_10003425 | FW16_GLEAN_10003425 | PROTEOLYSIS | APE3 aminopeptidase Y 3.4.11.15 |  |  |
| 108 | FW16_GLEAN_10003449 | 45 | 7 | 92 | 7 | 396.63 | 4.244299 | 22.12 | FW16_GLEAN_10003449 |  | FW16_GLEAN_10003449 | FW16_GLEAN_10003449 | FW16_GLEAN_10003449 | FW16_GLEAN_10003449 | FW16_GLEAN_10003449 |  | NONE CONSERVED DOMAIN |  |  |  |
| 109 | FW16_GLEAN_10003484 | 25 | 5 | 24 | 5 | 96.4 | 4.981718 | 36.79 |  |  |  |  |  |  |  | FW16_GLEAN_10003484 | OXIDATION-REDUCTION | Alpha-hydroxy acid dehydrogenase, FMN-dependent |  |  |
| 110 | FW16_GLEAN_10003498 | 10 | 7 | 59 | 7 | 200.64 | 4.582367 | 93.99 | FW16_GLEAN_10003498 |  | FW16_GLEAN_10003498 |  | FW16_GLEAN_10003498 |  | FW16_GLEAN_10003498 |  | CARBOHYDRATE METABOLISM |  | GH3 | β-glucosidase /xylan 1,4-β-xylosidase |
| 111 | FW16_GLEAN_10003510 | 2 | 1 | 14 | 1 | 38.51 | 4.584323 | 48.76 |  |  |  |  |  |  | FW16_GLEAN_10003510 | FW16_GLEAN_10003510 | OTHER BIOLOGICAL PROCESS | tubulin beta |  |  |
| 112 | FW16_GLEAN_10003512 | 28 | 12 | 223 | 6 | 892.31 | 4.323031 | 83.20 | FW16_GLEAN_10003512 | FW16_GLEAN_10003512 | FW16_GLEAN_10003512 |  | FW16_GLEAN_10003512 | FW16_GLEAN_10003512 |  |  | CARBOHYDRATE METABOLISM |  | GH55 | exo-β-1,3-glucanase / endo-β-1,3-glucanase |
| 113 | FW16_GLEAN_10003540 | 1.08043 | 1 | 1 | 1 | 2.16024 | 9.717032 | 92.44 |  |  |  |  |  |  |  | FW16_GLEAN_10003540 | RNA METABOLIC | ATP-dependent RNA helicase |  |  |
| 114 | FW16_GLEAN_10003598 | 19 | 2 | 8 | 2 | 28.13 | 8.116592 | 13.11 | FW16_GLEAN_10003598 |  | FW16_GLEAN_10003598 | FW16_GLEAN_10003598 | FW16_GLEAN_10003598 |  | FW16_GLEAN_10003598 | FW16_GLEAN_10003598 | NONE CONSERVED DOMAIN |  |  |  |
| 115 | FW16_GLEAN_10003618 | 15.3846 | 3 | 3 | 3 | 8.89884 | 4.815724 | 26.83 | FW16_GLEAN_10003618 | FW16_GLEAN_10003618 | FW16_GLEAN_10003618 | FW16_GLEAN_10003618 | FW16_GLEAN_10003618 | FW16_GLEAN_10003618 |  |  | OTHER BIOLOGICAL PROCESS | triose-phosphate isomerase |  |  |
| 116 | FW16_GLEAN_10003683 | 0.660066 | 1 | 1 | 1 | 1.84292 | 4.760005 | 133.78 |  |  |  |  |  | FW16_GLEAN_10003683 |  |  | PROTEIN BIOSYNTHESIS | Splicing factor 3B subunit 3 |  |  |
| 117 | FW16_GLEAN_10003711 | 16.9772 | 8 | 8 | 8 | 18.1933 | 5.318553 | 55.04 | FW16_GLEAN_10003711 |  | FW16_GLEAN_10003711 |  |  |  |  |  | CARBOHYDRATE METABOLISM |  | GH1 | β-glucosidase |
| 118 | FW16_GLEAN_10003848 | 2.54453 | 1 | 1 | 1 | 2.88931 | 9.821688 | 44.59 |  |  |  | FW16_GLEAN_10003848 |  |  |  |  | OTHER BIOLOGICAL PROCESS | Protein kinase domain |  |  |
| 119 | FW16_GLEAN_10003883 | 1 | 1 | 1 | 1 | 2.02 | 5.427371 | 173.35 |  |  |  |  |  |  | FW16_GLEAN_10003883 | FW16_GLEAN_10003883 | NONE CONSERVED DOMAIN | hypothetical protein CEP54_011132 [Fusarium sp. AF-8] |  |  |
| 120 | FW16_GLEAN_10003884 | 6.25 | 1 | 1 | 1 | 3.46284 | 10.10933 | 24.79 |  |  | FW16_GLEAN_10003884 |  |  |  |  |  | PROTEIN BIOSYNTHESIS | 60S ribosomal protein |  |  |
| 121 | FW16_GLEAN_10003910 | 22 | 5 | 50 | 5 | 200.11 | 4.895213 | 58.47 |  |  |  |  |  |  | FW16_GLEAN_10003910 | FW16_GLEAN_10003910 | PROTEOLYSIS | CARBOXIPEPTIDASE Y |  |  |
| 122 | FW16_GLEAN_10003912 | 7.69231 | 1 | 1 | 1 | 2.98917 | 11.01438 | 17.87 |  |  |  |  |  |  | FW16_GLEAN_10003912 |  | PROTEIN BIOSYNTHESIS | Ribosomal protein S13 J |  |  |
| 123 | FW16_GLEAN_10003917 | 7 | 1 | 3 | 1 | 12.75 | 4.036835 | 34.91 |  |  |  |  |  |  |  | FW16_GLEAN_10003917 | CARBOHYDRATE METABOLISM |  | GH16 | mixed-linked glucanase |
| 124 | FW16_GLEAN_10003963 | 24.8148 | 4 | 6 | 3 | 18.5371 | 4.538169 | 30.05 | FW16_GLEAN_10003963 | FW16_GLEAN_10003963 | FW16_GLEAN_10003963 | FW16_GLEAN_10003963 | FW16_GLEAN_10003963 | FW16_GLEAN_10003963 |  |  | AMINO ACID METABOLISM | tyrosine 3-monooxygenase tryptophan 5-monooxygenase [Fusarium beomiforme] |  |  |
| 125 | FW16_GLEAN_10003969 | 9.06801 | 3 | 3 | 3 | 8.4814 | 4.771756 | 44.80 | FW16_GLEAN_10003969 |  | FW16_GLEAN_10003969 | FW16_GLEAN_10003969 |  |  |  |  | RNA METABOLIC | ATP-dependent RNA helicase |  |  |
| 126 | FW16_GLEAN_10004125 | 44 | 5 | 57 | 5 | 231.65 | 5.440212 | 20.14 |  |  |  |  |  |  |  | FW16_GLEAN_10004125 | OTHER BIOLOGICAL PROCESS | DUF1524 |  |  |
| 127 | FW16_GLEAN_10004130 | 12.5874 | 1 | 1 | 1 | 0 | 5.380604 | 16.88 | FW16_GLEAN_10004130 |  |  |  |  |  |  |  | OXIDATION-REDUCTION | Thioredoxin-like fold |  |  |
| 128 | FW16_GLEAN_10004137 | 6.55738 | 1 | 1 | 1 | 1.87277 | 10.11274 | 12.64 |  |  | FW16_GLEAN_10004137 |  |  |  |  |  | NONE CONSERVED DOMAIN |  |  |  |
| 129 | FW16_GLEAN_10004167 | 2.17391 | 1 | 1 | 1 | 2.93047 | 4.969946 | 55.77 |  |  | FW16_GLEAN_10004167 | FW16_GLEAN_10004167 |  |  |  |  | OTHER BIOLOGICAL PROCESS | 2,3-bisphosphoglycerate-independent phosphoglycerate mutase |  |  |
| 130 | FW16_GLEAN_10004183 | 6.27871 | 3 | 3 | 3 | 10.1624 | 8.133441 | 71.08 |  |  | FW16_GLEAN_10004183 |  |  | FW16_GLEAN_10004183 |  |  | OTHER BIOLOGICAL PROCESS | ATP citrate (pro-S)-lyase |  |  |
| 131 | FW16_GLEAN_10004184 | 5.11247 | 2 | 2 | 2 | 5.56992 | 5.183362 | 53.31 | FW16_GLEAN_10004184 | FW16_GLEAN_10004184 | FW16_GLEAN_10004184 | FW16_GLEAN_10004184 |  |  |  |  | OTHER BIOLOGICAL PROCESS | putative ATP-citrate synthase subunit 2 [Fusarium kuroshium] |  |  |
| 132 | FW16_GLEAN_10004186 | 5 | 2 | 13 | 2 | 37.21 | 4.194869 | 51.40 |  |  |  |  |  |  | FW16_GLEAN_10004186 | FW16_GLEAN_10004186 | CARBOHYDRATE METABOLISM |  | GH72 | β-1,3-glucanosyltransglycosylase |
| 133 | FW16_GLEAN_10004204 | 13.1455 | 2 | 2 | 2 | 7.38093 | 9.466052 | 23.75 |  |  | FW16_GLEAN_10004204 |  |  |  |  |  | PROTEIN BIOSYNTHESIS | small subunit ribosomal protein |  |  |
| 134 | FW16_GLEAN_10004226 | 2.12766 | 1 | 1 | 1 | 2.07835 | 5.538117 | 57.96 | FW16_GLEAN_10004226 |  |  |  |  |  |  |  | OTHER BIOLOGICAL PROCESS | related to UDP-galactopyranose mutase [Fusarium fujikuroi IMI 58289] |  |  |
| 135 | FW16_GLEAN_10004241 | 4.47761 | 2 | 2 | 2 | 3.62146 | 6.254163 | 50.71 | FW16_GLEAN_10004241 |  |  |  |  |  |  |  | OXIDATION-REDUCTION | glutathione reductase |  |  |
| 136 | FW16_GLEAN_10004296 | 4.62585 | 2 | 2 | 2 | 6.48958 | 5.340633 | 78.06 |  |  | FW16_GLEAN_10004296 | FW16_GLEAN_10004296 |  |  |  |  | OTHER BIOLOGICAL PROCESS | groEL, HSPD1 chaperonin GroEL |  |  |
| 137 | FW16_GLEAN_10004309 | 32.8402 | 7 | 9 | 7 | 27.2917 | 5.646375 | 36.06 | FW16_GLEAN_10004309 | FW16_GLEAN_10004309 | FW16_GLEAN_10004309 |  |  |  |  |  | NONE CONSERVED DOMAIN |  |  |  |
| 138 | FW16_GLEAN_10004314 | 15.9184 | 3 | 3 | 3 | 8.71807 | 6.072647 | 24.58 | FW16_GLEAN_10004314 | FW16_GLEAN_10004314 | FW16_GLEAN_10004314 | FW16_GLEAN_10004314 | FW16_GLEAN_10004314 | FW16_GLEAN_10004314 |  |  | AMINO ACID METABOLISM | Class I glutamine amidotransferase-like |  |  |
| 139 | FW16_GLEAN_10004324 | 5.85366 | 1 | 1 | 1 | 2.42139 | 4.562345 | 44.86 |  |  |  |  | FW16_GLEAN_10004324 |  |  |  | OXIDATION-REDUCTION | D-arabinitol dehydrogenase |  |  |
| 140 | FW16_GLEAN_10004332 | 56 | 26 | 428 | 26 | 1760.51 | 5.003079 | 66.73 | FW16_GLEAN_10004332 |  |  | FW16_GLEAN_10004332 |  | FW16_GLEAN_10004332 |  |  | CARBOHYDRATE METABOLISM |  | GH15-CBM20 | glucoamylase |
| 141 | FW16_GLEAN_10004386 | 1.61692 | 1 | 1 | 1 | 3.1296 | 6.073521 | 90.53 |  |  | FW16_GLEAN_10004386 |  |  |  |  |  | OXIDATION-REDUCTION | Xylulose 5-phosphate/Fructose 6-phosphate phosphoketolase |  |  |
| 142 | FW16_GLEAN_10004459 | 9.79021 | 4 | 4 | 4 | 12.0604 | 4.822618 | 63.12 |  |  | FW16_GLEAN_10004459 |  |  |  |  |  | OXIDATION-REDUCTION | pyruvate decarboxylase |  |  |
| 143 | FW16_GLEAN_10004469 | 4.52756 | 2 | 2 | 2 | 4.23318 | 6.509073 | 54.31 | FW16_GLEAN_10004469 |  | FW16_GLEAN_10004469 |  |  |  |  |  | LIPID METABOLIC | dihydrolipoyl dehydrogenase [Fusarium oxysporum f. sp. vasinfectum 25433] |  |  |
| 144 | FW16_GLEAN_10004486 | 6.56934 | 1 | 2 | 1 | 3.73971 | 10.57477 | 14.82 | FW16_GLEAN_10004486 | FW16_GLEAN_10004486 | FW16_GLEAN_10004486 | FW16_GLEAN_10004486 | FW16_GLEAN_10004486 | FW16_GLEAN_10004486 |  |  | OTHER BIOLOGICAL PROCESS | histone 2B [Nectria haematococca mpVI 77-13-4] |  |  |
| 145 | FW16_GLEAN_10004487 | 5.22388 | 1 | 1 | 1 | 2.4585 | 11.00569 | 14.15 |  | FW16_GLEAN_10004487 | FW16_GLEAN_10004487 |  |  |  |  |  | OTHER BIOLOGICAL PROCESS | Histone H2A |  |  |
| 146 | FW16_GLEAN_10004522 | 7.62463 | 2 | 2 | 2 | 6.10569 | 4.441572 | 34.57 | FW16_GLEAN_10004522 |  | FW16_GLEAN_10004522 |  |  | FW16_GLEAN_10004522 |  |  | CARBOHYDRATE METABOLISM |  | GH17 | glucan endo-1,3-β-glucosidase |
| 147 | FW16_GLEAN_10004549 | 0.790068 | 1 | 1 | 1 | 1.83516 | 5.761981 | 99.51 | FW16_GLEAN_10004549 |  |  |  |  |  |  |  | CARBOHYDRATE METABOLISM |  | GT20 | trehalose 6-phosphate synthase |
| 148 | FW16_GLEAN_10004573 | 38 | 10 | 225 | 10 | 946.85 | 6.477905 | 47.66 |  |  |  |  |  |  | FW16_GLEAN_10004573 | FW16_GLEAN_10004573 | CARBOHYDRATE METABOLISM |  | GH79 | β-glucuronidase |
| 149 | FW16_GLEAN_10004602 | 4.28571 | 1 | 3 | 1 | 11.3747 | 7.52212 | 36.58 | FW16_GLEAN_10004602 | FW16_GLEAN_10004602 | FW16_GLEAN_10004602 |  |  |  |  |  | CARBOHYDRATE METABOLISM |  | AA9-CBM1 | proteins are copper-dependent lytic polysaccharide monooxygenases |
| 150 | FW16_GLEAN_10004691 | 4 | 1 | 3 | 1 | 10.92 | 5.640548 | 44.94 |  |  |  |  |  |  | FW16_GLEAN_10004691 |  | CARBOHYDRATE METABOLISM |  | GH5 | exo-1,3-beta-glucanase or endo-1,6-β-glucosidase |
| 151 | FW16_GLEAN_10004719 | 4.69484 | 1 | 1 | 1 | 2.76552 | 3.43576 | 20.48 |  | FW16_GLEAN_10004719 |  |  |  |  |  |  | NONE CONSERVED DOMAIN |  |  |  |
| 152 | FW16_GLEAN_10004754 | 3 | 1 | 2 | 1 | 11.4 | 6.365185 | 58.35 |  |  |  |  |  |  | FW16_GLEAN_10004754 | FW16_GLEAN_10004754 | CARBOHYDRATE METABOLISM |  | AA7 | glucooligosaccharide oxidase / chitooligosaccharide oxidase |
| 153 | FW16_GLEAN_10004777 | 7 | 1 | 3 | 1 | 8.59 | 7.709103 | 28.86 |  |  |  |  |  |  | FW16_GLEAN_10004777 |  | CARBOHYDRATE METABOLISM |  | CE1 | xylan esterase |
| 154 | FW16_GLEAN_10004803 | 8 | 1 | 5 | 1 | 20.2 | 2.916027 | 32.57 |  |  |  |  |  |  | FW16_GLEAN_10004803 |  | NONE CONSERVED DOMAIN | hypothetical protein CEP53_002561 [Fusarium sp. AF-6] |  |  |
| 155 | FW16_GLEAN_10004829 | 2 | 1 | 7 | 1 | 27.12 | 3.622895 | 76.75 |  |  |  |  |  |  | FW16_GLEAN_10004829 | FW16_GLEAN_10004829 | NONE CONSERVED DOMAIN | hypothetical protein CDV31_016226 [Fusarium ambrosium] |  |  |
| 156 | FW16_GLEAN_10004843 | 23 | 13 | 45 | 13 | 167.7 | 4.946369 | 105.13 |  |  |  |  |  |  | FW16_GLEAN_10004843 | FW16_GLEAN_10004843 | CARBOHYDRATE METABOLISM |  | GH3 | β-glucosidase/ xylan 1,4-β-xylosidase |
| 157 | FW16_GLEAN_10004889 | 45 | 14 | 430 | 14 | 1935.42 | 4.357307 | 46.69 | FW16_GLEAN_10004889 | FW16_GLEAN_10004889 | FW16_GLEAN_10004889 | FW16_GLEAN_10004889 | FW16_GLEAN_10004889 | FW16_GLEAN_10004889 | FW16_GLEAN_10004889 | FW16_GLEAN_10004889 | CARBOHYDRATE METABOLISM |  | GH5-CBM1 | endo-β-1,4-glucanase / cellulase/other |
| 158 | FW16_GLEAN_10004932 | 32 | 12 | 137 | 4 | 579.06 | 4.944519 | 57.93 |  |  |  |  |  |  | FW16_GLEAN_10004932 | FW16_GLEAN_10004932 | LIPID METABOLIC |  |  |  |
| 159 | FW16_GLEAN_10004942 | 13 | 3 | 10 | 3 | 28.83 | 5.459106 | 37.29 | FW16_GLEAN_10004942 |  |  |  |  |  | FW16_GLEAN_10004942 | FW16_GLEAN_10004942 | AMINO ACID METABOLISM | L-asparaginase, type II |  |  |
| 160 | FW16_GLEAN_10005065 | 1.39104 | 1 | 1 | 1 | 1.68296 | 6.62922 | 74.03 |  | FW16_GLEAN_10005065 |  |  |  |  |  |  | NONE CONSERVED DOMAIN |  |  |  |
| 161 | FW16_GLEAN_10005084 | 3 | 1 | 6 | 1 | 7.38 | 4.499718 | 30.22 |  |  | FW16_GLEAN_10005084 |  |  |  |  |  | NONE CONSERVED DOMAIN |  |  |  |
| 162 | FW16_GLEAN_10005091 | 2 | 1 | 2 | 1 | 4.34 | 5.132512 | 45.47 |  |  |  |  |  |  | FW16_GLEAN_10005091 | FW16_GLEAN_10005091 | CARBOHYDRATE METABOLISM |  | GH28 | galacturan 1,4-α-galacturonidase |
| 163 | FW16_GLEAN_10005103 | 13 | 2 | 7 | 2 | 23.04 | 3.840057 | 21.82 |  |  |  |  |  |  |  | FW16_GLEAN_10005103 | NONE CONSERVED DOMAIN |  |  |  |
| 164 | FW16_GLEAN_10005174 | 8.48214 | 1 | 1 | 1 | 3.70215 | 4.534456 | 24.25 |  | FW16_GLEAN_10005174 |  |  |  |  |  |  | NONE CONSERVED DOMAIN |  |  |  |
| 165 | FW16_GLEAN_10005240 | 32 | 9 | 392 | 9 | 1906.08 | 6.06625 | 40.84 |  |  |  |  |  |  | FW16_GLEAN_10005240 | FW16_GLEAN_10005240 | PROTEOLYSIS | PEPTIDASE S8 FAMILY |  |  |
| 166 | FW16_GLEAN_10005257 | 4.91071 | 1 | 1 | 1 | 1.91084 | 3.977226 | 22.30 |  | FW16_GLEAN_10005257 | FW16_GLEAN_10005257 | FW16_GLEAN_10005257 | FW16_GLEAN_10005257 | FW16_GLEAN_10005257 |  |  | NONE CONSERVED DOMAIN |  |  |  |
| 167 | FW16_GLEAN_10005278 | 26.1111 | 4 | 4 | 4 | 12.556 | 6.350357 | 19.48 | FW16_GLEAN_10005278 | FW16_GLEAN_10005278 | FW16_GLEAN_10005278 | FW16_GLEAN_10005278 | FW16_GLEAN_10005278 | FW16_GLEAN_10005278 |  |  | OXIDATION-REDUCTION | Peptidyl-prolyl cis-trans isomerase, mitochondrial [Fusarium ambrosium] |  |  |
| 168 | FW16_GLEAN_10005371 | 11.1111 | 1 | 1 | 1 | 0 | 4.288388 | 29.67 |  |  |  |  |  |  | FW16_GLEAN_10005371 | FW16_GLEAN_10005371 | NONE CONSERVED DOMAIN | hypothetical protein CDV36_007420 [Fusarium kuroshium] |  |  |
| 169 | FW16_GLEAN_10005411 | 7.02811 | 2 | 2 | 2 | 7.13322 | 5.168931 | 54.15 | FW16_GLEAN_10005411 |  | FW16_GLEAN_10005411 |  |  |  |  |  | OTHER BIOLOGICAL PROCESS | Pleckstrin homology domain |  |  |
| 170 | FW16_GLEAN_10005551 | 19 | 7 | 51 | 7 | 208.86 | 8.510742 | 55.23 | FW16_GLEAN_10005551 | FW16_GLEAN_10005551 | FW16_GLEAN_10005551 | FW16_GLEAN_10005551 | FW16_GLEAN_10005551 | FW16_GLEAN_10005551 | FW16_GLEAN_10005551 | FW16_GLEAN_10005551 | OTHER BIOLOGICAL PROCESS | PENTA MXKDX SUPERFAMILY |  |  |
| 171 | FW16_GLEAN_10005559 | 7.28155 | 1 | 1 | 1 | 2.94954 | 11.06557 | 23.19 |  |  |  |  | FW16_GLEAN_10005559 |  |  |  | PROTEIN BIOSYNTHESIS | 40S ribosomal protein S8 |  |  |
| 172 | FW16_GLEAN_10005570 | 4.53515 | 1 | 1 | 1 | 0 | 4.525009 | 41.12 |  |  |  | FW16_GLEAN_10005570 |  |  |  |  | CARBOHYDRATE METABOLISM |  | AA11 | proteins are copper-dependent lytic polysaccharide monooxygenases |
| 173 | FW16_GLEAN_10005596 | 5.03817 | 3 | 43 | 3 | 177.62 | 4.773026 | 71.33 | FW16_GLEAN_10005596 | FW16_GLEAN_10005596 | FW16_GLEAN_10005596 | FW16_GLEAN_10005596 | FW16_GLEAN_10005596 | FW16_GLEAN_10005596 |  |  | NONE CONSERVED DOMAIN |  |  |  |
| 174 | FW16_GLEAN_10005619 | 0.853659 | 1 | 1 | 1 | 0 | 5.291055 | 93.65 |  |  |  |  | FW16_GLEAN_10005619 | FW16_GLEAN_10005619 |  | FW16_GLEAN_10005619 | OTHER BIOLOGICAL PROCESS | ATPase subunit a |  |  |
| 175 | FW16_GLEAN_10005637 | 4.8433 | 1 | 1 | 1 | 2.36277 | 6.731316 | 34.97 | FW16_GLEAN_10005637 |  |  |  |  | FW16_GLEAN_10005637 |  |  | CARBOHYDRATE METABOLISM |  | GH128 | β-1,3-glucanase |
| 176 | FW16_GLEAN_10005679 | 13 | 4 | 20 | 4 | 64.71 | 4.36571 | 33.11 |  |  |  |  |  | FW16_GLEAN_10005679 |  |  | NONE CONSERVED DOMAIN | hypothetical protein CEP54_006534 [Fusarium sp. AF-8] |  |  |
| 177 | FW16_GLEAN_10005727 | 2.89331 | 1 | 1 | 1 | 3.78572 | 5.004976 | 59.82 |  |  | FW16_GLEAN_10005727 |  |  | FW16_GLEAN_10005727 |  |  | OXIDATION-REDUCTION | Alpha-D-phosphohexomutase |  |  |
| 178 | FW16_GLEAN_10005737 | 10.5263 | 1 | 1 | 1 | 4.27627 | 10.49895 | 17.46 |  |  | FW16_GLEAN_10005737 |  |  |  |  |  | PROTEIN BIOSYNTHESIS | 40S ribosomal protein S15 |  |  |
| 179 | FW16_GLEAN_10005759 | 25 | 4 | 78 | 4 | 266.41 | 4.525208 | 26.50 | FW16_GLEAN_10005759 |  | FW16_GLEAN_10005759 |  |  |  | FW16_GLEAN_10005759 | FW16_GLEAN_10005759 | OTHER BIOLOGICAL PROCESS | GPI-ANCHORED/Kre9/Knh1 |  |  |
| 180 | FW16_GLEAN_10005843 | 4.48276 | 1 | 1 | 1 | 3.00604 | 4.908931 | 32.69 |  |  | FW16_GLEAN_10005843 |  |  |  |  |  | OTHER BIOLOGICAL PROCESS | inorganic pyrophosphatase |  |  |
| 181 | FW16_GLEAN_10005867 | 26 | 4 | 174 | 1 | 669.48 | 4.280581 | 23.07 |  | FW16_GLEAN_10005867 | FW16_GLEAN_10005867 | FW16_GLEAN_10005867 | FW16_GLEAN_10005867 | FW16_GLEAN_10005867 |  |  | NONE CONSERVED DOMAIN |  |  |  |
| 182 | FW16_GLEAN_10005868 | 5.05837 | 1 | 1 | 1 | 4.33731 | 6.389336 | 57.25 |  |  | FW16_GLEAN_10005868 |  |  |  |  |  | RNA METABOLIC | UTP:glucose-1-phosphate uridylyltransferase |  |  |
| 183 | FW16_GLEAN_10005875 | 14 | 7 | 39 | 7 | 124.61 | 4.549232 | 57.58 |  |  |  |  |  |  | FW16_GLEAN_10005875 | FW16_GLEAN_10005875 | OTHER BIOLOGICAL PROCESS | HISTIDINE PHOSPHATASE |  |  |
| 184 | FW16_GLEAN_10005918 | 67 | 41 | 9490 | 32 | 27924.5 | 4.603791 | 52.92 | FW16_GLEAN_10005918 | FW16_GLEAN_10005918 | FW16_GLEAN_10005918 | FW16_GLEAN_10005918 | FW16_GLEAN_10005918 | FW16_GLEAN_10005918 | FW16_GLEAN_10005918 | FW16_GLEAN_10005918 | CARBOHYDRATE METABOLISM |  | GH7-CBM1 | endo-β-1,4-glucanase |
| 185 | FW16_GLEAN_10005936 | 38 | 5 | 44 | 5 | 154.51 | 3.877474 | 16.03 |  |  |  |  |  |  | FW16_GLEAN_10005936 | FW16_GLEAN_10005936 | OTHER BIOLOGICAL PROCESS | CFEM DOMAIN (RELATED TO FUNGAL PHATOGENESIS |  |  |
| 186 | FW16_GLEAN_10005937 | 4 | 1 | 1 | 1 | 1.62953 | 4.768452 | 27.28 |  |  |  | FW16_GLEAN_10005937 |  |  |  |  | OTHER BIOLOGICAL PROCESS | ribulose-phosphate 3-epimerase |  |  |
| 187 | FW16_GLEAN_10005957 | 20.8 | 2 | 2 | 2 | 3.74537 | 4.947302 | 13.55 | FW16_GLEAN_10005957 |  | FW16_GLEAN_10005957 | FW16_GLEAN_10005957 |  |  |  |  | AMINO ACID METABOLISM | 2-iminobutanoate/2-iminopropanoate deaminase |  |  |
| 188 | FW16_GLEAN_10005976 | 7 | 2 | 7 | 2 | 24.8 | 5.497471 | 47.30 |  |  |  |  |  |  |  | FW16_GLEAN_10005976 | OTHER BIOLOGICAL PROCESS | DUF4243 (DOMAIN OF UNKNOW FUNCTION) |  |  |
| 189 | FW16_GLEAN_10005996 | 4.28571 | 1 | 2 | 1 | 5.48817 | 6.854277 | 31.31 | FW16_GLEAN_10005996 |  | FW16_GLEAN_10005996 |  | FW16_GLEAN_10005996 | FW16_GLEAN_10005996 |  |  | PROTEOLYSIS | proline iminopeptidase |  |  |
| 190 | FW16_GLEAN_10006005 | 2.88462 | 1 | 1 | 1 | 2.31409 | 5.659318 | 29.55 | FW16_GLEAN_10006005 | FW16_GLEAN_10006005 |  |  |  |  |  |  | CARBOHYDRATE METABOLISM |  | GH7 | endo-β-1,4-glucanase |
| 191 | FW16_GLEAN_10006013 | 11.7391 | 2 | 2 | 2 | 4.6297 | 4.07776 | 24.99 | FW16_GLEAN_10006013 |  | FW16_GLEAN_10006013 | FW16_GLEAN_10006013 |  | FW16_GLEAN_10006103 |  |  | OTHER BIOLOGICAL PROCESS | Glutathione S-transferase |  |  |
| 192 | FW16_GLEAN_10006014 | 2.82776 | 1 | 1 | 1 | 2.83222 | 4.638529 | 41.70 |  |  | FW16_GLEAN_10006014 |  |  |  |  |  | PROTEOLYSIS | 26S proteasome |  |  |
| 193 | FW16_GLEAN_10006087 | 8.09249 | 1 | 1 | 1 | 3.61278 | 10.68411 | 19.89 |  |  | FW16_GLEAN_10006087 |  |  |  |  |  | PROTEIN BIOSYNTHESIS | 60S ribosomal protein |  |  |
| 194 | FW16_GLEAN_10006088 | 9.60145 | 4 | 4 | 4 | 12.7502 | 9.435578 | 59.66 | FW16_GLEAN_10006088 |  | FW16_GLEAN_10006088 | FW16_GLEAN_10006088 |  |  |  |  | OTHER BIOLOGICAL PROCESS | ATP1 F-type H+-transporting ATPase |  |  |
| 195 | FW16_GLEAN_10006099 | 6.01852 | 1 | 1 | 1 | 3.0361 | 7.158691 | 24.48 | FW16_GLEAN_10006099 |  |  | FW16_GLEAN_10006099 |  |  |  |  | OTHER BIOLOGICAL PROCESS | Small GTPase superfamily |  |  |
| 196 | FW16_GLEAN_10006103 | 1.99715 | 1 | 1 | 1 | 4.00863 | 5.815871 | 78.24 |  |  | FW16_GLEAN_10006103 |  | FW16_GLEAN_10006103 |  |  |  | PROTEOLYSIS | Dipeptidyl-peptidase 5 |  |  |
| 197 | FW16_GLEAN_10006117 | 12.8049 | 4 | 4 | 4 | 15.7927 | 5.818914 | 54.13 |  |  | FW16_GLEAN_10006117 | FW16_GLEAN_10006117 | FW16_GLEAN_10006117 | FW16_GLEAN_10006117 |  |  | OXIDATION-REDUCTION | 6-phosphogluconate dehydrogenase |  |  |
| 198 | FW16_GLEAN_10006185 | 20 | 3 | 18 | 3 | 74.3 | 3.439517 | 19.57 |  | FW16_GLEAN_10006185 |  |  |  |  | FW16_GLEAN_10006185 | FW16_GLEAN_10006185 | NONE CONSERVED DOMAIN |  |  |  |
| 199 | FW16_GLEAN_10006190 | 1.32248 | 1 | 1 | 1 | 2.65243 | 8.088921 | 110.95 |  |  | FW16_GLEAN_10006190 |  |  |  |  |  | RNA METABOLIC | RRP44 exosome complex exonuclease DIS3 |  |  |
| 200 | FW16_GLEAN_10006258 | 1.68955 | 1 | 1 | 1 | 0 | 5.718728 | 106.69 | FW16_GLEAN_10006258 |  |  |  |  |  |  |  | OTHER BIOLOGICAL PROCESS | Nucleic acid-binding |  |  |
| 201 | FW16_GLEAN_10006264 | 3.95833 | 2 | 2 | 2 | 3.00438 | 4.397281 | 50.53 | FW16_GLEAN_10006264 |  | FW16_GLEAN_10006264 |  | FW16_GLEAN_10006264 | FW16_GLEAN_10006264 |  |  | OXIDATION-REDUCTION | Protein disulfide-isomerase [Fusarium kuroshium] |  |  |
| 202 | FW16_GLEAN_10006270 | 26.1589 | 5 | 70 | 4 | 172.97 | 4.815811 | 33.47 | FW16_GLEAN_10006270 | FW16_GLEAN_10006270 | FW16_GLEAN_10006270 | FW16_GLEAN_10006270 | FW16_GLEAN_10006270 | FW16_GLEAN_10006270 | FW16_GLEAN_10006270 | FW16_GLEAN_10006270 | OTHER BIOLOGICAL PROCESS | PHOSPHOSERINE/TREONINE BINDING PROTEIN |  |  |
| 203 | FW16_GLEAN_10006272 | 31 | 5 | 48 | 5 | 165.85 | 4.682882 | 16.55 |  |  |  |  |  |  | FW16_GLEAN_10006272 | FW16_GLEAN_10006272 | NONE CONSERVED DOMAIN |  |  |  |
| 204 | FW16_GLEAN_10006290 | 4.44444 | 2 | 2 | 2 | 5.36779 | 6.619897 | 57.03 |  |  | FW16_GLEAN_10006290 |  |  |  |  |  | OXIDATION-REDUCTION | glucose-6-phosphate 1-dehydrogenase |  |  |
| 205 | FW16_GLEAN_10006294 | 21 | 9 | 58 | 9 | 210.14 | 4.948858 | 65.70 |  |  |  |  |  |  | FW16_GLEAN_10006294 | FW16_GLEAN_10006294 | CARBOHYDRATE METABOLISM |  | GH20 | β-hexosaminidase |
| 206 | FW16_GLEAN_10006308 | 11.3527 | 3 | 3 | 3 | 10.8875 | 5.250278 | 44.81 |  |  | FW16_GLEAN_10006308 |  | FW16_GLEAN_10006308 |  |  |  | OTHER BIOLOGICAL PROCESS | acetate kinase |  |  |
| 207 | FW16_GLEAN_10006361 | 44.5205 | 13 | 18 | 13 | 66.7532 | 4.673237 | 47.14 | FW16_GLEAN_10006361 | FW16_GLEAN_10006361 | FW16_GLEAN_10006361 | FW16_GLEAN_10006361 | FW16_GLEAN_10006361 | FW16_GLEAN_10006361 |  |  | OXIDATION-REDUCTION | eno enolase |  |  |
| 208 | FW16_GLEAN_10006366 | 4.11899 | 1 | 1 | 1 | 3.9038 | 4.989795 | 43.97 | FW16_GLEAN_10006366 | FW16_GLEAN_10006366 |  |  | FW16_GLEAN_10006366 |  |  |  | CARBOHYDRATE METABOLISM |  | GH132 | beta-glucosidase β-1,3-glucan |
| 209 | FW16_GLEAN_10006422 | 1 | 1 | 8 | 1 | 30.44 | 5.395332 | 115.59 |  |  | FW16_GLEAN_10006422 |  |  |  |  |  | OTHER BIOLOGICAL PROCESS | ABC transporter G family member ARB_01379 |  |  |
| 210 | FW16_GLEAN_10006436 | 6.8 | 1 | 2 | 1 | 6.4 | 5.562231 | 26.66 |  |  | FW16_GLEAN_10006436 |  |  |  |  |  | CARBOHYDRATE METABOLISM |  | AA6 | 1,4-benzoquinone reductase |
| 211 | FW16_GLEAN_10006441 | 8.33333 | 1 | 1 | 1 | 3.11758 | 4.38448 | 18.50 |  |  | FW16_GLEAN_10006441 |  |  |  |  |  | OTHER BIOLOGICAL PROCESS | peptidyl-prolyl cis-trans isomerase , chaperones |  |  |
| 212 | FW16_GLEAN_10006458 | 8.29787 | 3 | 3 | 3 | 8.97033 | 8.224775 | 51.99 | FW16_GLEAN_10006458 | FW16_GLEAN_10006458 | FW16_GLEAN_10006458 |  |  | FW16_GLEAN_10006458 |  |  | OTHER BIOLOGICAL PROCESS | Citrate synthase C |  |  |
| 213 | FW16_GLEAN_10006460 | 8.44156 | 5 | 5 | 5 | 17.8581 | 4.714627 | 100.14 |  |  | FW16_GLEAN_10006460 |  |  |  |  |  | OTHER BIOLOGICAL PROCESS | H+-transporting ATPase |  |  |
| 214 | FW16_GLEAN_10006519 | 7.36434 | 1 | 1 | 1 | 3.11309 | 10.71265 | 28.09 |  |  | FW16_GLEAN_10006519 |  |  |  |  |  | PROTEIN BIOSYNTHESIS | 40S ribosomal protein S2 |  |  |
| 215 | FW16_GLEAN_10006551 | 5 | 1 | 3 | 1 | 8.01 | 5.197724 | 37.03 |  |  | FW16_GLEAN_10006551 |  |  |  |  |  | OTHER BIOLOGICAL PROCESS | NAD(P)H-dependent D-xylose reductase |  |  |
| 216 | FW16_GLEAN_10006564 | 6.38978 | 1 | 1 | 1 | 4.25064 | 4.613889 | 34.02 |  |  | FW16_GLEAN_10006564 |  |  | FW16_GLEAN_10006564 |  |  | OXIDATION-REDUCTION | glucose-6-phosphate 1-epimerase |  |  |
| 217 | FW16_GLEAN_10006630 | 2 | 1 | 18 | 1 | 81.07 | 3.904092 | 86.12 |  |  | FW16_GLEAN_10006630 |  |  |  |  |  | NONE CONSERVED DOMAIN | hypothetical protein BHE90_013809 [Fusarium euwallaceae] |  |  |
| 218 | FW16_GLEAN_10006647 | 42 | 16 | 254 | 16 | 992.72 | 4.49191 | 61.11 | FW16_GLEAN_10006647 | FW16_GLEAN_10006647 | FW16_GLEAN_10006647 | FW16_GLEAN_10006647 |  | FW16_GLEAN_10006647 | FW16_GLEAN_10006647 | FW16_GLEAN_10006647 | LIPID METABOLIC |  |  |  |
| 219 | FW16_GLEAN_10006648 | 6.75991 | 5 | 5 | 5 | 13.473 | 5.055851 | 96.09 | FW16_GLEAN_10006648 | FW16_GLEAN_10006648 | FW16_GLEAN_10006648 | FW16_GLEAN_10006648 | FW16_GLEAN_10006648 | FW16_GLEAN_10006648 |  |  | PROTEOLYSIS | Peptidase M1 |  |  |
| 220 | FW16_GLEAN_10006674 | 5.57769 | 1 | 1 | 1 | 3.1363 | 9.153913 | 26.23 | FW16_GLEAN_10006674 |  |  |  |  |  |  |  | NONE CONSERVED DOMAIN |  |  |  |
| 221 | FW16_GLEAN_10006714 | 4.8951 | 1 | 1 | 1 | 2.4585 | 10.75973 | 15.25 |  | FW16_GLEAN_10006714 | FW16_GLEAN_10006714 |  |  |  |  |  | OTHER BIOLOGICAL PROCESS | Histone H2A |  |  |
| 222 | FW16_GLEAN_10006734 | 15 | 7 | 30 | 7 | 98.31 | 4.685306 | 68.32 |  |  |  |  |  |  | FW16_GLEAN_10006734 |  | CARBOHYDRATE METABOLISM |  | GH1 | β-glucosidase / β-galactosidase / β-mannosidase/ other |
| 223 | FW16_GLEAN_10006738 | 11.0048 | 1 | 1 | 1 | 3.09731 | 4.639664 | 22.28 |  |  |  | FW16_GLEAN_10006738 |  |  |  |  | NONE CONSERVED DOMAIN |  |  |  |
| 224 | FW16_GLEAN_10006784 | 18 | 6 | 128 | 6 | 524.2 | 5.478546 | 62.59 |  |  | FW16_GLEAN_10006784 |  | FW16_GLEAN_10006784 | FW16_GLEAN_10006784 | FW16_GLEAN_10006784 | FW16_GLEAN_10006784 | PROTEOLYSIS | CLN2 tripeptidyl-peptidase I |  |  |
| 225 | FW16_GLEAN_10006822 | 32 | 10 | 140 | 5 | 549.27 | 5.77758 | 57.04 | FW16_GLEAN_10006822 | FW16_GLEAN_10006822 | FW16_GLEAN_10006822 | FW16_GLEAN_10006822 | FW16_GLEAN_10006822 | FW16_GLEAN_10006822 |  |  | CARBOHYDRATE METABOLISM |  | GH43 | β-xylosidase / α-L-arabinofuranosidase / xylanase |
| 226 | FW16_GLEAN_10006835 | 54 | 14 | 513 | 14 | 2215.18 | 5.188786 | 45.76 | FW16_GLEAN_10006835 | FW16_GLEAN_10006835 | FW16_GLEAN_10006835 | FW16_GLEAN_10006835 |  | FW16_GLEAN_10006835 | FW16_GLEAN_10006835 | FW16_GLEAN_10006835 | CARBOHYDRATE METABOLISM |  | GH6-CBM1 | endoglucanase / cellobiohydrolase |
| 227 | FW16_GLEAN_10006900 | 9.13043 | 2 | 2 | 2 | 3.38626 | 5.727097 | 22.20 |  |  |  | FW16_GLEAN_10006900 | FW16_GLEAN_10006900 |  |  |  | CARBOHYDRATE METABOLISM |  | CE5 | acetyl xylan esterase / cutinase |
| 228 | FW16_GLEAN_10006970 | 22 | 11 | 204 | 10 | 737.56 | 4.982499 | 69.89 |  |  |  |  |  |  | FW16_GLEAN_10006970 | FW16_GLEAN_10006970 | PROTEOLYSIS | PEPSIN |  |  |
| 229 | FW16_GLEAN_10007028 | 5 | 1 | 5 | 1 | 17.41 | 4.662203 | 32.49 |  |  | FW16_GLEAN_10007028 |  |  |  |  |  | LIPID METABOLIC | triacylglycerol lipase |  |  |
| 230 | FW16_GLEAN_10007037 | 41.866 | 11 | 16 | 11 | 48.3695 | 5.220933 | 44.62 | FW16_GLEAN_10007037 | FW16_GLEAN_10007037 | FW16_GLEAN_10007037 | FW16_GLEAN_10007037 | FW16_GLEAN_10007037 | FW16_GLEAN_10007037 |  |  | OXIDATION-REDUCTION | phosphoglycerate kinase |  |  |
| 231 | FW16_GLEAN_10007082 | 20 | 8 | 31 | 8 | 116.93 | 6.184772 | 61.18 | FW16_GLEAN_10007082 |  | FW16_GLEAN_10007082 |  |  |  | FW16_GLEAN_10007082 | FW16_GLEAN_10007082 | AMINO ACID METABOLISM | amiE amidase |  |  |
| 232 | FW16_GLEAN_10007085 | 22 | 6 | 60 | 6 | 242 | 6.043246 | 47.20 | FW16_GLEAN_10007085 | FW16_GLEAN_10007085 | FW16_GLEAN_10007085 |  |  | FW16_GLEAN_10007085 | FW16_GLEAN_10007085 | FW16_GLEAN_10007085 | CARBOHYDRATE METABOLISM |  | GH7 | endo-β-1,4-glucanase |
| 233 | FW16_GLEAN_10007143 | 9 | 1 | 2 | 1 | 6.98 | 8.265688 | 21.87 |  |  | FW16_GLEAN_10007143 |  |  |  |  |  | CARBOHYDRATE METABOLISM |  | CBM63 | bind cellulose |
| 234 | FW16_GLEAN_10007169 | 15 | 3 | 25 | 3 | 99.26 | 3.796479 | 30.27 |  |  |  |  |  |  | FW16_GLEAN_10007169 |  | CARBOHYDRATE METABOLISM |  | CE5 | acetyl xylan esterase / cutinase |
| 235 | FW16_GLEAN_10007175 | 9 | 2 | 2 | 2 | 6.46 | 6.677584 | 45.01 |  |  |  |  |  |  | FW16_GLEAN_10007175 |  | CARBOHYDRATE METABOLISM |  | GH43 | β-xylosidase / α-L-arabinofuranosidase / xylanase |
| 236 | FW16_GLEAN_10007217 | 25 | 6 | 10 | 6 | 31.2992 | 5.052559 | 39.15 | FW16_GLEAN_10007217 | FW16_GLEAN_10007217 | FW16_GLEAN_10007217 | FW16_GLEAN_10007217 | FW16_GLEAN_10007217 | FW16_GLEAN_10007217 |  |  | NONE CONSERVED DOMAIN |  |  |  |
| 237 | FW16_GLEAN_10007224 | 8.58209 | 1 | 1 | 1 | 0 | 8.454258 | 30.41 |  |  | FW16_GLEAN_10007224 |  |  |  |  |  | NONE CONSERVED DOMAIN | hypothetical protein CEP54_002781 [Fusarium sp. AF-8] |  |  |
| 238 | FW16_GLEAN_10007235 | 1.2987 | 1 | 1 | 1 | 2.19975 | 6.612223 | 88.07 |  |  |  | FW16_GLEAN_10007235 |  |  |  |  | RNA METABOLIC | TRL1 tRNA ligase |  |  |
| 239 | FW16_GLEAN_10007311 | 17.7258 | 3 | 4 | 3 | 13.725 | 4.488275 | 29.72 | FW16_GLEAN_10007311 | FW16_GLEAN_10007311 | FW16_GLEAN_10007311 | FW16_GLEAN_10007311 | FW16_GLEAN_10007311 | FW16_GLEAN_10007311 |  |  | CARBOHYDRATE METABOLISM |  | GH75 | endochitosanase |
| 240 | FW16_GLEAN_10007315 | 4.86726 | 1 | 1 | 1 | 2.03332 | 9.413151 | 24.81 |  |  |  | FW16_GLEAN_10007315 |  |  |  |  | OXIDATION-REDUCTION | nucleoside-diphosphate-sugar epimerase [Fusarium longipes] |  |  |
| 241 | FW16_GLEAN_10007320 | 3.31325 | 1 | 1 | 1 | 1.77144 | 6.009431 | 36.81 | FW16_GLEAN_10007320 |  |  |  |  |  |  |  | OXIDATION-REDUCTION | L-glyceraldehyde reductase |  |  |
| 242 | FW16_GLEAN_10007334 | 41.7112 | 6 | 32 | 6 | 112.12 | 9.285429 | 18.97 | FW16_GLEAN_10007334 | FW16_GLEAN_10007334 | FW16_GLEAN_10007334 | FW16_GLEAN_10007334 | FW16_GLEAN_10007334 | FW16_GLEAN_10007334 | FW16_GLEAN_10007334 | FW16_GLEAN_10007334 | OTHER BIOLOGICAL PROCESS | alta1 |  |  |
| 243 | FW16_GLEAN_10007390 | 10.453 | 3 | 3 | 3 | 4.41431 | 4.361991 | 27.56 |  |  | FW16_GLEAN_10007390 | FW16_GLEAN_10007390 | FW16_GLEAN_10007390 | FW16_GLEAN_10007390 |  |  | NONE CONSERVED DOMAIN |  |  |  |
| 244 | FW16_GLEAN_10007414 | 5.28846 | 1 | 1 | 1 | 2.51254 | 6.924026 | 23.21 | FW16_GLEAN_10007414 |  |  | FW16_GLEAN_10007414 |  |  |  |  | OTHER BIOLOGICAL PROCESS | Small GTPase superfamily |  |  |
| 245 | FW16_GLEAN_10007422 | 2.52101 | 1 | 2 | 1 | 2.42569 | 4.764844 | 104.57 |  |  |  | FW16_GLEAN_10007422 |  |  |  |  | OTHER BIOLOGICAL PROCESS | DNA replication licensing factor MCM6 |  |  |
| 246 | FW16_GLEAN_10007429 | 24 | 9 | 89 | 9 | 334.29 | 4.580511 | 63.89 |  |  |  |  |  |  |  | FW16_GLEAN_10007429 | PROTEOLYSIS | PEPTIDASE |  |  |
| 247 | FW16_GLEAN_10007434 | 8.30769 | 2 | 2 | 2 | 6.55653 | 7.211754 | 34.15 |  |  | FW16_GLEAN_10007434 |  |  |  |  |  | OXIDATION-REDUCTION | MDH2 malate dehydrogenase |  |  |
| 248 | FW16_GLEAN_10007455 | 9 | 1 | 7 | 1 | 25.99 | 4.380178 | 17.51 |  |  | FW16_GLEAN_10007455 |  |  |  |  |  | PROTEOLYSIS | lysozyme |  |  |
| 249 | FW16_GLEAN_10007468 | 3 | 1 | 1 | 1 | 3.16 | 4.46819 | 61.08 |  |  | FW16_GLEAN_10007468 |  |  |  | FW16_GLEAN_10007468 | FW16_GLEAN_10007468 | NONE CONSERVED DOMAIN | hypothetical protein CDV31_007427 [Fusarium ambrosium] |  |  |
| 250 | FW16_GLEAN_10007489 | 1.73502 | 1 | 1 | 1 | 0 | 9.680714 | 71.57 | FW16_GLEAN_10007489 |  |  |  |  |  |  |  | NONE CONSERVED DOMAIN |  |  |  |
| 251 | FW16_GLEAN_10007529 | 5 | 1 | 3 | 1 | 8.98 | 10.09318 | 26.17 | FW16_GLEAN_10007529 | FW16_GLEAN_10007529 | FW16_GLEAN_10007529 |  |  |  |  |  | CARBOHYDRATE METABOLISM |  | GH24 | lysozyme |
| 252 | FW16_GLEAN_10007555 | 3 | 1 | 3 | 1 | 9.89 | 5.872237 | 46.90 |  |  | FW16_GLEAN_10007555 |  |  |  |  |  | NONE CONSERVED DOMAIN | hypothetical protein CDV31_003683 [Fusarium ambrosium] |  |  |
| 253 | FW16_GLEAN_10007569 | 2 | 1 | 2 | 1 | 4.63 | 4.054325 | 51.68 |  |  | FW16_GLEAN_10007569 |  |  |  |  |  | CARBOHYDRATE METABOLISM |  | GH16-CBM18 | Chitinase |
| 254 | FW16_GLEAN_10007573 | 4.09207 | 1 | 2 | 1 | 4.16 | 6.608156 | 40.06 | FW16_GLEAN_10007573 | FW16_GLEAN_10007573 | FW16_GLEAN_10007573 |  |  |  |  |  | NONE CONSERVED DOMAIN |  |  |  |
| 255 | FW16_GLEAN_10007596 | 2.3976 | 1 | 1 | 1 | 3.07422 | 9.253889 | 108.60 |  |  | FW16_GLEAN_10007596 |  |  |  |  |  | OTHER BIOLOGICAL PROCESS | Arrestin C-terminal-like domain |  |  |
| 256 | FW16_GLEAN_10007629 | 18 | 5 | 67 | 2 | 216.47 | 5.56026 | 41.69 |  |  |  |  |  |  | FW16_GLEAN_10007629 | FW16_GLEAN_10007629 | PROTEOLYSIS | SERINE CARBOXIPEPTIDASE |  |  |
| 257 | FW16_GLEAN_10007711 | 38 | 5 | 18 | 5 | 74.82 | 4.735003 | 24.91 | FW16_GLEAN_10007711 | FW16_GLEAN_10007711 | FW16_GLEAN_10007711 | FW16_GLEAN_10007711 | FW16_GLEAN_10007711 | FW16_GLEAN_10007711 |  | FW16_GLEAN_10007711 | CARBOHYDRATE METABOLISM |  | AA9 | proteins are copper-dependent lytic polysaccharide monooxygenases |
| 258 | FW16_GLEAN_10007759 | 8.66667 | 1 | 1 | 1 | 2.85311 | 11.14931 | 15.95 |  |  | FW16_GLEAN_10007759 |  |  |  |  |  | PROTEIN BIOSYNTHESIS | 40S ribosomal protein |  |  |
| 259 | FW16_GLEAN_10007768 | 2.04513 | 1 | 1 | 1 | 0 | 5.91778 | 157.63 | FW16_GLEAN_10007768 |  |  |  |  |  |  |  | RNA METABOLIC | RNA polymerase II transcription factor |  |  |
| 260 | FW16_GLEAN_10007776 | 21.7647 | 3 | 3 | 3 | 6.1612 | 4.295539 | 18.90 | FW16_GLEAN_10007776 |  |  | FW16_GLEAN_10007776 |  |  |  |  | OTHER BIOLOGICAL PROCESS | Mss4/translationally controlled tumour-associated TCTP |  |  |
| 261 | FW16_GLEAN_10007795 | 3.33333 | 1 | 1 | 1 | 3.14976 | 6.519611 | 34.39 |  | FW16_GLEAN_10007795 | FW16_GLEAN_10007795 |  |  | FW16_GLEAN_10007795 |  |  | OXIDATION-REDUCTION | malate dehydrogenase |  |  |
| 262 | FW16_GLEAN_10007853 | 8 | 1 | 3 | 1 | 12.17 | 4.458012 | 22.94 | FW16_GLEAN_10007853 |  |  |  |  |  |  |  | NONE CONSERVED DOMAIN | hypothetical protein CDV31_010009 [Fusarium ambrosium] |  |  |
| 263 | FW16_GLEAN_10007861 | 31 | 6 | 167 | 6 | 725.26 | 8.180329 | 39.74 | FW16_GLEAN_10007861 |  | FW16_GLEAN_10007861 |  |  |  | FW16_GLEAN_10007861 | FW16_GLEAN_10007861 | CARBOHYDRATE METABOLISM |  | GH45-CBM1 | endoglucanase |
| 264 | FW16_GLEAN_10008102 | 5.14469 | 3 | 4 | 3 | 6.62188 | 4.930274 | 68.69 | FW16_GLEAN_10008102 | FW16_GLEAN_10008102 | FW16_GLEAN_10008102 | FW16_GLEAN_10008102 | FW16_GLEAN_10008102 | FW16_GLEAN_10008102 |  |  | OTHER BIOLOGICAL PROCESS | Actin |  |  |
| 265 | FW16_GLEAN_10008132 | 3.71901 | 1 | 1 | 1 | 1.8921 | 4.139532 | 23.59 | FW16_GLEAN_10008132 |  |  |  |  |  |  |  | NONE CONSERVED DOMAIN |  |  |  |
| 266 | FW16_GLEAN_10008150 | 6 | 1 | 4 | 1 | 19.14 | 4.227128 | 25.36 | FW16_GLEAN_10008150 |  |  |  |  |  |  |  | NONE CONSERVED DOMAIN | hypothetical protein FDECE_7264 [Fusarium decemcellulare] |  |  |
| 267 | FW16_GLEAN_10008188 | 3 | 2 | 14 | 2 | 52.72 | 8.205063 | 91.63 |  |  |  |  |  |  | FW16_GLEAN_10008188 | FW16_GLEAN_10008188 | CARBOHYDRATE METABOLISM |  | GH18-CBM18-CBM50-CBM50 | chitinase |
| 268 | FW16_GLEAN_10008214 | 9.47631 | 3 | 5 | 3 | 15.79 | 5.312397 | 41.89 | FW16_GLEAN_10008214 | FW16_GLEAN_10008214 | FW16_GLEAN_10008214 | FW16_GLEAN_10008214 | FW16_GLEAN_10008214 | FW16_GLEAN_10008214 | FW16_GLEAN_10008214 | FW16_GLEAN_10008214 | OXIDATION-REDUCTION | TIOREDOXIN REDUTASE |  |  |
| 269 | FW16_GLEAN_10008225 | 2.35507 | 1 | 3 | 1 | 9.2 | 4.354943 | 55.42 |  |  | FW16_GLEAN_10008225 |  |  |  |  |  | NONE CONSERVED DOMAIN |  |  |  |
| 270 | FW16_GLEAN_10008243 | 19 | 5 | 15 | 5 | 62.35 | 7.738795 | 49.86 |  |  |  |  |  |  | FW16_GLEAN_10008243 |  | CARBOHYDRATE METABOLISM |  | GH28 | polygalacturonase |
| 271 | FW16_GLEAN_10008261 | 15 | 6 | 46 | 6 | 180.75 | 4.433774 | 63.61 |  |  |  |  |  |  | FW16_GLEAN_10008261 | FW16_GLEAN_10008261 | NONE CONSERVED DOMAIN |  |  |  |
| 272 | FW16_GLEAN_10008317 | 4.29688 | 1 | 1 | 1 | 3.1398 | 10.54434 | 29.19 |  |  | FW16_GLEAN_10008317 |  |  |  |  |  | PROTEIN BIOSYNTHESIS | 40S ribosomal protein |  |  |
| 273 | FW16_GLEAN_10008357 | 6.76329 | 1 | 1 | 1 | 3.57727 | 7.375305 | 19.64 | FW16_GLEAN_10008357 |  | FW16_GLEAN_10008357 | FW16_GLEAN_10008357 |  | FW16_GLEAN_10008357 |  |  | OTHER BIOLOGICAL PROCESS | peptidyl-prolyl cis-trans isomerase B |  |  |
| 274 | FW16_GLEAN_10008361 | 9 | 1 | 15 | 1 | 74.45 | 3.761319 | 26.62 | FW16_GLEAN_10008361 |  |  |  |  |  |  |  | NONE CONSERVED DOMAIN | hypothetical protein BHE90_016344 [Fusarium euwallaceae] |  |  |
| 275 | FW16_GLEAN_10008397 | 7.95181 | 2 | 2 | 2 | 6.08689 | 5.112037 | 46.91 |  |  | FW16_GLEAN_10008397 | FW16_GLEAN_10008397 |  | FW16_GLEAN_10008397 |  |  | OTHER BIOLOGICAL PROCESS | elongation factor 1 gamma domain-containing protein / Glutathione S-transferase |  |  |
| 276 | FW16_GLEAN_10008463 | 7 | 1 | 10 | 1 | 36.21 | 7.029408 | 22.10 |  |  |  |  |  |  | FW16_GLEAN_10008463 | FW16_GLEAN_10008463 | CARBOHYDRATE METABOLISM |  | PL3 | pectate lyase |
| 277 | FW16_GLEAN_10008487 | 6.27178 | 1 | 1 | 1 | 2.41705 | 6.093103 | 31.98 |  | FW16_GLEAN_10008487 |  |  |  |  |  |  | RNA METABOLIC | CCR4-NOT complex subunit CAF16 |  |  |
| 278 | FW16_GLEAN_10008496 | 2.41758 | 1 | 1 | 1 | 2.62688 | 5.910596 | 49.31 |  |  |  |  | FW16_GLEAN_10008496 |  |  |  | OXIDATION-REDUCTION | glutamate dehydrogenase |  |  |
| 279 | FW16_GLEAN_10008505 | 2.02247 | 1 | 1 | 1 | 0 | 6.255551 | 49.12 | FW16_GLEAN_10008505 | FW16_GLEAN_10008505 |  |  |  |  |  |  | PROTEIN BIOSYNTHESIS | TUFM elongation factor Tu |  |  |
| 280 | FW16_GLEAN_10008508 | 8.55457 | 2 | 2 | 2 | 7.15105 | 11.23833 | 36.60 |  |  | FW16_GLEAN_10008508 |  |  |  |  |  | PROTEIN BIOSYNTHESIS | 60S ribosomal protein L4 |  |  |
| 281 | FW16_GLEAN_10008556 | 18.3771 | 7 | 153 | 7 | 441.65 | 5.6251 | 46.20 | FW16_GLEAN_10008556 | FW16_GLEAN_10008556 | FW16_GLEAN_10008556 | FW16_GLEAN_10008556 | FW16_GLEAN_10008556 | FW16_GLEAN_10008556 | FW16_GLEAN_10008556 | FW16_GLEAN_10008556 | OTHER BIOLOGICAL PROCESS | actin (cellular process, transport and catabolism) |  |  |
| 282 | FW16_GLEAN_10008616 | 76 | 9 | 139 | 9 | 632.67 | 3.960229 | 15.95 |  |  |  |  | FW16_GLEAN_10008616 |  | FW16_GLEAN_10008616 | FW16_GLEAN_10008616 | OTHER BIOLOGICAL PROCESS | DNase1 [Fusarium albosuccineum] |  |  |
| 283 | FW16_GLEAN_10008683 | 33 | 17 | 91 | 17 | 292.74 | 5.520916 | 72.80 |  |  |  |  |  |  | FW16_GLEAN_10008683 |  | CARBOHYDRATE METABOLISM |  | PL4 | rhamnogalacturonan endolyase |
| 284 | FW16_GLEAN_10008694 | 18.9041 | 7 | 7 | 7 | 13.5963 | 6.287066 | 39.86 | FW16_GLEAN_10008694 | FW16_GLEAN_10008694 | FW16_GLEAN_10008694 | FW16_GLEAN_10008694 | FW16_GLEAN_10008694 | FW16_GLEAN_10008694 |  |  | OXIDATION-REDUCTION | formate dehydrogenase |  |  |
| 285 | FW16_GLEAN_10008697 | 50 | 8 | 181 | 8 | 722.12 | 4.398019 | 25.42 | FW16_GLEAN_10008697 | FW16_GLEAN_10008697 | FW16_GLEAN_10008697 | FW16_GLEAN_10008697 | FW16_GLEAN_10008697 | FW16_GLEAN_10008697 | FW16_GLEAN_10008697 | FW16_GLEAN_10008697 | NONE CONSERVED DOMAIN |  |  |  |
| 286 | FW16_GLEAN_10008698 | 2.69784 | 1 | 1 | 1 | 3.7118 | 6.169523 | 60.23 |  |  | FW16_GLEAN_10008698 |  |  |  |  |  | CARBOHYDRATE METABOLISM |  | GH71 | α-1,3-glucanase |
| 287 | FW16_GLEAN_10008724 | 4.89796 | 1 | 1 | 1 | 3.01592 | 4.571445 | 26.88 |  |  | FW16_GLEAN_10008724 |  |  |  |  |  | OTHER BIOLOGICAL PROCESS | HAD hydrolase |  |  |
| 288 | FW16_GLEAN_10008797 | 17.2131 | 4 | 4 | 4 | 10.0498 | 6.493626 | 20.84 | FW16_GLEAN_10008797 | FW16_GLEAN_10008797 | FW16_GLEAN_10008797 | FW16_GLEAN_10008797 | FW16_GLEAN_10008797 |  |  |  | RNA METABOLIC | nucleoside-diphosphate kinase |  |  |
| 289 | FW16_GLEAN_10008834 | 13 | 6 | 36 | 6 | 132.64 | 4.586988 | 86.14 |  |  |  |  |  |  | FW16_GLEAN_10008834 |  | CARBOHYDRATE METABOLISM |  | GH3 | β-glucosidase /xylan 1,4-β-xylosidase |
| 290 | FW16_GLEAN_10008842 | 33 | 14 | 78 | 14 | 320.93 | 4.961968 | 68.54 |  | FW16_GLEAN_10008842 |  |  |  |  | FW16_GLEAN_10008842 | FW16_GLEAN_10008842 | CARBOHYDRATE METABOLISM |  | AA7 | glucooligosaccharide oxidase |
| 291 | FW16_GLEAN_10008893 | 34 | 8 | 59 | 7 | 191.53 | 6.481699 | 35.23 | FW16_GLEAN_10008893 | FW16_GLEAN_10008893 | FW16_GLEAN_10008893 |  |  | FW16_GLEAN_10008893 |  | FW16_GLEAN_10008893 | LIPID METABOLIC | Fungal lipase-like domain |  |  |
| 292 | FW16_GLEAN_10008926 | 7 | 2 | 4 | 2 | 10.23 | 4.592112 | 30.08 |  |  |  | FW16_GLEAN_10008926 |  |  |  | FW16_GLEAN_10008926 | OTHER BIOLOGICAL PROCESS | Survival protein SurE FAMILY |  |  |
| 293 | FW16_GLEAN_10008961 | 33 | 4 | 47 | 4 | 205.69 | 4.295626 | 31.22 | FW16_GLEAN_10008961 |  | FW16_GLEAN_10008961 |  |  |  | FW16_GLEAN_10008961 | FW16_GLEAN_10008961 | CARBOHYDRATE METABOLISM |  | GH18-CBM18 | chitinase |
| 294 | FW16_GLEAN_10008963 | 59 | 7 | 271 | 7 | 1154.19 | 8.429239 | 25.28 | FW16_GLEAN_10008963 |  |  | FW16_GLEAN_10008963 | FW16_GLEAN_10008963 |  | FW16_GLEAN_10008963 | FW16_GLEAN_10008963 | CARBOHYDRATE METABOLISM |  | GH12 | endoglucanase / xyloglucan hydrolase |
| 295 | FW16_GLEAN_10008990 | 13.5036 | 5 | 5 | 5 | 14.8547 | 5.342394 | 60.28 | FW16_GLEAN_10008990 | FW16_GLEAN_10008990 | FW16_GLEAN_10008990 | FW16_GLEAN_10008990 |  | FW16_GLEAN_10008990 |  |  | OTHER BIOLOGICAL PROCESS | Phosphoglucose isomerase |  |  |
| 296 | FW16_GLEAN_10009054 | 13 | 6 | 406 | 6 | 1369.41 | 4.977044 | 60.28 | FW16_GLEAN_10009054 | FW16_GLEAN_10009054 | FW16_GLEAN_10009054 | FW16_GLEAN_10009054 | FW16_GLEAN_10009054 |  | FW16_GLEAN_10009054 | FW16_GLEAN_10009054 | PROTEOLYSIS | SERINE CARBOXIPEPTIDASE |  |  |
| 297 | FW16_GLEAN_10009074 | 24 | 5 | 42 | 5 | 198.11 | 5.203712 | 38.21 |  |  |  |  |  |  |  | FW16_GLEAN_10009074 | PROTEOLYSIS | ASPARTYL PROTEASE/ASPARTIC PEPTIDASE A1 |  |  |
| 298 | FW16_GLEAN_10009112 | 1 | 1 | 1 | 1 | 4.11 | 3.25807 | 226.52 |  | FW16_GLEAN_10009112 |  |  |  |  |  |  | NONE CONSERVED DOMAIN | hypothetical protein CEP53_011843 [Fusarium sp. AF-6] |  |  |
| 299 | FW16_GLEAN_10009225 | 29.3333 | 4 | 6 | 4 | 18.3739 | 4.443382 | 22.12 | FW16_GLEAN_10009225 | FW16_GLEAN_10009225 | FW16_GLEAN_10009225 | FW16_GLEAN_10009225 | FW16_GLEAN_10009225 | FW16_GLEAN_10009225 | FW16_GLEAN_10009225 |  | CARBOHYDRATE METABOLISM |  | GH16 | xyloglucan:xyloglucosyltransferase / keratan-sulfate endo-1,4-β-galactosidase / endo-1,3-β-glucanase / laminarinase/ other |
| 300 | FW16_GLEAN_10009306 | 4.48625 | 1 | 1 | 1 | 0 | 6.653854 | 79.00 |  | FW16_GLEAN_10009306 |  |  |  |  |  |  | NONE CONSERVED DOMAIN | NECHADRAFT_99093 [Fusarium vanettenii 77-13-4] |  |  |
| 301 | FW16_GLEAN_10009310 | 3 | 1 | 1 | 1 | 2.9 | 5.559349 | 59.74 |  | FW16_GLEAN_10009310 |  |  |  |  |  |  | LIPID METABOLIC | triacylglycerol lipase |  |  |
| 302 | FW16_GLEAN_10009372 | 26 | 4 | 22 | 4 | 97.69 | 6.834366 | 21.68 |  |  |  |  |  |  | FW16_GLEAN_10009372 | FW16_GLEAN_10009372 | NONE CONSERVED DOMAIN |  |  |  |
| 303 | FW16_GLEAN_10009429 | 4.5283 | 1 | 1 | 1 | 0 | 6.265631 | 28.46 |  |  |  | FW16_GLEAN_10009429 |  |  |  |  | OXIDATION-REDUCTION | NAD dependent epimerase dehydratase [Fusarium albosuccineum] |  |  |
| 304 | FW16_GLEAN_10009433 | 2.37489 | 2 | 2 | 2 | 5.34396 | 4.86455 | 131.05 |  |  | FW16_GLEAN_10009433 | FW16_GLEAN_10009433 |  |  |  |  | OTHER BIOLOGICAL PROCESS | ubiquitin-activating enzyme E1 |  |  |
| 305 | FW16_GLEAN_10009562 | 7.33696 | 1 | 1 | 1 | 0 | 5.810372 | 40.85 | FW16_GLEAN_10009562 |  |  |  |  |  |  |  | OXIDATION-REDUCTION | oxidoreductase activit |  |  |
| 306 | FW16_GLEAN_10009621 | 7.98319 | 1 | 1 | 1 | 3.05112 | 4.611651 | 26.10 |  |  | FW16_GLEAN_10009621 |  |  |  |  |  | PROTEIN BIOSYNTHESIS | 20S proteasome subunit alpha 5 |  |  |
| 307 | FW16_GLEAN_10009674 | 24 | 3 | 10 | 3 | 41.05 | 4.833379 | 23.49 |  |  |  |  | FW16_GLEAN_10009674 | FW16_GLEAN_10009674 |  | FW16_GLEAN_10009674 | OTHER BIOLOGICAL PROCESS | spherulin-1b [Fusarium langsethiae] |  |  |
| 308 | FW16_GLEAN_10009682 | 2.47036 | 1 | 1 | 1 | 0 | 8.401176 | 111.63 | FW16_GLEAN_10009682 | FW16_GLEAN_10009682 | FW16_GLEAN_10009682 |  | FW16_GLEAN_10009682 | FW16_GLEAN_10009682 | FW16_GLEAN_10009682 | FW16_GLEAN_10009682 | OTHER BIOLOGICAL PROCESS | ABC transporter-like IPR003593; AAA+ ATPase domain |  |  |
| 309 | FW16_GLEAN_10009721 | 8 | 1 | 1 | 1 | 3.11 | 4.379453 | 25.80 | FW16_GLEAN_10009721 | FW16_GLEAN_10009721 | FW16_GLEAN_10009721 |  |  | FW16_GLEAN_10009721 |  |  | OTHER BIOLOGICAL PROCESS | hypothetical protein CEP54_011908 [Fusarium sp. AF-8] |  |  |
| 310 | FW16_GLEAN_10009722 | 17 | 2 | 11 | 2 | 43.79 | 8.419594 | 11.94 |  |  |  |  |  |  | FW16_GLEAN_10009722 | FW16_GLEAN_10009722 | OTHER BIOLOGICAL PROCESS | ACYL-CoA BINDING PROTEIN (ACBP) |  |  |
| 311 | FW16_GLEAN_10009774 | 6 | 4 | 60 | 1 | 209.26 | 3.890619 | 94.33 |  |  |  |  |  |  | FW16_GLEAN_10009774 | FW16_GLEAN_10009774 | CARBOHYDRATE METABOLISM |  | GH16 | xyloglucan:xyloglucosyltransferase / keratan-sulfate endo-1,4-β-galactosidase / endo-1,3-β-glucanase / laminarinase/ other |
| 312 | FW16_GLEAN_10009828 | 14 | 3 | 5 | 3 | 10.4 | 5.541626 | 28.41 |  |  |  |  |  |  |  | FW16_GLEAN_10009828 | OTHER BIOLOGICAL PROCESS | Gamma interferon inducible lysosomal thiol reductase (GILT) |  |  |
| 313 | FW16_GLEAN_10009840 | 17 | 4 | 31 | 4 | 94.95 | 6.772526 | 35.83 |  |  |  |  |  |  | FW16_GLEAN_10009840 | FW16_GLEAN_10009840 | CARBOHYDRATE METABOLISM |  | GH43 | β-xylosidase / α-L-arabinofuranosidase / xylanase |
| 314 | FW16_GLEAN_10009865 | 15.2778 | 2 | 2 | 2 | 4.11303 | 5.372719 | 15.28 |  |  |  | FW16_GLEAN_10009865 | FW16_GLEAN_10009865 |  |  |  | OTHER BIOLOGICAL PROCESS | MAPK signaling pathway |  |  |
| 315 | FW16_GLEAN_10009869 | 3.54839 | 1 | 1 | 1 | 2.51254 | 8.182375 | 33.86 | FW16_GLEAN_10009869 |  |  | FW16_GLEAN_10009869 |  |  |  |  | OTHER BIOLOGICAL PROCESS | GTP binding |  |  |
| 316 | FW16_GLEAN_10009882 | 4.36047 | 1 | 1 | 1 | 2.85144 | 9.765842 | 38.52 |  |  | FW16_GLEAN_10009882 |  |  |  |  |  | NONE CONSERVED DOMAIN |  |  |  |
| 317 | FW16_GLEAN_10009894 | 29 | 3 | 74 | 3 | 220.17 | 4.265561 | 17.16 | FW16_GLEAN_10009894 | FW16_GLEAN_10009894 | FW16_GLEAN_10009894 | FW16_GLEAN_10009894 |  | FW16_GLEAN_10009894 | FW16_GLEAN_10009894 | FW16_GLEAN_10009894 | OTHER BIOLOGICAL PROCESS | Tox1 [Fusarium virguliforme] |  |  |
| 318 | FW16_GLEAN_10009899 | 7.21154 | 5 | 19 | 5 | 67.36 | 6.321359 | 91.59 | FW16_GLEAN_10009899 |  | FW16_GLEAN_10009899 | FW16_GLEAN_10009899 | FW16_GLEAN_10009899 | FW16_GLEAN_10009899 |  |  | PROTEIN BIOSYNTHESIS | EF2_NEUCR Elongation factor 2 |  |  |
| 319 | FW16_GLEAN_10009915 | 7.22222 | 1 | 1 | 1 | 1.74262 | 4.170173 | 17.22 |  |  | FW16_GLEAN_10009915 | FW16_GLEAN_10009915 |  |  | FW16_GLEAN_10009915 | FW16_GLEAN_10009915 | LIPID METABOLIC | phosphatidylglycerol transfer protein |  |  |
| 320 | FW16_GLEAN_10009934 | 1.69492 | 1 | 1 | 1 | 1.77817 | 5.102231 | 72.28 |  |  |  | FW16_GLEAN_10009934 |  |  |  |  | OTHER BIOLOGICAL PROCESS | lysine-specific demethylase |  |  |
| 321 | FW16_GLEAN_10009999 | 2.79503 | 1 | 1 | 1 | 2.68449 | 4.811509 | 33.44 |  |  | FW16_GLEAN_10009999 | FW16_GLEAN_10009999 |  | FW16_GLEAN_10009999 |  |  | NONE CONSERVED DOMAIN |  |  |  |
| 322 | FW16_GLEAN_10010043 | 1 | 1 | 5 | 1 | 20.55 | 3.945898 | 263.42 | FW16_GLEAN_10010043 | FW16_GLEAN_10010043 |  |  |  |  |  |  | NONE CONSERVED DOMAIN | hypothetical protein CDV31_015585 [Fusarium ambrosium] |  |  |
| 323 | FW16_GLEAN_10010073 | 3.2634 | 1 | 1 | 1 | 2.1695 | 6.367354 | 47.63 |  |  |  | FW16_GLEAN_10010073 |  |  |  |  | AMINO ACID METABOLISM | Aminotransferases |  |  |
| 324 | FW16_GLEAN_10010110 | 8 | 2 | 6 | 2 | 22.91 | 5.088872 | 63.49 |  |  |  | FW16_GLEAN_10010110 |  |  |  |  | OTHER BIOLOGICAL PROCESS | PREDICTED EXTRACELLULAR NUCLEASE |  |  |
| 325 | FW16_GLEAN_10010157 | 12.3675 | 6 | 8 | 6 | 15.9106 | 8.977295 | 59.89 | FW16_GLEAN_10010157 | FW16_GLEAN_10010157 | FW16_GLEAN_10010157 |  |  |  |  |  | AMINO ACID METABOLISM | tyrosinase |  |  |
| 326 | FW16_GLEAN_10010166 | 5 | 3 | 45 | 1 | 131.61 | 4.46965 | 49.81 | FW16_GLEAN_10010166 | FW16_GLEAN_10010166 | FW16_GLEAN_10010166 |  | FW16_GLEAN_10010166 | FW16_GLEAN_10010166 | FW16_GLEAN_10010166 |  | OTHER BIOLOGICAL PROCESS | tubulin beta |  |  |
| 327 | FW16_GLEAN_10010168 | 3.93258 | 1 | 1 | 1 | 0 | 5.270276 | 37.73 |  |  |  | FW16_GLEAN_10010168 |  |  |  |  | OTHER BIOLOGICAL PROCESS | 3'(2'), 5'-bisphosphate nucleotidase |  |  |
| 328 | FW16_GLEAN_10010201 | 16 | 5 | 45 | 5 | 146.74 | 4.285813 | 42.88 |  |  |  |  | FW16_GLEAN_10010201 |  | FW16_GLEAN_10010201 | FW16_GLEAN_10010201 | CARBOHYDRATE METABOLISM |  | GH16 | xyloglucan:xyloglucosyltransferase / keratan-sulfate endo-1,4-β-galactosidase / endo-1,3-β-glucanase / laminarinase/ other |
| 329 | FW16_GLEAN_10010213 | 24 | 5 | 28 | 5 | 90.23 | 4.101139 | 24.44 | FW16_GLEAN_10010213 | FW16_GLEAN_10010213 |  | FW16_GLEAN_10010213 | FW16_GLEAN_10010213 |  | FW16_GLEAN_10010213 | FW16_GLEAN_10010213 | OTHER BIOLOGICAL PROCESS | complex i intermediate-associated 30 [Fusarium sporotrichioides] |  |  |
| 330 | FW16_GLEAN_10010243 | 4 | 3 | 21 | 3 | 86.63 | 5.021943 | 138.30 |  |  |  |  |  |  | FW16_GLEAN_10010243 | FW16_GLEAN_10010243 | OTHER BIOLOGICAL PROCESS | PATCHED SUPER FAMILY |  |  |
| 331 | FW16_GLEAN_10010327 | 3 | 1 | 3 | 1 | 11.82 | 6.073726 | 55.48 | FW16_GLEAN_10010327 | FW16_GLEAN_10010327 |  |  |  |  |  |  | OTHER BIOLOGICAL PROCESS | Sugar/inositol transporter |  |  |
| 332 | FW16_GLEAN_10010329 | 3.33333 | 1 | 1 | 1 | 2.75924 | 5.329556 | 73.39 |  | FW16_GLEAN_10010329 |  |  |  |  |  |  | NONE CONSERVED DOMAIN |  |  |  |
| 333 | FW16_GLEAN_10010356 | 11 | 2 | 5 | 2 | 19.76 | 4.124053 | 32.29 |  |  |  |  |  |  | FW16_GLEAN_10010356 | FW16_GLEAN_10010356 | CARBOHYDRATE METABOLISM |  | GH11-CBM1 | endo-β-1,4-xylanase / endo-β-1,3-xylanase |
| 334 | FW16_GLEAN_10010396 | 1 | 1 | 4 | 1 | 9.19 | 4.29875 | 87.04 | FW16_GLEAN_10010396 | FW16_GLEAN_10010396 |  |  |  |  |  |  | NONE CONSERVED DOMAIN | hypothetical protein BHE90_000658 [Fusarium euwallaceae] |  |  |
| 335 | FW16_GLEAN_10010400 | 40 | 19 | 359 | 19 | 1352.7 | 5.028947 | 57.90 |  |  |  |  |  |  | FW16_GLEAN_10010400 | FW16_GLEAN_10010400 | PROTEOLYSIS | PEPTIDASE S28 |  |  |
| 336 | FW16_GLEAN_10010545 | 3.01811 | 1 | 1 | 1 | 3.30702 | 5.606826 | 51.63 | FW16_GLEAN_10010545 | FW16_GLEAN_10010545 |  |  |  |  |  | FW16_GLEAN_10010545 | PROTEOLYSIS | aminopeptidase S |  |  |
| 337 | FW16_GLEAN_10010580 | 1.78784 | 1 | 1 | 1 | 3.38749 | 5.085534 | 95.62 |  |  | FW16_GLEAN_10010580 |  |  |  |  |  | CARBOHYDRATE METABOLISM |  | GH2 | β-galactosidase / β-mannosidase / β-glucuronidase |
| 338 | FW16_GLEAN_10010582 | 34.9727 | 5 | 5 | 5 | 12.8246 | 4.799471 | 18.47 | FW16_GLEAN_10010582 | FW16_GLEAN_10010582 | FW16_GLEAN_10010582 |  |  | FW16_GLEAN_10010582 |  |  | CARBOHYDRATE METABOLISM |  | GH134 | endo-β-1,4-mannanase |
| 339 | FW16_GLEAN_10010710 | 10 | 2 | 8 | 2 | 35.49 | 5.220487 | 48.09 |  |  |  |  |  |  | FW16_GLEAN_10010710 | FW16_GLEAN_10010710 | OTHER BIOLOGICAL PROCESS | FASCICLIN SUPERFAMILY |  |  |
| 340 | FW16_GLEAN_10010746 | 2.1021 | 1 | 1 | 1 | 1.75864 | 7.385471 | 36.89 |  | FW16_GLEAN_10010746 |  |  |  |  |  |  | NONE CONSERVED DOMAIN | hypothetical protein BFJ70_g17149 [Fusarium oxysporum] |  |  |
| 341 | FW16_GLEAN_10010764 | 7.30337 | 1 | 1 | 1 | 2.27876 | 4.920089 | 19.75 | FW16_GLEAN_10010764 | FW16_GLEAN_10010764 |  |  |  | FW16_GLEAN_10010764 |  |  | OTHER BIOLOGICAL PROCESS | Alpha/Beta hydrolase fold |  |  |
| 342 | FW16_GLEAN_10010850 | 1.52027 | 1 | 1 | 1 | 1.64334 | 4.787346 | 64.60 |  |  |  | FW16_GLEAN_10010850 |  |  |  |  | OTHER BIOLOGICAL PROCESS | fungal-type DNA-binding domain |  |  |
| 343 | FW16_GLEAN_10010890 | 5 | 3 | 31 | 3 | 101.82 | 4.888301 | 77.74 |  |  |  |  |  |  | FW16_GLEAN_10010890 | FW16_GLEAN_10010890 | CARBOHYDRATE METABOLISM |  | GH17 | glucan endo-1,3-β-glucosidase |
| 344 | FW16_GLEAN_10010955 | 6.42202 | 2 | 2 | 2 | 2.56247 | 4.519542 | 36.75 | FW16_GLEAN_10010955 |  |  |  |  |  |  |  | CARBOHYDRATE METABOLISM |  | GH43 | β-xylosidase / α-L-arabinofuranosidase / xylanase |
| 345 | FW16_GLEAN_10011090 | 3.9783 | 1 | 1 | 1 | 3.35876 | 7.554726 | 60.87 |  |  | FW16_GLEAN_10011090 |  |  |  |  |  | OXIDATION-REDUCTION | 2-methylcitrate dehydratase |  |  |
| 346 | FW16_GLEAN_10011145 | 14 | 5 | 14 | 5 | 40.45 | 8.57593 | 51.63 |  |  |  |  |  |  | FW16_GLEAN_10011145 |  | LIPID METABOLIC |  |  |  |
| 347 | FW16_GLEAN_10011178 | 12.0482 | 4 | 31 | 4 | 83.95 | 9.514788 | 54.16 | FW16_GLEAN_10011178 | FW16_GLEAN_10011178 | FW16_GLEAN_10011178 | FW16_GLEAN_10011178 | FW16_GLEAN_10011178 | FW16_GLEAN_10011178 | FW16_GLEAN_10011178 | FW16_GLEAN_10011178 | PROTEIN BIOSYNTHESIS | EEF1A elongation factor 1-alpha |  |  |
| 348 | FW16_GLEAN_10011190 | 4 | 1 | 2 | 1 | 4.71 | 5.126499 | 29.14 | FW16_GLEAN_10011190 | FW16_GLEAN_10011190 | FW16_GLEAN_10011190 | FW16_GLEAN_10011190 |  |  |  | FW16_GLEAN_10011190 | OXIDATION-REDUCTION | FMN binding; molecular_function GO:0016491; oxidoreductase activity |  |  |
| 349 | FW16_GLEAN_10011227 | 2 | 1 | 1 | 1 | 2.4 | 3.915292 | 50.74 |  | FW16_GLEAN_10011227 |  |  |  |  |  |  | PROTEOLYSIS | Aspartic peptidase |  |  |
| 350 | FW16_GLEAN_10011231 | 5 | 3 | 7 | 3 | 27.38 | 3.844637 | 97.64 |  |  | FW16_GLEAN_10011231 |  |  |  | FW16_GLEAN_10011231 | FW16_GLEAN_10011231 | CARBOHYDRATE METABOLISM |  | GH18 | chitinase |
| 351 | FW16_GLEAN_10011242 | 2.04678 | 1 | 1 | 1 | 1.76745 | 9.067724 | 37.86 | FW16_GLEAN_10011242 |  |  |  |  |  |  |  | RNA METABOLIC | fruit fly brahma transcriptional activator [Fusarium acutatum] |  |  |
| 352 | FW16_GLEAN_10011249 | 9 | 2 | 6 | 2 | 19.43 | 3.574113 | 48.01 | FW16_GLEAN_10011249 | FW16_GLEAN_10011249 | FW16_GLEAN_10011249 |  |  |  |  |  | NONE CONSERVED DOMAIN | hypothetical protein BHE90_009887 [Fusarium euwallaceae] |  |  |
| 353 | FW16_GLEAN_10011298 | 10.2941 | 3 | 5 | 3 | 15.259 | 6.293786 | 46.09 |  |  | FW16_GLEAN_10011298 |  |  |  |  |  | PROTEIN BIOSYNTHESIS | Translation elongation factor IF5A |  |  |
| 354 | FW16_GLEAN_10011330 | 5.06329 | 1 | 1 | 1 | 2.1783 | 11.73821 | 17.76 |  |  | FW16_GLEAN_10011330 |  |  |  |  |  | PROTEIN BIOSYNTHESIS | 60S ribosomal protein L24 |  |  |
| 355 | FW16_GLEAN_10011363 | 6.51341 | 2 | 2 | 2 | 4.32007 | 10.57354 | 29.56 |  |  | FW16_GLEAN_10011363 |  |  |  |  |  | PROTEIN BIOSYNTHESIS | 40S ribosomal protein S4 |  |  |
| 356 | FW16_GLEAN_10011373 | 30.4348 | 8 | 11 | 8 | 38.36 | 5.403837 | 35.41 | FW16_GLEAN_10011373 | FW16_GLEAN_10011373 | FW16_GLEAN_10011373 | FW16_GLEAN_10011373 | FW16_GLEAN_10011373 | FW16_GLEAN_10011373 |  |  | OXIDATION-REDUCTION | talB transaldolase |  |  |
| 357 | FW16_GLEAN_10011385 | 8.84354 | 1 | 1 | 1 | 3.00396 | 10.90618 | 16.65 |  |  | FW16_GLEAN_10011385 |  |  |  |  |  | PROTEIN BIOSYNTHESIS | 60S ribosomal protein L14 |  |  |
| 358 | FW16_GLEAN_10011396 | 18 | 2 | 17 | 2 | 68.37 | 3.589926 | 19.82 |  |  |  |  |  |  | FW16_GLEAN_10011396 | FW16_GLEAN_10011396 | OTHER BIOLOGICAL PROCESS | CFEM DOMAIN (RELATED TO FUNGAL PHATOGENESIS |  |  |
| 359 | FW16_GLEAN_10011463 | 6 | 2 | 9 | 2 | 40.71 | 6.226162 | 117.53 | FW16_GLEAN_10011463 | FW16_GLEAN_10011463 |  | FW16_GLEAN_10011463 |  |  |  |  | OTHER BIOLOGICAL PROCESS | polyubiquitin protein |  |  |
| 360 | FW16_GLEAN_10011474 | 7.86385 | 6 | 6 | 6 | 10.7789 | 8.704061 | 91.29 | FW16_GLEAN_10011474 | FW16_GLEAN_10011474 | FW16_GLEAN_10011474 |  |  | FW16_GLEAN_10011474 |  |  | CARBOHYDRATE METABOLISM |  | GH81 | endo-β-1,3-glucanase (EC 3.2.1.39) |
| 361 | FW16_GLEAN_10011482 | 1.79153 | 1 | 1 | 1 | 2.3371 | 4.775262 | 137.47 |  | FW16_GLEAN_10011482 |  |  |  | FW16_GLEAN_10011482 |  |  | PROTEIN BIOSYNTHESIS | large subunit ribosomal protein L41 |  |  |
| 362 | FW16_GLEAN_10011486 | 6.80751 | 1 | 1 | 1 | 0 | 4.205835 | 46.59 | FW16_GLEAN_10011486 |  |  |  |  |  |  |  | NONE CONSERVED DOMAIN |  |  |  |
| 363 | FW16_GLEAN_10011543 | 4.13534 | 1 | 1 | 1 | 2.74973 | 10.77638 | 29.82 |  |  | FW16_GLEAN_10011543 |  |  |  |  |  | PROTEIN BIOSYNTHESIS | 60S ribosomal protein L8 |  |  |
| 364 | FW16_GLEAN_10011615 | 16 | 7 | 32 | 7 | 99.87 | 5.476476 | 57.51 |  |  |  |  |  |  | FW16_GLEAN_10011615 |  | CARBOHYDRATE METABOLISM |  | GH32 | invertase / endo-inulinase/ other |
| 365 | FW16_GLEAN_10011639 | 27 | 13 | 109 | 13 | 429.28 | 5.051679 | 82.24 |  |  |  |  |  |  | FW16_GLEAN_10011639 | FW16_GLEAN_10011639 | CARBOHYDRATE METABOLISM |  | GH3 | β-glucosidase /xylan 1,4-β-xylosidase |
| 366 | FW16_GLEAN_10011671 | 0.899281 | 1 | 1 | 1 | 2.72703 | 7.46876 | 123.43 |  |  |  | FW16_GLEAN_10011671 |  |  |  |  | OTHER BIOLOGICAL PROCESS | sodium/potassium-transporting ATPase subunit alpha |  |  |
| 367 | FW16_GLEAN_10011672 | 8.52273 | 1 | 1 | 1 | 3.41268 | 9.138466 | 19.41 |  |  | FW16_GLEAN_10011672 |  |  |  |  |  | NONE CONSERVED DOMAIN |  |  |  |
| 368 | FW16_GLEAN_10011717 | 15.0943 | 2 | 2 | 2 | 7.76061 | 11.62685 | 23.76 |  |  | FW16_GLEAN_10011717 |  |  |  |  |  | PROTEIN BIOSYNTHESIS | 60S ribosomal protein L13 |  |  |
| 369 | FW16_GLEAN_10011762 | 8.69565 | 1 | 1 | 1 | 0 | 4.187443 | 15.09 | FW16_GLEAN_10011762 |  |  |  |  |  |  |  | NONE CONSERVED DOMAIN |  |  |  |
| 370 | FW16_GLEAN_10011765 | 6 | 2 | 2 | 2 | 5.79 | 4.244736 | 45.48 | FW16_GLEAN_10011765 |  |  |  |  |  | FW16_GLEAN_10011765 |  | OTHER BIOLOGICAL PROCESS | atrophin 1 - WSC binding domain |  |  |
| 371 | FW16_GLEAN_10011883 | 23 | 13 | 57 | 13 | 215.14 | 4.975612 | 83.81 |  |  |  |  |  |  | FW16_GLEAN_10011883 |  | CARBOHYDRATE METABOLISM |  | GH3 | β-glucosidase /xylan 1,4-β-xylosidase |
| 372 | FW16_GLEAN_10011887 | 31 | 4 | 14 | 4 | 45.12 | 4.347411 | 17.63 |  |  |  |  |  |  | FW16_GLEAN_10011887 | FW16_GLEAN_10011887 | NONE CONSERVED DOMAIN |  |  |  |
| 373 | FW16_GLEAN_10011917 | 66 | 19 | 840 | 19 | 3367.6 | 5.571232 | 40.58 |  |  |  |  |  |  | FW16_GLEAN_10011917 | FW16_GLEAN_10011917 | CARBOHYDRATE METABOLISM |  | GH93 | exo-α-L-1,5-arabinanase |
| 374 | FW16_GLEAN_10011918 | 12 | 3 | 32 | 3 | 88.82 | 4.767336 | 35.87 |  |  |  |  |  |  | FW16_GLEAN_10011918 | FW16_GLEAN_10011918 | CARBOHYDRATE METABOLISM |  | GH43 | β-xylosidase / α-L-arabinofuranosidase / xylanase |
| 375 | FW16_GLEAN_10011993 | 1.77305 | 1 | 2 | 1 | 5.7208 | 5.57422 | 62.07 |  |  | FW16_GLEAN_10011993 |  |  |  |  |  | OTHER BIOLOGICAL PROCESS | Sugar/inositol transporter |  |  |
| 376 | FW16_GLEAN_10011996 | 3 | 1 | 1 | 1 | 3.34 | 7.262535 | 42.66 |  | FW16_GLEAN_10011996 | FW16_GLEAN_10011996 | FW16_GLEAN_10011996 | FW16_GLEAN_10011996 | FW16_GLEAN_10011996 |  |  | CARBOHYDRATE METABOLISM |  | CE8 | Pectinesterase |
| 377 | FW16_GLEAN_10012002 | 27.1277 | 4 | 25 | 4 | 88.83 | 5.825287 | 17.18 | FW16_GLEAN_10012002 | FW16_GLEAN_10012002 |  | FW16_GLEAN_10012002 | FW16_GLEAN_10012002 | FW16_GLEAN_10012002 | FW16_GLEAN_10012002 |  | OTHER BIOLOGICAL PROCESS | cell wall [Fusarium albosuccineum] |  |  |
| 378 | FW16_GLEAN_10012035 | 4 | 1 | 1 | 1 | 3.25 | 4.302308 | 32.27 |  |  | FW16_GLEAN_10012035 | FW16_GLEAN_10012035 |  |  |  |  | CARBOHYDRATE METABOLISM |  | PL3 | pectate lyase |
| 379 | FW16_GLEAN_10012037 | 43 | 8 | 67 | 8 | 242.33 | 6.165258 | 32.21 |  |  |  |  |  |  | FW16_GLEAN_10012037 | FW16_GLEAN_10012037 | CARBOHYDRATE METABOLISM |  | PL1 | pectate lyase |
| 380 | FW16_GLEAN_10012038 | 16 | 4 | 24 | 4 | 101.35 | 4.231765 | 44.35 |  |  |  |  |  |  |  | FW16_GLEAN_10012038 | CARBOHYDRATE METABOLISM |  | GH43 | β-xylosidase / α-L-arabinofuranosidase / xylanase |
| 381 | FW16_GLEAN_10012076 | 2 | 1 | 2 | 1 | 6.5 | 4.485134 | 132.59 | FW16_GLEAN_10012076 | FW16_GLEAN_10012076 | FW16_GLEAN_10012076 | FW16_GLEAN_10012076 | FW16_GLEAN_10012076 | FW16_GLEAN_10012076 |  |  | NONE CONSERVED DOMAIN | hypothetical protein CEP53_007904 [Fusarium sp. AF-6] |  |  |
| 382 | FW16_GLEAN_10012096 | 11.1111 | 2 | 19 | 1 | 45.48 | 4.742007 | 27.82 | FW16_GLEAN_10012096 | FW16_GLEAN_10012096 | FW16_GLEAN_10012096 | FW16_GLEAN_10012096 |  | FW16_GLEAN_10012096 |  |  | LIPID METABOLIC | lipase [Fusarium heterosporum] |  |  |
| 383 | FW16_GLEAN_10012117 | 21 | 7 | 54 | 7 | 180.24 | 5.006737 | 65.57 | FW16_GLEAN_10012117 |  |  |  |  |  |  | FW16_GLEAN_10012117 | CARBOHYDRATE METABOLISM |  | GH49 | dextranase / isopullulanase |
| 384 | FW16_GLEAN_10012126 | 13.5802 | 1 | 1 | 1 | 0 | 5.174491 | 28.04 | FW16_GLEAN_10012126 | FW16_GLEAN_10012126 | FW16_GLEAN_10012126 | FW16_GLEAN_10012126 | FW16_GLEAN_10012126 | FW16_GLEAN_10012126 |  |  | NONE CONSERVED DOMAIN | hypothetical protein CEP52_013857 [Fusarium sp. AF-4] |  |  |
| 385 | FW16_GLEAN_10012179 | 16 | 4 | 27 | 4 | 105.07 | 4.616436 | 39.10 |  |  |  |  |  |  | FW16_GLEAN_10012179 | FW16_GLEAN_10012179 | OTHER BIOLOGICAL PROCESS | PERIPLASMATIC BINDING PROTEIN |  |  |
| 386 | FW16_GLEAN_10012182 | 16.5414 | 3 | 3 | 3 | 10.5054 | 5.01177 | 28.44 |  |  | FW16_GLEAN_10012182 | FW16_GLEAN_10012182 | FW16_GLEAN_10012182 | FW16_GLEAN_10012182 |  | FW16_GLEAN_10012182 | CARBOHYDRATE METABOLISM |  | GH99 | glycoprotein endo-α-1,2-mannosidase / mannan endo-1,2-α-mannanase |
| 387 | FW16_GLEAN_10012229 | 79 | 15 | 602 | 15 | 2393.7 | 7.844883 | 25.58 | FW16_GLEAN_10012229 | FW16_GLEAN_10012229 | FW16_GLEAN_10012229 |  | FW16_GLEAN_10012229 | FW16_GLEAN_10012229 | FW16_GLEAN_10012229 | FW16_GLEAN_10012229 | CARBOHYDRATE METABOLISM |  | CE12 | pectin acetylesterase/ rhamnogalacturonan acetylesterase / acetyl xylan esterase |
| 388 | FW16_GLEAN_10012243 | 17 | 6 | 46 | 6 | 179.55 | 4.836422 | 61.24 |  |  |  |  |  |  | FW16_GLEAN_10012243 | FW16_GLEAN_10012243 | LIPID METABOLIC |  |  |  |
| 389 | FW16_GLEAN_10012260 | 1.79283 | 1 | 1 | 1 | 2.70449 | 6.198906 | 53.18 |  |  | FW16_GLEAN_10012260 |  |  | FW16_GLEAN_10012260 |  |  | CARBOHYDRATE METABOLISM |  | AA7 | glucooligosaccharide oxidase / chitooligosaccharide oxidase |
| 390 | FW16_GLEAN_10012310 | 2.70655 | 1 | 1 | 1 | 3.17044 | 7.659983 | 80.29 |  |  |  |  |  | FW16_GLEAN_10012310 |  |  | NONE CONSERVED DOMAIN |  |  |  |
| 391 | FW16_GLEAN_10012330 | 8.58209 | 1 | 1 | 1 | 3.48178 | 8.717091 | 28.20 | FW16_GLEAN_10012330 |  |  |  |  |  |  |  | OXIDATION-REDUCTION | Glucose/ribitol dehydrogenase |  |  |
| 392 | FW16_GLEAN_10012346 | 2.38532 | 2 | 2 | 2 | 4.56257 | 4.540277 | 56.59 |  |  |  | FW16_GLEAN_10012346 |  |  |  |  | NONE CONSERVED DOMAIN |  |  |  |
| 393 | FW16_GLEAN_10012377 | 2 | 3 | 4 | 3 | 11.01 | 5.370837 | 187.84 |  |  |  | FW16_GLEAN_10012377 |  |  |  |  | PROTEOLYSIS | Gamma-glutamyltranspeptidase |  |  |
| 394 | FW16_GLEAN_10012410 | 3 | 1 | 1 | 1 | 3.28 | 8.574467 | 40.83 |  |  |  | FW16_GLEAN_10012410 |  |  |  |  | NONE CONSERVED DOMAIN | hypothetical protein BHE90_002710 [Fusarium euwallaceae] |  |  |
| 395 | FW16_GLEAN_10012425 | 3.05164 | 1 | 1 | 1 | 3.56881 | 5.33136 | 47.80 |  |  |  | FW16_GLEAN_10012425 | FW16_GLEAN_10012425 |  |  |  | OTHER BIOLOGICAL PROCESS | transcription factor activity |  |  |
| 396 | FW16_GLEAN_10012445 | 1.81488 | 1 | 1 | 1 | 2.58108 | 6.503593 | 62.30 | FW16_GLEAN_10012445 |  | FW16_GLEAN_10012425 |  |  | FW16_GLEAN_10012445 |  |  | NONE CONSERVED DOMAIN |  |  |  |
| 397 | FW16_GLEAN_10012503 | 6.94981 | 1 | 1 | 1 | 4.23371 | 5.401829 | 29.27 |  | FW16_GLEAN_10012503 |  |  |  |  |  |  | OTHER BIOLOGICAL PROCESS | eukaryotic translation initiation factor 5A [Fusarium albosuccineum] |  |  |
| 398 | FW16_GLEAN_10012515 | 6 | 1 | 1 | 1 | 2.88 | 5.276946 | 29.53 | FW16_GLEAN_10012515 |  | FW16_GLEAN_10012515 | FW16_GLEAN_10012515 |  |  |  |  | OTHER BIOLOGICAL PROCESS | Quinoprotein amine dehydrogenase |  |  |
| 399 | FW16_GLEAN_10012536 | 38 | 7 | 85 | 7 | 389.74 | 6.350555 | 32.14 |  | FW16_GLEAN_10012536 |  |  |  |  | FW16_GLEAN_10012536 | FW16_GLEAN_10012536 | CARBOHYDRATE METABOLISM |  | GH17 | glucan endo-1,3-β-glucosidase |
| 400 | FW16_GLEAN_10012539 | 3.85675 | 1 | 1 | 1 | 3.27392 | 5.125842 | 38.36 | FW16_GLEAN_10012539 |  | FW16_GLEAN_10012539 | FW16_GLEAN_10012539 |  |  |  |  | AMINO ACID METABOLISM | leuB 3-isopropylmalate dehydrogenase |  |  |
| 401 | FW16_GLEAN_10012582 | 7.07071 | 1 | 1 | 1 | 3.71636 | 7.145599 | 21.68 | FW16_GLEAN_10012582 |  | FW16_GLEAN_10012582 |  |  |  |  |  | LIPID METABOLIC | Glycolipid transfer protein domain |  |  |
| 402 | FW16_GLEAN_10012607 | 3 | 1 | 9 | 1 | 40.71 | 6.465049 | 70.05 | FW16_GLEAN_10012607 | FW16_GLEAN_10012607 |  | FW16_GLEAN_10012607 |  |  |  |  | PROTEIN BIOSYNTHESIS | Ribosomal protein L40e |  |  |
| 403 | FW16_GLEAN_10012613 | 3.40426 | 2 | 3 | 2 | 7.20361 | 4.873228 | 77.91 |  |  | FW16_GLEAN_10012613 |  |  |  |  |  | OTHER BIOLOGICAL PROCESS | HSPA4 heat shock 70kDa protein 4 |  |  |
| 404 | FW16_GLEAN_10012654 | 5.45455 | 1 | 1 | 1 | 2.66051 | 9.827819 | 17.77 | FW16_GLEAN_10012654 |  |  | FW16_GLEAN_10012654 |  |  |  |  | PROTEIN BIOSYNTHESIS | large subunit ribosomal protein L12e |  |  |
| 405 | FW16_GLEAN_10012787 | 11.1663 | 2 | 9 | 2 | 31.31 | 5.967564 | 42.42 |  |  | FW16_GLEAN_10012787 |  |  |  |  | FW16_GLEAN_10012787 | AMINO ACID METABOLISM | tyrosinase |  |  |
| 406 | FW16_GLEAN_10012814 | 10.3841 | 6 | 82 | 6 | 305.19 | 4.61275 | 79.67 | FW16_GLEAN_10012814 | FW16_GLEAN_10012814 | FW16_GLEAN_10012814 | FW16_GLEAN_10012814 | FW16_GLEAN_10012814 | FW16_GLEAN_10012814 | FW16_GLEAN_10012814 | FW16_GLEAN_10012814 | OTHER BIOLOGICAL PROCESS | HSP90A molecular chaperone |  |  |
| 407 | FW16_GLEAN_10012817 | 41 | 17 | 194 | 17 | 725.35 | 4.27387 | 56.03 | FW16_GLEAN_10012817 | FW16_GLEAN_10012817 | FW16_GLEAN_10012817 | FW16_GLEAN_10012817 | FW16_GLEAN_10012817 | FW16_GLEAN_10012817 | FW16_GLEAN_10012817 | FW16_GLEAN_10012817 | CARBOHYDRATE METABOLISM |  | GH72-CBM43 | β-1,3-glucanosyltransglycosylase |
| 408 | FW16_GLEAN_10012854 | 8.55263 | 1 | 1 | 1 | 4.2858 | 11.27727 | 16.18 |  | FW16_GLEAN_10012854 |  |  |  |  |  |  | PROTEIN BIOSYNTHESIS | 40S ribosomal protein S14 |  |  |
| 409 | FW16_GLEAN_10012865 | 5.91837 | 1 | 1 | 1 | 0 | 9.6656 | 53.51 |  |  |  | FW16_GLEAN_10012865 |  |  |  |  | OTHER BIOLOGICAL PROCESS | integral component of membrane |  |  |
| 410 | FW16_GLEAN_10012881 | 5.51181 | 1 | 1 | 1 | 3.15697 | 5.520252 | 25.68 |  |  |  |  | FW16_GLEAN_10012881 |  |  |  | OXIDATION-REDUCTION | Manganese/iron superoxide dismutase |  |  |
| 411 | FW16_GLEAN_10012888 | 16.5354 | 1 | 1 | 1 | 3.40418 | 8.670388 | 11.89 |  | FW16_GLEAN_10012888 |  |  |  |  |  |  | OTHER BIOLOGICAL PROCESS | Manganese/iron superoxide dismutase |  |  |
| 412 | FW16_GLEAN_10012908 | 11.8785 | 3 | 3 | 3 | 8.15546 | 6.221656 | 40.08 |  |  | FW16_GLEAN_10012908 |  |  |  |  |  | OXIDATION-REDUCTION | scyllo-inositol 2-dehydrogenase |  |  |
| 413 | FW16_GLEAN_10012910 | 22 | 5 | 106 | 5 | 395.96 | 3.666408 | 17.57 |  |  |  |  |  |  | FW16_GLEAN_10012910 | FW16_GLEAN_10012910 | OTHER BIOLOGICAL PROCESS | CFEM DOMAIN (RELATED TO FUNGAL PHATOGENESIS) |  |  |
| 414 | FW16_GLEAN_10012918 | 2.11082 | 1 | 1 | 1 | 2.12893 | 6.26387 | 40.37 |  |  | FW16_GLEAN_10012918 |  |  |  |  |  | OXIDATION-REDUCTION | Alcohol dehydrogenase superfamily |  |  |
| 415 | FW16_GLEAN_10012953 | 6.84932 | 1 | 1 | 1 | 2.16443 | 7.246517 | 13.70 | FW16_GLEAN_10012953 |  |  | FW16_GLEAN_10012953 | FW16_GLEAN_10012953 |  |  |  | NONE CONSERVED DOMAIN |  |  |  |
| 416 | FW16_GLEAN_10012966 | 40 | 4 | 7 | 4 | 23.23 | 4.568624 | 17.02 |  |  |  |  |  |  | FW16_GLEAN_10012966 |  | OTHER BIOLOGICAL PROCESS | HISTIDINE PHOSPHATASE |  |  |
| 417 | FW16_GLEAN_10013031 | 17 | 3 | 10 | 3 | 32.01 | 4.278182 | 31.26 |  |  | FW16_GLEAN_10013031 |  | FW16_GLEAN_10013031 |  |  |  | OTHER BIOLOGICAL PROCESS | surface SP1 [Fusarium albosuccineum] |  |  |
| 418 | FW16_GLEAN_10013032 | 41 | 11 | 176 | 11 | 669.29 | 5.007815 | 42.61 |  |  |  |  |  |  | FW16_GLEAN_10013032 | FW16_GLEAN_10013032 | OTHER BIOLOGICAL PROCESS | GALACTOSE MUTAROTASE |  |  |
| 419 | FW16_GLEAN_10013060 | 13 | 2 | 9 | 2 | 43.23 | 5.483604 | 40.85 |  |  |  | FW16_GLEAN_10013060 |  |  |  |  | PROTEOLYSIS | Serine protease |  |  |
| 420 | FW16_GLEAN_10013150 | 1.84995 | 1 | 1 | 1 | 0 | 6.883498 | 108.34 |  |  | FW16_GLEAN_10013150 | FW16_GLEAN_10013150 |  |  |  |  | NONE CONSERVED DOMAIN | NECHADRAFT_69762 [Fusarium vanettenii 77-13-4] |  |  |
| 421 | FW16_GLEAN_10013206 | 15 | 5 | 16 | 5 | 59.04 | 6.013125 | 53.27 | FW16_GLEAN_10013206 | FW16_GLEAN_10013206 | FW16_GLEAN_10013206 | FW16_GLEAN_10013206 | FW16_GLEAN_10013206 | FW16_GLEAN_10013206 | FW16_GLEAN_10013206 | FW16_GLEAN_10013206 | PROTEOLYSIS | PEPTIDASE |  |  |
| 422 | FW16_GLEAN_10013250 | 66 | 14 | 613 | 14 | 2599.64 | 8.520288 | 23.52 | FW16_GLEAN_10013250 | FW16_GLEAN_10013250 | FW16_GLEAN_10013250 | FW16_GLEAN_10013250 | FW16_GLEAN_10013250 | FW16_GLEAN_10013250 | FW16_GLEAN_10013250 | FW16_GLEAN_10013250 | PROTEOLYSIS | SERINE PEPTIDASE |  |  |
| 423 | FW16_GLEAN_10013277 | 29.4118 | 4 | 4 | 4 | 11.6872 | 9.524929 | 17.85 | FW16_GLEAN_10013277 |  | FW16_GLEAN_10013277 |  |  | FW16_GLEAN_10013277 |  |  | OTHER BIOLOGICAL PROCESS | cell wall [Fusarium albosuccineum] |  |  |
| 424 | FW16_GLEAN_10013290 | 9 | 4 | 9 | 4 | 29.16 | 4.10532 | 68.39 |  |  | FW16_GLEAN_10013290 |  | FW16_GLEAN_10013290 | FW16_GLEAN_10013290 | FW16_GLEAN_10013290 | FW16_GLEAN_10013290 | LIPID METABOLIC | lysophospholipase |  |  |
| 425 | FW16_GLEAN_10013304 | 41 | 12 | 1561 | 12 | 5873.64 | 9.14945 | 22.08 | FW16_GLEAN_10013304 | FW16_GLEAN_10013304 | FW16_GLEAN_10013304 | FW16_GLEAN_10013304 | FW16_GLEAN_10013304 | FW16_GLEAN_10013304 | FW16_GLEAN_10013304 | FW16_GLEAN_10013304 | CARBOHYDRATE METABOLISM |  | GH11 | endo-β-1,4-xylanase / endo-β-1,3-xylanase |
| 426 | FW16_GLEAN_10013305 | 77 | 8 | 698 | 8 | 2621.2 | 8.169109 | 24.93 | FW16_GLEAN_10013305 | FW16_GLEAN_10013305 |  | FW16_GLEAN_10013305 | FW16_GLEAN_10013305 |  | FW16_GLEAN_10013305 | FW16_GLEAN_10013305 | CARBOHYDRATE METABOLISM |  | CE4 | acetyl xylan esterase / chitin deacetylase / chitooligosaccharide deacetylase |
| 427 | FW16_GLEAN_10013316 | 59 | 11 | 189 | 11 | 661.35 | 5.670811 | 24.02 |  |  |  |  |  |  | FW16_GLEAN_10013316 | FW16_GLEAN_10013316 | CARBOHYDRATE METABOLISM |  | CE12 | pectin acetylesterase/ rhamnogalacturonan acetylesterase / acetyl xylan esterase |
| 428 | FW16_GLEAN_10013337 | 25 | 7 | 67 | 7 | 265.54 | 4.230944 | 47.56 | FW16_GLEAN_10013337 |  | FW16_GLEAN_10013337 |  | FW16_GLEAN_10013337 |  | FW16_GLEAN_10013337 | FW16_GLEAN_10013337 | CARBOHYDRATE METABOLISM |  | GH17 | glucan endo-1,3-β-glucosidase |
| 429 | FW16_GLEAN_10013346 | 6 | 1 | 2 | 1 | 6.66 | 4.387072 | 25.23 | FW16_GLEAN_10013346 | FW16_GLEAN_10013346 | FW16_GLEAN_10013346 | FW16_GLEAN_10013346 | FW16_GLEAN_10013346 |  |  |  | CARBOHYDRATE METABOLISM |  | GH16 | Endo-1,3-1,4-beta-glycanase eglC [Fusarium oxysporum f. sp. cubense race 1] |
| 430 | FW16_GLEAN_10013360 | 38 | 10 | 51 | 10 | 211.15 | 5.718554 | 44.62 |  |  |  | FW16_GLEAN_10013360 |  |  | FW16_GLEAN_10013360 | FW16_GLEAN_10013360 | CARBOHYDRATE METABOLISM |  | AA1 | laccase 2 |
| 431 | FW16_GLEAN_10013411 | 4.56989 | 1 | 1 | 1 | 2.49205 | 5.903864 | 40.46 |  |  | FW16_GLEAN_10013411 |  |  |  |  |  | OTHER BIOLOGICAL PROCESS | peptidyl-prolyl isomerase D |  |  |
| 432 | FW16_GLEAN_10013507 | 50 | 6 | 23 | 6 | 60.1942 | 8.503056 | 13.21 | FW16_GLEAN_10013507 | FW16_GLEAN_10013507 | FW16_GLEAN_10013507 | FW16_GLEAN_10013507 | FW16_GLEAN_10013507 | FW16_GLEAN_10013507 |  |  | OTHER BIOLOGICAL PROCESS | rot1 PRECURSOR [Fusarium agapanthi] |  |  |
| 433 | FW16_GLEAN_10013550 | 12.8035 | 4 | 19 | 4 | 64.24 | 4.31553 | 47.68 | FW16_GLEAN_10013550 | FW16_GLEAN_10013550 | FW16_GLEAN_10013550 | FW16_GLEAN_10013550 | FW16_GLEAN_10013550 | FW16_GLEAN_10013550 | FW16_GLEAN_10013550 | FW16_GLEAN_10013550 | CARBOHYDRATE METABOLISM |  | GH72 | β-1,3-glucanosyltransglycosylase |
| 434 | FW16_GLEAN_10013556 | 1.87891 | 1 | 1 | 1 | 2.37759 | 9.914845 | 50.74 |  |  | FW16_GLEAN_10013556 |  | FW16_GLEAN_10013556 |  |  |  | PROTEIN BIOSYNTHESIS |  |  |  |
| 435 | FW16_GLEAN_10013562 | 58 | 15 | 147 | 15 | 570.38 | 4.186677 | 40.31 | FW16_GLEAN_10013562 | FW16_GLEAN_10013562 | FW16_GLEAN_10013562 | FW16_GLEAN_10013562 | FW16_GLEAN_10013562 | FW16_GLEAN_10013562 | FW16_GLEAN_10013562 | FW16_GLEAN_10013562 | OTHER BIOLOGICAL PROCESS | sporulation-specific SPS2 [Fusarium albosuccineum] |  |  |
| 436 | FW16_GLEAN_10013667 | 1.92308 | 1 | 1 | 1 | 2.37373 | 5.190744 | 70.57 |  |  |  |  | FW16_GLEAN_10013667 |  |  |  | PROTEOLYSIS | alanine aminopeptidase |  |  |
| 437 | FW16_GLEAN_10013688 | 33 | 3 | 25 | 3 | 73.38 | 7.112324 | 17.01 | FW16_GLEAN_10013688 | FW16_GLEAN_10013688 |  |  |  |  | FW16_GLEAN_10013688 | FW16_GLEAN_10013688 | NONE CONSERVED DOMAIN |  |  |  |
| 438 | FW16_GLEAN_10013698 | 16.7598 | 1 | 1 | 1 | 2.90782 | 4.629659 | 19.24 | FW16_GLEAN_10013698 |  |  |  |  |  |  |  | OXIDATION-REDUCTION | L-methionine (R)-S-oxide reductase |  |  |
| 439 | FW16_GLEAN_10013741 | 18 | 1 | 1 | 1 | 3.57 | 3.791979 | 15.76 | FW16_GLEAN_10013741 |  |  | FW16_GLEAN_10013741 |  |  |  |  | LIPID METABOLIC | phospholipase d1 [Fusarium albosuccineum] |  |  |
| 440 | FW16_GLEAN_10013842 | 4 | 1 | 2 | 1 | 5.29 | 5.33131 | 27.68 |  |  | FW16_GLEAN_10013842 |  |  |  |  |  | PROTEOLYSIS | Proteasome subunit |  |  |
| 441 | FW16_GLEAN_10013849 | 9.86395 | 1 | 1 | 1 | 4.67117 | 4.989715 | 33.45 |  |  | FW16_GLEAN_10013849 |  | FW16_GLEAN_10013849 |  |  |  | OTHER BIOLOGICAL PROCESS | Spermidine synthase |  |  |
| 442 | FW16_GLEAN_10013860 | 1.30058 | 1 | 1 | 1 | 1.81873 | 5.294043 | 75.37 |  |  | FW16_GLEAN_10013860 |  |  |  |  |  | OTHER BIOLOGICAL PROCESS | Transketolase |  |  |
| 443 | FW16_GLEAN_10013878 | 18 | 4 | 10 | 4 | 41.83 | 3.58223 | 38.60 |  |  |  |  |  |  | FW16_GLEAN_10013878 |  | CARBOHYDRATE METABOLISM |  | PL3-CBM1 | pectate lyase |
| 444 | FW16_GLEAN_10013922 | 0.608011 | 1 | 1 | 1 | 2.51371 | #NV | #NV |  |  | FW16_GLEAN_10013922 |  |  |  |  |  | PROTEIN BIOSYNTHESIS | translational activator |  |  |
| 445 | FW16_GLEAN_10013926 | 12.9747 | 3 | 3 | 3 | 9.91606 | 6.382108 | 35.10 | FW16_GLEAN_10013926 |  | FW16_GLEAN_10013926 | FW16_GLEAN_10013926 |  |  |  |  | RNA METABOLIC | Guanine nucleotide-binding protein subunit beta-like protein [Fusarium kuroshium] |  |  |
| 446 | FW16_GLEAN_10013972 | 3.51562 | 1 | 1 | 1 | 2.22129 | 8.303204 | 28.37 |  |  | FW16_GLEAN_10013972 |  |  |  |  |  | OTHER BIOLOGICAL PROCESS | adenylate kinase |  |  |
| 447 | FW16_GLEAN_10013988 | 2.52294 | 1 | 1 | 1 | 2.40637 | 5.350948 | 48.53 |  |  | FW16_GLEAN_10013988 |  |  |  |  |  | OTHER BIOLOGICAL PROCESS | adenylosuccinate synthetase |  |  |
| 448 | FW16_GLEAN_10014049 | 6.56716 | 2 | 2 | 2 | 4.75561 | 5.184955 | 37.97 | FW16_GLEAN_10014049 |  |  |  |  |  |  |  | OTHER BIOLOGICAL PROCESS | retinaldehyde binding/alpha-tocopherol transport |  |  |
| 449 | FW16_GLEAN_10014052 | 2.52525 | 1 | 1 | 1 | 2.51283 | 4.421485 | 41.30 |  |  | FW16_GLEAN_10014052 |  | FW16_GLEAN_10014052 | FW16_GLEAN_10014052 |  |  | PROTEOLYSIS | Aspartic peptidase |  |  |
| 450 | FW16_GLEAN_10014053 | 3.2345 | 1 | 1 | 1 | 0 | 4.850128 | 41.26 | FW16_GLEAN_10014053 |  |  |  |  |  |  |  | NONE CONSERVED DOMAIN |  |  |  |
| 451 | FW16_GLEAN_10014095 | 8.35509 | 6 | 6 | 6 | 15.2913 | 5.900777 | 86.26 | FW16_GLEAN_10014095 | FW16_GLEAN_10014095 | FW16_GLEAN_10014095 | FW16_GLEAN_10014095 |  | FW16_GLEAN_10014095 |  |  | AMINO ACID METABOLISM | metE 5-methyltetrahydropteroyltriglutamate--homocysteine methyltransferase |  |  |
| 452 | FW16_GLEAN_10014128 | 28.9773 | 6 | 8 | 6 | 26.6984 | 6.410114 | 37.05 | FW16_GLEAN_10014128 | FW16_GLEAN_10014128 | FW16_GLEAN_10014128 |  |  |  |  |  | OTHER BIOLOGICAL PROCESS | putative 5-methyltetrahydropteroyltriglutamate--homocysteine methyltransferase [Fusarium sp. AF-8] |  |  |
| 453 | FW16_GLEAN_10014145 | 20.9091 | 3 | 3 | 3 | 10.1325 | 5.800045 | 24.49 | FW16_GLEAN_10014145 | FW16_GLEAN_10014145 | FW16_GLEAN_10014145 | FW16_GLEAN_10014145 | FW16_GLEAN_10014145 | FW16_GLEAN_10014145 |  |  | OTHER BIOLOGICAL PROCESS | Small GTPase superfamily |  |  |
| 454 | FW16_GLEAN_10014176 | 2.33766 | 1 | 1 | 1 | 2.36571 | 6.064489 | 41.01 |  |  |  | FW16_GLEAN_10014176 |  |  |  |  | AMINO ACID METABOLISM | alanine-glyoxylate transaminase / serine-glyoxylate transaminase / serine-pyruvate transaminase |  |  |
| 455 | FW16_GLEAN_10014188 | 3.71622 | 1 | 1 | 1 | 2.33779 | 4.524513 | 31.74 |  |  | FW16_GLEAN_10014188 |  |  |  |  |  | PROTEIN BIOSYNTHESIS | 40S ribosomal protein |  |  |
| 456 | FW16_GLEAN_10014192 | 6.19718 | 2 | 2 | 2 | 6.44567 | 5.985441 | 38.26 |  |  | FW16_GLEAN_10014192 |  |  |  |  |  | OTHER BIOLOGICAL PROCESS | related to stomatin [Fusarium proliferatum ET1] |  |  |
| 457 | FW16_GLEAN_10014251 | 10 | 1 | 9 | 1 | 40.71 | 10.216 | 17.61 | FW16_GLEAN_10014251 | FW16_GLEAN_10014251 |  | FW16_GLEAN_10014251 |  |  |  |  | PROTEIN BIOSYNTHESIS | ubiquitin-40S ribosomal protein S31 |  |  |
| 458 | FW16_GLEAN_10014307 | 3 | 1 | 17 | 1 | 45.77 | 8.535686 | 55.25 | FW16_GLEAN_10014307 | FW16_GLEAN_10014307 | FW16_GLEAN_10014307 | FW16_GLEAN_10014307 | FW16_GLEAN_10014307 | FW16_GLEAN_10014307 |  |  | OTHER BIOLOGICAL PROCESS | integral component of membrane; cellular_component |  |  |
| 459 | FW16_GLEAN_10014313 | 12 | 6 | 36 | 6 | 109.85 | 4.828916 | 86.03 |  |  |  |  |  |  | FW16_GLEAN_10014313 | FW16_GLEAN_10014313 | PROTEOLYSIS | SERINE PEPTIDASE |  |  |
| 460 | FW16_GLEAN_10014327 | 15 | 4 | 17 | 4 | 55.64 | 9.159728 | 36.27 |  |  | FW16_GLEAN_10014327 |  |  |  |  |  | CARBOHYDRATE METABOLISM |  | GH53 | endo-β-1,4-galactanase |
| 461 | FW16_GLEAN_10014336 | 6.50407 | 3 | 3 | 3 | 2.48698 | 8.810644 | 51.11 | FW16_GLEAN_10014336 | FW16_GLEAN_10014336 | FW16_GLEAN_10014336 |  |  |  |  |  | CARBOHYDRATE METABOLISM |  | AA7 | glucooligosaccharide oxidase / chitooligosaccharide oxidase |
| 462 | FW16_GLEAN_10014384 | 7.72059 | 1 | 1 | 1 | 3.32406 | 5.434261 | 27.32 |  |  | FW16_GLEAN_10014384 |  |  |  |  |  | CARBOHYDRATE METABOLISM |  | AA9 | copper-dependent lytic polysaccharide monooxygenases |
| 463 | FW16_GLEAN_10014386 | 2.38095 | 1 | 1 | 1 | 2.538 | 5.054456 | 56.89 |  |  |  |  | FW16_GLEAN_10014386 |  |  |  | AMINO ACID METABOLISM | fungal specific transcription factor domain [Fusarium albosuccineum] |  |  |
| 464 | FW16_GLEAN_10014470 | 3.86965 | 2 | 2 | 2 | 4.45801 | 6.23088 | 52.62 | FW16_GLEAN_10014470 |  | FW16_GLEAN_10014470 | FW16_GLEAN_10014470 |  | FW16_GLEAN_10014470 |  |  | PROTEOLYSIS | Serine carboxypeptidase ARB |  |  |
| 465 | FW16_GLEAN_10014495 | 5 | 3 | 45 | 1 | 131.61 | 4.655967 | 49.62 | FW16_GLEAN_10014495 | FW16_GLEAN_10014495 | FW16_GLEAN_10014495 | FW16_GLEAN_10014495 | FW16_GLEAN_10014495 | FW16_GLEAN_10014495 |  |  | OTHER BIOLOGICAL PROCESS | tubulin beta |  |  |
| 466 | FW16_GLEAN_10014529 | 4.71545 | 1 | 1 | 1 | 3.77782 | 5.525875 | 69.72 | FW16_GLEAN_10014529 | FW16_GLEAN_10014529 | FW16_GLEAN_10014529 | FW16_GLEAN_10014529 | FW16_GLEAN_10014529 | FW16_GLEAN_10014529 |  |  | PROTEOLYSIS | Xaa-Pro dipeptidyl-peptidase-like domain |  |  |
| 467 | FW16_GLEAN_10014534 | 13 | 3 | 13 | 3 | 41.61 | 5.922411 | 28.53 | FW16_GLEAN_10014534 |  |  |  |  |  |  | FW16_GLEAN_10014534 | OTHER BIOLOGICAL PROCESS | PAN DOMAIN |  |  |
| 468 | FW16_GLEAN_10014717 | 10.9612 | 4 | 6 | 4 | 27.9771 | 5.656417 | 61.48 | FW16_GLEAN_10014717 | FW16_GLEAN_10014717 | FW16_GLEAN_10014717 | FW16_GLEAN_10014717 | FW16_GLEAN_10014717 | FW16_GLEAN_10014717 |  | FW16_GLEAN_10014717 | CARBOHYDRATE METABOLISM |  | GH71-CBM24-CBM24 | α-1,3-glucanase |
| 469 | FW16_GLEAN_10014718 | 19.4245 | 2 | 4 | 2 | 12.24 | 8.828472 | 12.70 | FW16_GLEAN_10014718 | FW16_GLEAN_10014718 |  | FW16_GLEAN_10014718 | FW16_GLEAN_10014718 | FW16_GLEAN_10014718 |  |  | OTHER BIOLOGICAL PROCESS | SnodProt1 |  |  |
| 470 | FW16_GLEAN_10014742 | 20.5298 | 4 | 5 | 4 | 17.2339 | 9.706884 | 30.99 | FW16_GLEAN_10014742 | FW16_GLEAN_10014742 | FW16_GLEAN_10014742 |  | FW16_GLEAN_10014742 |  |  |  | CARBOHYDRATE METABOLISM |  | GH17 | glucan endo-1,3-β-glucosidase |
| 471 | FW16_GLEAN_10014747 | 41.5584 | 5 | 9 | 5 | 25.054 | 8.685873 | 14.74 | FW16_GLEAN_10014747 | FW16_GLEAN_10014747 | FW16_GLEAN_10014747 | FW16_GLEAN_10014747 | FW16_GLEAN_10014747 | FW16_GLEAN_10014747 |  |  | NONE CONSERVED DOMAIN |  |  |  |
| 472 | FW16_GLEAN_10014771 | 9 | 6 | 12 | 6 | 39.29 | 5.701464 | 107.21 |  |  |  |  |  |  | FW16_GLEAN_10014771 |  | CARBOHYDRATE METABOLISM |  | GH31 | α-glucosidases |
| 473 | FW16_GLEAN_10014832 | 3 | 1 | 4 | 1 | 16.55 | 6.135529 | 55.90 | FW16_GLEAN_10014832 | FW16_GLEAN_10014832 | FW16_GLEAN_10014832 | FW16_GLEAN_10014832 | FW16_GLEAN_10014832 | FW16_GLEAN_10014832 |  | FW16_GLEAN_10014832 | CARBOHYDRATE METABOLISM |  | CE1 | feruloyl esterase |
| 474 | FW16_GLEAN_10014957 | 4 | 1 | 1 | 1 | 3.24 | 4.855844 | 41.02 | FW16_GLEAN_10014957 | FW16_GLEAN_10014957 | FW16_GLEAN_10014957 | FW16_GLEAN_10014957 | FW16_GLEAN_10014957 | FW16_GLEAN_10014957 |  |  | OTHER BIOLOGICAL PROCESS | gluconolactonase |  |  |
| 475 | FW16_GLEAN_10014963 | 3.76884 | 1 | 1 | 1 | 2.55303 | 5.573897 | 43.97 |  |  | FW16_GLEAN_10014963 |  |  |  |  |  | AMINO ACID METABOLISM | Aminotransferase class |  |  |
| 476 | FW16_GLEAN_10014974 | 5.69476 | 2 | 2 | 2 | 7.50609 | 4.012733 | 45.23 | FW16_GLEAN_10014974 |  |  | FW16_GLEAN_10014974 | FW16_GLEAN_10014974 |  |  |  | PROTEOLYSIS | Aspartic peptidase |  |  |
| 477 | FW16_GLEAN_10015029 | 24 | 3 | 15 | 3 | 47.89 | 5.147017 | 27.58 |  |  |  |  |  |  | FW16_GLEAN_10015029 |  | OTHER BIOLOGICAL PROCESS | ABHYDROLASE (alpha/beta hydrolase) |  |  |
| 478 | FW16_GLEAN_10015055 | 1 | 1 | 1 | 1 | 2.87 | 3.950055 | 105.31 | FW16_GLEAN_10015055 | FW16_GLEAN_10015055 | FW16_GLEAN_10015055 | FW16_GLEAN_10015055 | FW16_GLEAN_10015055 | FW16_GLEAN_10015055 | FW16_GLEAN_10015055 | FW16_GLEAN_10015055 | NONE CONSERVED DOMAIN | hypothetical protein CEP54_006446 [Fusarium sp. AF-8] |  |  |
| 479 | FW16_GLEAN_10015069 | 6.15385 | 1 | 1 | 1 | 3.96179 | 7.334641 | 35.77 |  |  |  |  |  | FW16_GLEAN_10015069 |  |  | NONE CONSERVED DOMAIN |  |  |  |
| 480 | FW16_GLEAN_10015125 | 7.24638 | 1 | 1 | 1 | 1.67463 | 8.197079 | 13.27 | FW16_GLEAN_10015125 | FW16_GLEAN_10015125 |  | FW16_GLEAN_10015125 |  |  | FW16_GLEAN_10015540 |  | NONE CONSERVED DOMAIN |  |  |  |
| 481 | FW16_GLEAN_10015150 | 6.81115 | 1 | 1 | 1 | 3.26158 | 5.124503 | 36.21 |  |  |  |  |  | FW16_GLEAN_10015150 |  |  | AMINO ACID METABOLISM | tryptophan catabolic process to kynurenine |  |  |
| 482 | FW16_GLEAN_10015175 | 28 | 7 | 82 | 7 | 323.08 | 4.765923 | 45.26 |  |  |  |  |  |  | FW16_GLEAN_10015175 |  | CARBOHYDRATE METABOLISM |  | GH5 | endo-β-1,4-glucanase / cellulase and other |
| 483 | FW16_GLEAN_10015177 | 42 | 4 | 57 | 4 | 281.75 | 4.130748 | 25.73 |  |  |  |  |  |  | FW16_GLEAN_10015177 | FW16_GLEAN_10015177 | RNA METABOLIC | RIBONUCLEASE T2 (RNAse T2) |  |  |
| 484 | FW16_GLEAN_10015193 | 17.5532 | 1 | 1 | 1 | 0 | 4.784792 | 17.71 | FW16_GLEAN_10015193 |  |  |  |  |  |  |  | OTHER BIOLOGICAL PROCESS | Cell wall protein |  |  |
| 485 | FW16_GLEAN_10015262 | 16.25 | 1 | 1 | 1 | 2.76 | 8.204319 | 6.50 |  |  |  |  |  | FW16_GLEAN_10015262 |  |  | NONE CONSERVED DOMAIN |  |  |  |
| 486 | FW16_GLEAN_10015284 | 4 | 1 | 2 | 1 | 7.04 | 4.655843 | 32.06 | FW16_GLEAN_10015284 | FW16_GLEAN_10015284 | FW16_GLEAN_10015284 | FW16_GLEAN_10015284 | FW16_GLEAN_10015284 | FW16_GLEAN_10015284 | FW16_GLEAN_10015284 | FW16_GLEAN_10015284 | NONE CONSERVED DOMAIN | NECHADRAFT_45430 [Fusarium vanettenii 77-13-4] |  |  |
| 487 | FW16_GLEAN_10015303 | 46 | 9 | 85 | 9 | 350.83 | 8.299212 | 34.89 | FW16_GLEAN_10015303 | FW16_GLEAN_10015303 | FW16_GLEAN_10015303 | FW16_GLEAN_10015303 | FW16_GLEAN_10015303 | FW16_GLEAN_10015303 | FW16_GLEAN_10015303 |  | NONE CONSERVED DOMAIN |  |  |  |
| 488 | FW16_GLEAN_10015374 | 28 | 2 | 15 | 2 | 63.15 | 5.075172 | 19.28 | FW16_GLEAN_10015374 | FW16_GLEAN_10015374 | FW16_GLEAN_10015374 | FW16_GLEAN_10015374 | FW16_GLEAN_10015374 |  |  |  | NONE CONSERVED DOMAIN |  |  |  |
| 489 | FW16_GLEAN_10015384 | 10 | 3 | 10 | 3 | 31.65 | 6.176745 | 72.04 |  |  |  |  |  |  | FW16_GLEAN_10015384 |  | CARBOHYDRATE METABOLISM |  | PL4 | Rhamnogalacturonan lyase |
| 490 | FW16_GLEAN_10015397 | 6 | 1 | 9 | 1 | 28.63 | 4.053547 | 17.79 | FW16_GLEAN_10015397 | FW16_GLEAN_10015397 | FW16_GLEAN_10015397 | FW16_GLEAN_10015397 | FW16_GLEAN_10015397 | FW16_GLEAN_10015397 | FW16_GLEAN_10015397 |  | OTHER BIOLOGICAL PROCESS | Extracellular membrane protein |  |  |
| 491 | FW16_GLEAN_10015405 | 5.12 | 2 | 2 | 2 | 7.41 | 8.258373 | 65.56 | FW16_GLEAN_10015405 | FW16_GLEAN_10015405 | FW16_GLEAN_10015405 |  | FW16_GLEAN_10015405 | FW16_GLEAN_10015405 |  |  | OXIDATION-REDUCTION | 2-polyprenyl-6-methoxyphenol hydroxylase and related FAD-dependent oxidoreductases |  | |
| 492 | FW16_GLEAN_10015441 | 5.15021 | 1 | 1 | 1 | 0 | 6.24748 | 50.28 | FW16_GLEAN_10015441 | FW16_GLEAN_10015441 | FW16_GLEAN_10015441 | FW16_GLEAN_10015441 | FW16_GLEAN_10015441 | FW16_GLEAN_10015441 | FW16_GLEAN_10015441 | FW16_GLEAN_10015441 | RNA METABOLIC | NACHT nucleoside triphosphatase |  |  |
| 493 | FW16_GLEAN_10015477 | 3.37302 | 2 | 2 | 2 | 4.09891 | 4.997823 | 54.50 |  |  |  | FW16_GLEAN_10015477 |  |  |  |  | NONE CONSERVED DOMAIN |  |  |  |
| 494 | FW16_GLEAN_10015489 | 53 | 12 | 319 | 12 | 1308.87 | 5.267673 | 44.75 |  |  |  |  |  |  | FW16_GLEAN_10015489 | FW16_GLEAN_10015489 | PROTEOLYSIS | METALLOCARBOXIPEPTIDASE |  |  |
| 495 | FW16_GLEAN_10015496 | 23.3898 | 6 | 13 | 6 | 44.79 | 5.478397 | 29.82 | FW16_GLEAN_10015496 | FW16_GLEAN_10015496 | FW16_GLEAN_10015496 | FW16_GLEAN_10015496 |  | FW16_GLEAN_10015496 |  |  | CARBOHYDRATE METABOLISM |  | CE16 | acetylesterase |
| 496 | FW16_GLEAN_10015497 | 26.2376 | 8 | 15 | 7 | 45.3202 | 8.301765 | 43.94 | FW16_GLEAN_10015497 | FW16_GLEAN_10015497 | FW16_GLEAN_10015497 |  |  |  |  |  | CARBOHYDRATE METABOLISM |  | GH5 | endo-β-1,4-glucanase / cellulase and other |
| 497 | FW16_GLEAN_10015530 | 17 | 3 | 8 | 3 | 33.93 | 4.814323 | 23.35 | FW16_GLEAN_10015530 | FW16_GLEAN_10015530 | FW16_GLEAN_10015530 |  | FW16_GLEAN_10015530 | FW16_GLEAN_10015530 |  |  | CARBOHYDRATE METABOLISM |  | CBM9 |  |
| 498 | FW16_GLEAN_10015540 | 5 | 2 | 4 | 2 | 11.59 | 6.055947 | 59.17 | FW16_GLEAN_10015540 | FW16_GLEAN_10015540 | FW16_GLEAN_10015540 | FW16_GLEAN_10015540 | FW16_GLEAN_10015540 | FW16_GLEAN_10015540 |  |  | NONE CONSERVED DOMAIN | hypothetical protein CDV36_007838 [Fusarium kuroshium] |  |  |
| 499 | FW16_GLEAN_10015541 | 2.19199 | 1 | 1 | 1 | 0 | 6.994508 | 149.34 | FW16_GLEAN_10015541 |  |  | FW16_GLEAN_10015441 |  |  |  |  | LIPID METABOLIC | patatin-like serine protein [Fusarium austroafricanum] |  |  |
| 500 | FW16_GLEAN_10015547 | 2.62009 | 1 | 1 | 1 | 2.77303 | 6.599775 | 50.73 | FW16_GLEAN_10015547 |  |  | FW16_GLEAN_10015547 |  |  |  |  | OXIDATION-REDUCTION | FAD dependent oxidoreductase |  |  |

## Supplementary Table 4: Functional prediction of the CAZymes found on different synthetic and artificial cellulose and biomass substrates.

| Name | Number | Family | Predicted molecular function/Possible substrate(s) | top_1 | top_10 | top_100 | top_99999 | DIAMOND |
| --- | --- | --- | --- | --- | --- | --- | --- | --- |
| FW16_GLEAN_10001275 | 1 | AA1 | Laccase / Laccase-like multicopper oxidase |  |  |  |  | AA1_3 |
| FW16_GLEAN_10013360 | 2 | AA1 | Laccase / Laccase-like multicopper oxidase |  |  |  | EC 1.10.3.2 | AA1_3 |
| FW16_GLEAN_10001928 | 3 | AA2 | Manganese peroxidase / versatile peroxidase / lignin peroxidase |  |  |  |  | AA2 |
| FW16_GLEAN_10000205 | 4 | AA3 | Alcohol oxidase |  |  |  | 1.1.3.4\|1.1.3.7 |  |
| FW16_GLEAN_10000721 | 5 | AA3 | Cellobiose dehydrogenase |  |  |  | 1.1.3.13\|1.1.3.16\|1.1.3.4\|1.1.3.7 |  |
| FW16_GLEAN_10000164 | 6 | AA5 | Glyoxal oxidase |  |  |  | 1.1.3.-\|1.1.3.9\|1.2.3.15\|2.4.2.26\|3.2.1.58 |  |
| FW16_GLEAN_10006436 | 7 | AA6 | 1,4-Benzoquinone reductase |  |  | 1.6.5.6 | 1.6.5.6 |  |
| FW16_GLEAN_10008842 | 8 | AA7 | Glucooligosaccharide oxidase / chitooligosaccharide oxidase |  |  | 1.1.3.- | 1.1.3.- |  |
| FW16_GLEAN_10004754 | 9 | AA7 | Glucooligosaccharide oxidase / chitooligosaccharide oxidase |  |  | 1.1.3.- | 1.1.3.- |  |
| FW16_GLEAN_10014336 | 10 | AA7 | Glucooligosaccharide oxidase / chitooligosaccharide oxidase |  | 1.1.3.- | 1.1.3.- | 1.1.3.- |  |
| FW16_GLEAN_10012260 | 11 | AA7 | Glucooligosaccharide oxidase / chitooligosaccharide oxidase |  | 1.1.3.- | 1.1.3.- | 1.1.3.- |  |
| FW16_GLEAN_10001346 | 12 | AA9 | Copper-dependent lytic polysaccharide monooxygenases (LPMO) |  |  |  |  | AA9 |
| FW16_GLEAN_10014384 | 13 | AA9 | Copper-dependent lytic polysaccharide monooxygenases (LPMO) |  |  |  |  | AA9 |
| FW16_GLEAN_10007711 | 14 | AA9 | Copper-dependent lytic polysaccharide monooxygenases (LPMO) |  |  |  |  | AA9 |
| FW16_GLEAN_10005570 | 15 | AA11 | Copper-dependent lytic polysaccharide monooxygenases (LPMO) |  |  |  |  | AA11 |
| FW16_GLEAN_10004602 | 16 | AA9;CBM1 | Copper-dependent lytic polysaccharide monooxygenases (LPMO) |  |  |  | 3.2.1.132\|3.2.1.176 |  |
| FW16_GLEAN_10002187 | 17 | AA13;CBM20 | Copper-dependent lytic polysaccharide monooxygenases (LPMO) |  |  | 3.2.1.1\|3.2.1.3 | 2.4.1.19\|3.2.1.-\|3.2.1.1\|3.2.1.116\|3.2.1.2\|3.2.1.3 |  |
| FW16_GLEAN_10015530 | 18 | CBM9 | Modules binding xylan/ cellulose-binding function has been demonstrated in one case. |  |  |  |  | CBM9 |
| FW16_GLEAN_10000334 | 19 | CBM13 | Multivalent sugar-binding sites, as demonstrated for their interaction with xylan and arabinoxylan |  |  |  |  | CBM13 |
| FW16_GLEAN_10007143 | 20 | CBM63 | Bind cellulose |  |  |  | 3.2.1.4 | CBM63 |
| FW16_GLEAN_10004777 | 21 | CE1 | Xylan esterase |  | 3.1.1.72 | 3.1.1.-\|3.1.1.72 | 3.1.1.-\|3.1.1.6\|3.1.1.72\|3.1.1.73 |  |
| FW16_GLEAN_10014832 | 22 | CE1 | Feruloyl esterase |  |  |  |  | E3.1.1.73 |
| FW16_GLEAN_10001089 | 23 | CE2 | Acetyl xylan esterase |  |  | 3.1.1.-\|3.1.1.72\|3.2.1.4 | 3.1.1.-\|3.1.1.72\|3.2.1.4 |  |
| FW16_GLEAN_10013305 | 24 | CE4 | Acetyl xylan esterase / chitin deacetylase |  |  | 3.1.1.72\|3.5.1.41 | 3.1.1.72\|3.5.1.41 |  |
| FW16_GLEAN_10006900 | 25 | CE5 | Cutinase |  | 3.1.1.74 | 3.1.1.74 | 3.1.1.3\|3.1.1.74 |  |
| FW16_GLEAN_10007169 | 26 | CE5 | Acetyl xylan esterase |  |  | 3.1.1.72 | 3.1.1.72 |  |
| FW16_GLEAN_10001547 | 27 | CE8 | Pectin methylesterase |  |  | 3.1.1.11 | 3.1.1.11 |  |
| FW16_GLEAN_10001601 | 28 | CE8 | Pectin methylesterase |  |  | 3.1.1.11 | 3.1.1.11 |  |
| FW16_GLEAN_10011996 | 29 | CE8 | Pectin methylesterase |  |  |  | 3.1.1.11 |  |
| FW16_GLEAN_10012229 | 30 | CE12 | Pectin acetylesterase/ rhamnogalacturonan acetylesterase |  |  | 3.1.1.- | 3.1.1.- |  |
| FW16_GLEAN_10013316 | 31 | CE12 | Pectin acetylesterase/ rhamnogalacturonan acetylesterase |  |  |  |  | CE12 |
| FW16_GLEAN_10015496 | 32 | CE16 | Acetylesterase |  | 3.1.1.6 | 3.1.1.6 | 3.1.1.6 |  |
| FW16_GLEAN_10003711 | 33 | GH1 | β-glucosidase |  | 3.2.1.21 | 3.2.1.21 | 3.2.1.117\|3.2.1.118\|3.2.1.119\|3.2.1.125\|3.2.1.147\|3.2.1.149\|3.2.1.161\|3.2.1.182\|3.2.1.21\|3.2.1.25\|3.2.1.38 |  |
| FW16_GLEAN_10006734 | 34 | GH1 | β-glucosidase |  |  | 3.2.1.-\|3.2.1.21\|3.2.1.23\|3.2.1.37\|3.2.1.38 | 2.4.1.-\|3.2.1.-\|3.2.1.105\|3.2.1.108\|3.2.1.118\|3.2.1.147\|3.2.1.161\|3.2.1.21\|3.2.1.23\|3.2.1.37\|3.2.1.38\|3.2.1.62\|3.2.1.86 |  |
| FW16_GLEAN_10010580 | 35 | GH2 | β-mannosidase |  | 3.2.1.25 | 3.2.1.25 | 3.2.1.25 |  |
| FW16_GLEAN_10000066 | 36 | GH2 | β-galactosidase |  |  | 3.2.1.23 | 3.2.1.23 |  |
| FW16_GLEAN_10003498 | 37 | GH3 | β-glucosidase |  |  | 3.2.1.-\|3.2.1.21 | 3.2.1.-\|3.2.1.21\|3.2.1.37 |  |
| FW16_GLEAN_10008834 | 38 | GH3 | β-glucosidase |  | 3.2.1.21 | 3.2.1.-\|3.2.1.21 | 3.2.1.-\|3.2.1.21\|3.2.1.37 |  |
| FW16_GLEAN_10011639 | 39 | GH3 | β-glucosidase /xylan 1,4-β-xylosidase |  |  | 3.2.1.21\|3.2.1.37 | 3.2.1.21\|3.2.1.37\|3.2.1.55 |  |
| FW16_GLEAN_10011883 | 40 | GH3 | β-glucosidase |  |  | 3.2.1.21 | 3.2.1.-\|3.2.1.21\|3.2.1.37 |  |
| FW16_GLEAN_10004843 | 41 | GH3 | β-glucosidase |  |  | 3.2.1.21 | 3.2.1.-\|3.2.1.21\|3.2.1.37 |  |
| FW16_GLEAN_10001962 | 42 | GH5 | Endo-β-1,4-glucanase / cellulase / other |  |  | 4.2.2.- | 4.2.2.- |  |
| FW16_GLEAN_10004691 | 43 | GH5 | glucan endo-1,6-β-glucosidase |  | 3.2.1.75 | 3.2.1.21\|3.2.1.55\|3.2.1.75 | 3.2.1.-\|3.2.1.21\|3.2.1.55\|3.2.1.58\|3.2.1.75 |  |
| FW16_GLEAN_10015175 | 44 | GH5 | endo-β-1,6-galactanase |  | 3.2.1.164 | 3.2.1.164 | 3.2.1.164\|3.2.1.4 |  |
| FW16_GLEAN_10015497 | 45 | GH5 | mannan endo-β-1,4-mannosidase |  | 3.2.1.78 | 3.2.1.78 | 2.4.1.-\|3.2.1.78 |  |
| FW16_GLEAN_10007085 | 46 | GH7 | Endo-β-1,4-glucanase |  | 3.2.1.4 | 3.2.1.176\|3.2.1.4\|3.2.1.73 | 3.2.1.132\|3.2.1.176\|3.2.1.4\|3.2.1.73 |  |
| FW16_GLEAN_10003305 | 47 | GH10 | Endo-1,4-β-xylanase |  | 3.2.1.8 | 3.2.1.8 | 3.2.1.8 |  |
| FW16_GLEAN_10001573 | 48 | GH10 | Endo-1,4-β-xylanase | 3.2.1.8 | 3.2.1.8 | 3.2.1.8 | 3.1.1.73\|3.2.1.8 |  |
| FW16_GLEAN_10013304 | 49 | GH11 | Endo-β-1,4-xylanase | 3.2.1.8 | 3.2.1.8 | 3.2.1.8 | 3.2.1.8 |  |
| FW16_GLEAN_10001797 | 50 | GH12 | Xyloglucan hydrolase |  | 3.2.1.151\|3.2.1.4 | 3.2.1.151\|3.2.1.4\|3.2.1.73 | 3.2.1.151\|3.2.1.4\|3.2.1.73 |  |
| FW16_GLEAN_10008963 | 51 | GH12 | Xyloglucan hydrolase | 3.2.1.4 | 3.2.1.151\|3.2.1.4 | 3.2.1.151\|3.2.1.4\|3.2.1.73 | 3.2.1.151\|3.2.1.4\|3.2.1.73 |  |
| FW16_GLEAN_10001440 | 52 | GH13 | α-Amylase |  |  | 3.2.1.1 | 2.4.1.-\|2.4.1.19\|2.4.1.25\|3.2.1.1\|3.2.1.116\|3.2.1.133\|3.2.1.2\|3.2.1.41 |  |
| FW16_GLEAN_10009774 | 53 | GH16 | Endo-β-1,4-galactosidase / chitin β-1,6-glucanosyltransferase / β-transglycosidase / β-glycosidase / β-carrageenase/ endo-1,3-β-glucanase / laminarinase/ licheninase |  |  | 2.4.1.-\|3.2.1.-\|3.2.1.39\|3.2.1.6\|3.2.1.73 | 2.4.1.-\|3.2.1.-\|3.2.1.35\|3.2.1.39\|3.2.1.6\|3.2.1.73 |  |
| FW16_GLEAN_10010201 | 54 | GH16 | Chitin β-1,6-glucanosyltransferase / β-transglycosidase / endo-1,3-β-glucanase / laminarinase |  |  | 2.4.1.-\|3.2.1.39 | 2.4.1.-\|3.2.1.-\|3.2.1.39 |  |
| FW16_GLEAN_10003917 | 55 | GH16 | Endo-β-1,4-galactosidase / chitin β-1,6-glucanosyltransferase / β-transglycosidase / β-glycosidase / β-carrageenase/ endo-1,3-β-glucanase / laminarinase/ licheninase |  |  | 2.4.1.-\|3.2.1.-\|3.2.1.39\|3.2.1.6\|3.2.1.73 | 2.4.1.-\|3.2.1.-\|3.2.1.35\|3.2.1.39\|3.2.1.6\|3.2.1.73 |  |
| FW16_GLEAN_10009225 | 56 | GH16 | Chitin β-1,6-glucanosyltransferase / β-transglycosidase / endo-1,3-β-glucanase / laminarinase |  |  | 2.4.1.-\|3.2.1.39 | 2.4.1.-\|3.2.1.-\|3.2.1.39 |  |
| FW16_GLEAN_10013346 | 57 | GH16 | Endo-β-1,3-galactanase |  | 3.2.1.181 | 3.2.1.181 | 3.2.1.-\|3.2.1.181 |  |
| FW16_GLEAN_10004522 | 58 | GH17 | Glucan endo-1,3-β-glucosidase / licheninase / ABA-specific β-glucosidase / β-1,3-glucanosyltransglycosylase /β-1,3-glucosidase |  |  |  |  | GH17 unknown |
| FW16_GLEAN_10010890 | 59 | GH17 | β-1,3-Glucanosyltransglycosylase / β-1,3-glucosidase |  |  | 2.4.1.-\|3.2.1.- | 2.4.1.-\|3.2.1.- |  |
| FW16_GLEAN_10012536 | 60 | GH17 | β-1,3-glucanosyltransglycosylase / β-1,3-glucosidase |  |  |  | 2.4.1.-\|3.2.1.- |  |
| FW16_GLEAN_10013337 | 61 | GH17 | β-1,3-Glucanosyltransglycosylase / β-1,3-glucosidase |  |  |  | 2.4.1.-\|3.2.1.- |  |
| FW16_GLEAN_10014742 | 62 | GH17 | β-1,3-Glucosidase |  |  | 3.2.1.- | 2.4.1.-\|3.2.1.- |  |
| FW16_GLEAN_10011231 | 63 | GH18 | Chitinase |  |  | 3.2.1.14 | 3.2.1.1\|3.2.1.14\|3.2.1.17\|3.2.1.41 |  |
| FW16_GLEAN_10006294 | 64 | GH20 | β-Hexosaminidase |  | 3.2.1.52 | 3.2.1.52 | 3.2.1.52 |  |
| FW16_GLEAN_10007529 | 65 | GH24 | Lysozyme |  |  |  |  | [3.2.1.17](http://www.enzyme-database.org/query.php?ec=3.2.1.17) |
| FW16_GLEAN_10000319 | 66 | GH28 | Exo-polygalacturonase / xylogalacturonan hydrolase |  |  | 3.2.1.-\|3.2.1.67 | 3.2.1.-\|3.2.1.15\|3.2.1.67 |  |
| FW16_GLEAN_10001538 | 67 | GH28 | Exo-polygalacturonase |  |  |  |  | E3.2.1.67 |
| FW16_GLEAN_10005091 | 68 | GH28 | Exo-polygalacturonase | 3.2.1.67 | 3.2.1.67 | 3.2.1.-\|3.2.1.67 | 3.2.1.-\|3.2.1.15\|3.2.1.67 |  |
| FW16_GLEAN_10008243 | 69 | GH28 | Exo-polygalacturonase |  |  | 3.2.1.67 | 3.2.1.-\|3.2.1.15\|3.2.1.67 |  |
| FW16_GLEAN_10014771 | 70 | GH31 | α-glucosidases |  | 3.2.1.20 | 3.2.1.20\|3.2.1.84 | 3.2.1.10\|3.2.1.177\|3.2.1.20\|3.2.1.48\|3.2.1.84 |  |
| FW16_GLEAN_10011615 | 71 | GH32 | Fructan:fructan 1-fructosyltransferase |  | 2.4.1.100\|3.2.1.26 | 2.4.1.-\|2.4.1.100\|3.2.1.26 | 2.4.1.-\|2.4.1.100\|3.2.1.26 |  |
| FW16_GLEAN_10000618 | 72 | GH35 | β-Galactosidase / β-1,3-galactosidase |  |  | 2.4.1.-\|3.2.1.-\|3.2.1.23 | 2.4.1.-\|3.2.1.-\|3.2.1.23 |  |
| FW16_GLEAN_10002565 | 73 | GH37 | α,α-Trehalase |  |  | 3.2.1.28 | 3.2.1.28 |  |
| FW16_GLEAN_10003104 | 74 | GH39 | β-Glucosidase / β-galactosidase/ β-xylosidase / α-L-arabinofuranosidase |  |  |  | 3.2.1.21\|3.2.1.23\|3.2.1.37\|3.2.1.55 |  |
| FW16_GLEAN_10001518 | 75 | GH43 | β-Xylosidase / α-L-arabinofuranosidase |  |  |  | 3.2.1.146\|3.2.1.55 |  |
| FW16_GLEAN_10000829 | 76 | GH43 | α-1,2-L-Arabinofuranosidase / exo-α-1,5-L-arabinofuranosidase / exo-α-1,5-L-arabinanase / β-1,3-xylosidase /exo-α-1,5-L-arabinanase / β-xylosidase |  |  |  | 3.2.1.-\|3.2.1.37\|3.2.1.55 |  |
| FW16_GLEAN_10003286 | 77 | GH43 | Endo-α-1,5-L-arabinanase |  |  |  | 3.2.1.99 |  |
| FW16_GLEAN_10006822 | 78 | GH43 | β-Xylosidase / α-L-arabinofuranosidase |  |  |  | 3.2.1.37\|3.2.1.55 |  |
| FW16_GLEAN_10007175 | 79 | GH43 | β-D-Galactofuranosidase |  |  | 3.2.1.146 | 3.2.1.146 |  |
| FW16_GLEAN_10009840 | 80 | GH43 | α-L-Arabinofuranosidase |  |  | 3.2.1.55 | 3.2.1.37\|3.2.1.55 |  |
| FW16_GLEAN_10010955 | 81 | GH43 | β-Xylosidase |  | 3.2.1.37 | 3.2.1.37\|3.2.1.55 | 3.2.1.37\|3.2.1.55 |  |
| FW16_GLEAN_10001821 | 82 | GH43 | α-1,2-L-Arabinofuranosidase / exo-α-1,5-L-arabinofuranosidase / exo-α-1,5-L-arabinanase / β-1,3-xylosidase /exo-α-1,5-L-arabinanase / α-L-arabinofuranosidase |  |  | 3.2.1.-\|3.2.1.55 | 3.2.1.-\|3.2.1.55 |  |
| FW16_GLEAN_10011918 | 83 | GH43 | α-1,2-L-Arabinofuranosidase / exo-α-1,5-L-arabinofuranosidase / exo-α-1,5-L-arabinanase / β-1,3-xylosidase /exo-α-1,5-L-arabinanase / α-L-arabinofuranosidase |  | 3.2.1.- | 3.2.1.-\|3.2.1.55 | 3.2.1.-\|3.2.1.55 |  |
| FW16_GLEAN_10012038 | 84 | GH43 | β-Xylosidase / α-L-arabinofuranosidase |  |  |  | 3.2.1.37\|3.2.1.55 |  |
| FW16_GLEAN_10012117 | 85 | GH49 | Dextranase |  | 3.2.1.11 | 3.2.1.-\|3.2.1.11\|3.2.1.57\|3.2.1.95 | 3.2.1.-\|3.2.1.11\|3.2.1.57\|3.2.1.95 |  |
| FW16_GLEAN_10003329 | 86 | GH51 | α-L-Arabinofuranosidase |  |  | 3.2.1.55 | 3.2.1.37\|3.2.1.55 |  |
| FW16_GLEAN_10014327 | 87 | GH53 | Endo-β-1,4-galactanase |  |  | 3.2.1.89 | 3.2.1.89 |  |
| FW16_GLEAN_10002430 | 88 | GH54 | α-L-Arabinofuranosidase | 3.2.1.55 | 3.2.1.37\|3.2.1.55 | 3.2.1.37\|3.2.1.55 | 3.2.1.146\|3.2.1.20\|3.2.1.37\|3.2.1.55 |  |
| FW16_GLEAN_10003512 | 89 | GH55 | Exo-β-1,3-glucanase |  |  | 3.2.1.58 | 3.2.1.39\|3.2.1.58 |  |
| FW16_GLEAN_10008698 | 90 | GH71 | α-1,3-Glucanase |  |  |  |  | [EC 3.2.1.59)](http://www.enzyme-database.org/query.php?ec=3.2.1.59) |
| FW16_GLEAN_10004186 | 91 | GH72 | β-1,3-Glucanosyltransglycosylase |  |  | 2.4.1.- | 2.4.1.- |  |
| FW16_GLEAN_10013550 | 92 | GH72 | β-1,3-Glucanosyltransglycosylase |  |  | 2.4.1.- | 2.4.1.- |  |
| FW16_GLEAN_10000631 | 93 | GH74 | Xyloglucanase |  | 3.2.1.151 | 3.2.1.151 | 3.2.1.151\|3.2.1.4 |  |
| FW16_GLEAN_10007311 | 94 | GH75 | Chitosanase | 3.2.1.132 | 3.2.1.132 | 3.2.1.132 | 3.2.1.132 |  |
| FW16_GLEAN_10004573 | 95 | GH79 | β-Glucuronidase /β-4-O-methyl-glucuronidase |  |  | 3.2.1.-\|3.2.1.31 | 3.2.1.-\|3.2.1.166\|3.2.1.31 |  |
| FW16_GLEAN_10011474 | 96 | GH81 | Endo-β-1,3-glucanase |  |  | 3.2.1.39 | 3.2.1.39 |  |
| FW16_GLEAN_10001805 | 97 | GH93 | Exo-α-L-1,5-arabinanase |  | 3.2.1.- | 3.2.1.- | 3.2.1.-\|3.2.1.151 |  |
| FW16_GLEAN_10011917 | 98 | GH93 | exo-α-L-1,5-arabinanase |  |  | 3.2.1.- | 2.4.1.99\|3.2.1.-\|3.2.1.151 |  |
| FW16_GLEAN_10012182 | 99 | GH99 | Glycoprotein endo-α-1,2-mannosidase |  |  | 3.2.1.130\|3.2.1.198 | 3.2.1.130\|3.2.1.198 |  |
| FW16_GLEAN_10003277 | 100 | GH115 | xylan α-1,2-glucuronidase |  |  | 3.2.1.131 | 3.2.1.131 |  |
| FW16_GLEAN_10001958 | 101 | GH115 | xylan α-1,2-glucuronidase |  |  | 3.2.1.131 | 3.2.1.131 |  |
| FW16_GLEAN_10005637 | 102 | GH128 | β-1,3-glucanase / β-1,3-glucosidase |  |  | 3.2.1.-\|3.2.1.39 | 3.2.1.-\|3.2.1.39 |  |
| FW16_GLEAN_10006366 | 103 | GH132 | Glycosidases maybe activity on β-1,3-glucan |  |  | 3.2.1.- | 3.2.1.- |  |
| FW16_GLEAN_10010582 | 104 | GH134 | Endo-β-1,4-mannanase |  |  | 3.2.1.78 | 3.2.1.78 |  |
| FW16_GLEAN_10000830 | 105 | GH146 | β-L-Arabinofuranosidase |  |  |  |  | [EC 3.2.1.185](http://www.enzyme-database.org/query.php?ec=3.2.1.185) |
| FW16_GLEAN_10000416 | 106 | GH5;CBM1 | Endo-β-1,4-glucanase / cellulase |  | 3.2.1.4 | 3.2.1.4 | 3.2.1.4 |  |
| FW16_GLEAN_10004889 | 107 | GH5;CBM1 | Mannan endo-β-1,4-mannosidase |  | 3.2.1.78 | 3.2.1.78 | 2.4.1.-\|3.2.1.78 |  |
| FW16_GLEAN_10006835 | 108 | GH6;CBM1 | Cellobiohydrolase |  | 3.2.1.91 | 3.2.1.4\|3.2.1.91 | 3.2.1.-\|3.2.1.4\|3.2.1.73\|3.2.1.8\|3.2.1.91 |  |
| FW16_GLEAN_10001888 | 109 | GH7;CBM1 | Reducing end-acting cellobiohydrolase |  |  | 3.2.1.176 | 3.2.1.132\|3.2.1.176\|3.2.1.4\|3.2.1.73 |  |
| FW16_GLEAN_10005918 | 110 | GH7;CBM1 | Reducing end-acting cellobiohydrolase/ chitosanase |  |  | 3.2.1.132\|3.2.1.176 | 3.2.1.132\|3.2.1.176\|3.2.1.4\|3.2.1.73 |  |
| FW16_GLEAN_10010356 | 111 | GH11;CBM1 | Endo-β-1,4-xylanase |  | 3.2.1.8 | 3.2.1.8 | 3.2.1.8 |  |
| FW16_GLEAN_10002866 | 112 | GH13;CBM48 | 1,4-α-Glucan branching enzyme |  |  | 2.4.1.18 | 2.4.1.18 |  |
| FW16_GLEAN_10004332 | 113 | GH15;CBM20 | Glucoamylase |  |  | 3.2.1.3 | 3.2.1.1\|3.2.1.3 |  |
| FW16_GLEAN_10007569 | 114 | GH16;CBM18 | Chitin β-1,6-glucanosyltransferase / β-transglycosidase / β-glycosidase / β-carrageenase /endo-1,3-β-glucanase / laminarinase |  |  |  | 2.4.1.-\|3.2.1.-\|3.2.1.39 |  |
| FW16_GLEAN_10008961 | 115 | GH18;CBM18 | Chitinase |  |  | 3.2.1.14 | 3.2.1.14 |  |
| FW16_GLEAN_10002083 | 116 | GH18;CBM18;CBM50 | Chitinase |  |  | 3.2.1.14 | 3.2.1.14 |  |
| FW16_GLEAN_10008188 | 117 | GH18;CBM18;CBM50;CBM50 | Chitinase |  |  |  | 3.2.1.-\|3.2.1.14 |  |
| FW16_GLEAN_10001593 | 118 | GH27;CBM35 | α-Galactosidase / β-L-arabinopyranosidase |  | 3.2.1.22\|3.2.1.88 | 3.2.1.22\|3.2.1.88 | 2.4.1.-\|3.2.1.22\|3.2.1.88 |  |
| FW16_GLEAN_10000952 | 119 | GH43;CBM35 | β-Xylosidase (Sub family 24) |  | 3.2.1.145 | 3.2.1.145 | 3.2.1.145 |  |
| FW16_GLEAN_10007861 | 120 | GH45;CBM1 | Endoglucanase |  |  | 3.2.1.4 | 3.2.1.151\|3.2.1.4\|3.2.1.78\|3.2.1.8\|3.2.1.91 |  |
| FW16_GLEAN_10014717 | 121 | GH71;CBM24;CBM24 | α-1,3-Glucanase |  | 3.2.1.59 | 3.2.1.59 | 3.2.1.59 |  |
| FW16_GLEAN_10012817 | 122 | GH72;CBM43 | β-1,3-Glucanosyltransglycosylase |  |  | 2.4.1.- | 2.4.1.- |  |
| FW16_GLEAN_10004549 | 123 | GT20 | Trehalose 6-phosphatase |  |  | 3.1.3.12 | 2.4.1.15\|3.1.3.12 |  |
| FW16_GLEAN_10001028 | 124 | PL1 | Pectate lyase |  | 4.2.2.2 | 4.2.2.2 | 4.2.2.2\|4.2.2.9 |  |
| FW16_GLEAN_10001932 | 125 | PL1 | Pectate lyase |  |  | 4.2.2.2 | 4.2.2.2\|4.2.2.9 |  |
| FW16_GLEAN_10012037 | 126 | PL1 | Pectate lyase |  |  | 4.2.2.2 | 4.2.2.2\|4.2.2.9 |  |
| FW16_GLEAN_10000141 | 127 | PL3 | Pectate lyase |  | 4.2.2.2 | 4.2.2.2 | 4.2.2.2 |  |
| FW16_GLEAN_10001949 | 128 | PL3 | Pectate lyase | 4.2.2.2 | 4.2.2.2 | 4.2.2.2 | 4.2.2.2 |  |
| FW16_GLEAN_10008463 | 129 | PL3 | Pectate lyase | 4.2.2.2 | 4.2.2.2 | 4.2.2.2 | 4.2.2.2 |  |
| FW16_GLEAN_10012035 | 130 | PL3 | Pectate lyase |  |  | 4.2.2.2 | 4.2.2.2 |  |
| FW16_GLEAN_10008683 | 131 | PL4 | Rhamnogalacturonan lyase |  |  | 4.2.2.23 | 4.2.2.23 |  |
| FW16_GLEAN_10015384 | 132 | PL4 | Rhamnogalacturonan lyase |  |  | 4.2.2.23 | 4.2.2.23 |  |
| FW16_GLEAN_10001628 | 133 | PL9 | Pectate lyase / exopolygalacturonate lyase / rhamnogalacturonan endolyase |  |  |  | 4.2.2.2\|4.2.2.23\|4.2.2.9 |  |
| FW16_GLEAN_10000207 | 134 | PL20 | Endo-β-1,4-glucuronan lyase | 4.2.2.14 | 4.2.2.14 | 4.2.2.14 | 4.2.2.14 |  |
| FW16_GLEAN_10013878 | 135 | PL3;CBM1 | Pectate lyase |  |  | 4.2.2.2 | 4.2.2.2 |  |

## Supplementary Table 5: Quantity of protein applied in the saturation curve experiments.

|  | | Protein load (mg/g of glucan) | | | | | |
| --- | --- | --- | --- | --- | --- | --- | --- |
| Assay | Accellerase 1500 (µL)^(b)^ | | % In-house  cocktail | Protein content (µg/mL) | XSCB | MZ | NSCB |
| A1^(a)^ | 4.1 | | 0 | 0.12 | 4 | 6 | 5 |
| A2 | 4.1 | | 10 | 0.15 | 5 | 7 | 7 |
| A3 | 4.1 | | 25 | 0.19 | 8 | 11 | 10 |
| A4 | 4.1 | | 40 | 0.24 | 12 | 17 | 16 |
| A5 | 4.1 | | 55 | 0.28 | 18 | 25 | 24 |
| A6 | 4.1 | | 70 | 0.32 | 25 | 36 | 35 |

(a) Control assay without *F. metavorans* in-house crude enzymatic extract.

(b) Volume of Accellerase 1500 commercial enzymatic extract at 5 FPU/mL.

## References

White TJ, Bruns T, Lee S, Taylor J (1990) Amplification and direct sequencing of fungal ribosomal

RNA genes for phylogenetics. In: Innis M, Gelfand D, Sninsky J, White T, editors. PCR Protocols: a

Guide to Methods and Applications. Orlando, Florida: Academic Press. pp. 315-322.

Cantarel BL, Coutinho PM, Rancurel C, Bernard T, Lombard V, Henrissat B (2009) The Carbohydrate-Active EnZymes Database (CAZy): An Expert Resource for Glycogenomics. Nucleic Acids Research 37: pp. 233–238.

Lombard V, Ramulu HG, Drula E, Coutinho PM, Henrissat B (2014) The Carbohydrate-Active Enzymes Database (CAZy) in 2013. Nucleic Acids Research 42: pp. 490–495.
